# Machine Learning Accelerated Non‐Adiabatic Molecular Dynamics Elucidates Local Polarization Effects on Non‐radiative Recombination in Halide Perovskites

**DOI:** 10.1002/advs.75903

**Published:** 2026-05-29

**Authors:** Bing Yang, Xiaoli Wei, Bo Cai, Yan Yang, Xinghai Zhu, Yi Liu, Junmin Xia, Lihui Liu, Kun Cao, Wei Shen, Pengfei Xia, Shufen Chen, Siyu Chen, Jin Zhao

**Affiliations:** ^1^ State Key Laboratory of Flexible Electronics (LoFE) & Institute of Advanced Materials (IAM) Nanjing University of Posts & Telecommunications Nanjing China; ^2^ School of Biological Science and Medical Engineering Southeast University Nanjing China; ^3^ Department of Materials Science and Metallurgy University of Cambridge Cambridge UK; ^4^ TCM Group Cavendish Laboratory University of Cambridge Cambridge UK; ^5^ Department of Physics and ICQD/Hefei National Research Center for Physical Sciences at the Microscale University of Science and Technology of China Hefei China; ^6^ Hefei National Laboratory University of Science and Technology of China Hefei China; ^7^ Department of Physics and Astronomy University of Pittsburgh Pittsburgh USA

**Keywords:** excited‐state dynamics, local polarization, machine learning, non‐adiabatic molecular dynamics

## Abstract

Non‐radiative recombination is a critical factor limiting the optoelectronic performance of halide perovskites, yet how local polarization induced by charge redistribution regulates this process remains unclear. To gain deeper insight while reducing the high computational cost of conventional non‐adiabatic molecular dynamics (NAMD), we developed Hefei‐NAMD‐S, a machine learning (ML) framework constructed using stacked models. The relative errors of the ML predicted non‐adiabatic coupling and pure‐dephasing time, calculated with respect to the first‐principles values, remain below 1.10%, while the total computational time is reduced by approximately 78%, demonstrating the accuracy and efficiency of the proposed framework. NAMD simulations further reveal that the B‐site local polarization is involved in regulating the non‐radiative recombination process. The rubidium‐substitution‐doped system (FA_Rb_) and the cesium interstitial doped system exhibit recombination times of about 280 ns, nearly 2.8 times that of the pristine system, and the enhanced performance of FA_Rb_ has been supported by previous experimental evidence. These results identify B‐site local polarization as one of the important factors in suppressing non‐radiative recombination and provide a theoretical foundation for designing perovskite materials through local polarization modulation.

## Graphical abstract

This work proposes and constructs the Hefei‐NAMD‐S framework based on machine learning stacked models to investigate the relationship between local polarization and non‐radiative recombination. The results indicate that, compared with A‐site local polarization, B‐site local polarization shows a more evident association with the non‐radiative recombination process.

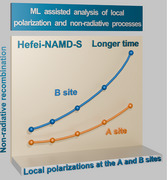

## 1 Introduction

Halide perovskites have emerged as highly promising optoelectronic materials due to their excellent light absorption capability, long carrier diffusion lengths, and favorable charge transport properties [1, 2, 3]. Their remarkable performance has enabled rapid progress in diverse applications such as solar cells, light‐emitting diodes, and photodetectors [4, 5, 6, 7]. At present, the certified power conversion efficiency (PCE) of perovskite solar cells has reached 27.30% [8], exhibiting competitiveness comparable to that of commercial silicon‐based photovoltaics. Nevertheless, despite their outstanding optoelectronic properties, the device performance of perovskites remains constrained by non‐radiative recombination, which constrains both device stability and achievable open‐circuit voltage (V_OC_) [9, 10, 11, 12, 13, 14, 15]. Non‐radiative recombination is fundamentally an excited‐state dynamical process driven by non‐adiabatic interactions between electrons and lattice vibrations. It involves electronic transitions among multiple potential energy surfaces, as well as energy exchange between electrons and atomic nuclei, making it difficult to accurately describe using only the coherence loss of a static electronic subsystem [16, 17, 18]. Therefore, a deep understanding of the dynamical mechanisms governing non‐radiative recombination in halide perovskites is crucial for the rational design and optimization of high‐performance optoelectronic materials.

Although extensive discussions have been conducted on the non‐radiative recombination processes in perovskite materials, most existing studies have primarily focused on the influence of defect‐state distributions [19, 20, 21], phase transitions [22, 23, 24], octahedral distortions [25, 26, 27], and the rotational or dynamic disorder of A‐site organic cations [28, 29]. These lattice perturbations in perovskites are often accompanied by charge redistribution and are highly likely to induce polarization, thereby modulating electron–phonon coupling and the non‐radiative recombination process. Previous studies, at a more macroscopic structural scale, have demonstrated that grain‐boundary polarization can regulate non‐radiative recombination. For example, Li and coworkers showed that the grain‐boundary polarization present at self‐stabilizable deformed domain walls promotes charge separation and suppresses non‐radiative recombination [30]. Ping and colleagues further found that polar grain boundaries can spontaneously form even in nominally nonpolar systems, and such polarization effectively prolongs carrier lifetimes by regulating the spatial separation of electrons and holes [31]. These studies highlight the significant role of grain‐boundary polarization in modulating excited‐state dynamics. However, for polarization at the ionic scale, particularly the local polarization around A‐ and B‐ site cations that are induced by lattice perturbations in perovskites, excited‐state dynamical evidence regarding how such local polarization influences the non‐radiative recombination process remains lacking. Exploring non‐radiative recombination from the perspective of local polarization may therefore provide a valuable and previously underexplored understanding of excited‐state dynamics in perovskite materials.

With the continuous advancement of excited‐state theories and computational methods, non‐adiabatic molecular dynamics (NAMD) has become an important simulation approach for investigating hot carrier relaxation, energy conversion, and time‐resolved photoluminescence processes [18, 32, 33, 34, 35]. NAMD enables direct visualization of population evolution among different electronic state potential energy surfaces and provides essential dynamical information for analyzing non‐radiative recombination pathways and rates. However, the practical application of NAMD remains challenging due to its extremely high computational demands. Conventional NAMD simulations require extensive electronic structure calculations and non‐adiabatic coupling (NAC) evaluations along dynamical trajectories, and the time‐dependent Schrödinger equation often needs to be solved tens of thousands of times [18, 19, 36, 37]. The resulting high computational cost makes it difficult to employ more accurate hybrid functionals, larger systems, or longer simulation times, thereby limiting comprehensive exploration of the microscopic mechanisms underlying non‐radiative recombination [38, 39, 40, 41]. The development of artificial intelligence in recent years has created new opportunities to alleviate the computational resource bottlenecks in first‐principles calculations. In particular, machine learning (ML) accelerated NAMD strategies have attracted considerable attention [42, 43, 44, 45, 46]. For example, Chu and coworkers employed E3‐equivariant neural networks to predict Hamiltonians and achieved efficient and accurate prediction of NAC and excited‐state energies [32]. The selection of effective descriptors and algorithms has thus become a key factor in the development of ML‐accelerated NAMD methodologies.

In this work, a prediction framework based on stacked models was constructed to accelerate the acquisition of input parameters for the Hefei‐NAMD package, hereafter referred to as Hefei‐NAMD‐S. This framework primarily focuses on formamidinium lead iodide (FAPbI_3_) and related perovskite systems doped with alkali metals (Potassium, K; Rubidium, Rb; Cesium, Cs), and titanium dioxide systems are included as complementary cases to validate the reliability and generalizability of the framework. For both NAC and pure‐dephasing time, the differences between the Hefei‐NAMD‐S predictions and the calculated values remain within 1.10%, and the framework exhibits strong generalizability for titanium dioxide. Compared with simulations conducted without Hefei‐NAMD‐S, this framework reduces central processing unit (CPU) runtime by approximately 78% without compromising accuracy, thereby substantially improving simulation efficiency. Quantitative analysis reveals that the B‐site generally exhibits stronger local polarization than the A‐site. Although the influence of A‐site polarization is comparatively weak, its contribution cannot be ignored. Subsequent NAMD simulations demonstrate that the non‐radiative recombination times of the rubidium substitution doped (FA_Rb_), and the cesium interstitial doped (Cs_i_) systems reach about 280 ns, nearly 2.8 times longer than that of the pristine lattice. Notably, the enhanced performance of the FA_Rb_ system has been validated experimentally. Furthermore, this study suggests that B‐site local polarization may serve as one of the important factors regulating the non‐radiative recombination process, as indicated by its association with the non‐radiative recombination time in most of the studied systems. This finding not only deepens the understanding of excited‐state dynamical mechanisms in perovskites but also offers a new theoretical perspective for designing perovskite materials through local polarization regulation.

## 2 Results and Discussion

### 2.1 Hefei‐NAMD‐S Framework

**FIGURE 1:**
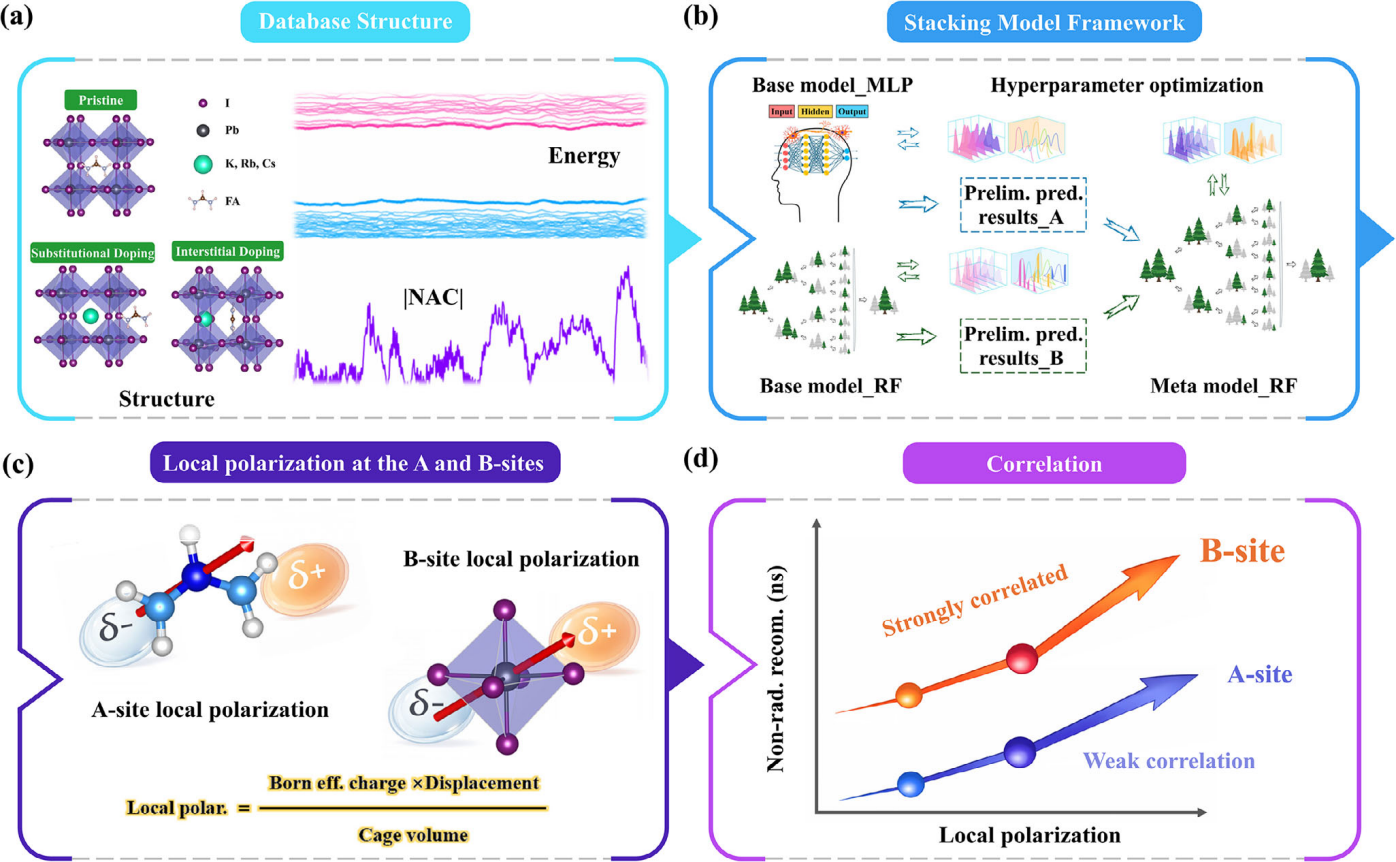
Workflow for the accelerated analysis of the effects of local polarization on non‐radiative recombination processes enabled by the Hefei‐NAMD‐S framework, (a) database structure; (b) stacking model framework; (c) local polarization at the A‐ and B‐ sites; and (d) correlation between local polarization and non‐radiative recombination time.

Before employing ML for NAMD acceleration, database construction is an essential preparatory steps. As shown in Figure 1a, we extracted 2000 time steps from adiabatic molecular dynamics trajectories of 2 × 2 × 2 supercells, including pristine FAPbI_3_, alkali metal substitutionally doped systems formed by substituting one FA cation with one alkali metal ion at the A‐site of FAPbI_3_ (FA_K_, FA_Rb_, and FA_Cs_), and interstitially doped systems formed by incorporating one alkali metal ion at an interstitial site (K_i_, Rb_i_, and Cs_i_). To capture both structural and dynamical information of these perovskite crystals, the database utilizes atomic coordinates, atomic forces, bond lengths, and energies as input descriptors. The valence band maximum (VBM), conduction band minimum (CBM), and the absolute value of the NAC factor (|NAC|) are selected as target properties. To overcome the limitations of a single model in terms of generalization ability and predictive performance, we constructed the Hefei‐NAMD‐S framework based on a stacked model. This framework enables rapid acquisition of the input parameters required for NAMD simulations, and its output can be directly used as input files for the Hefei‐NAMD software package [47, 48, 49, 50, 51], thereby avoiding time‐consuming post‐processing procedures. At the core of the Hefei‐NAMD‐S framework lies the construction of the stacked model. Random forest (RF) [52, 53] and multilayer perceptron (MLP) [54, 55] algorithms were selected as base models, while RF was also employed as the meta model, as shown in Figure 1b. In the stacked model, the meta model performs a secondary prediction based on the outputs of the base models to produce the final results. In this process, the training inputs for the meta model were constructed from the out‐of‐fold predictions generated during five‐fold cross‐validation, thereby avoiding potential information leakage during the stacking process. Meanwhile, this strategy ensured the independence between the train set (D_Train_) and the test set (D_Test_). Additional details regarding the ML preparatory procedures and Hefei‐NAMD‐S framework are provided in Sections S1.1–S1.6.

Local polarization is quantitatively characterized by combining the Born effective charge tensor, ionic displacements, and the cage volume, thereby capturing changes in polarization strength induced by lattice distortion and charge redistribution. As shown in Figure 1c, the A‐site local polarization primarily arises from the off‐centering displacement of organic and alkali metal cations within the cage, whereas the B‐site local polarization is mainly governed by distortions of the octahedral framework. Based on this physical definition, local polarization is introduced as a physically interpretable descriptor that bridges the electronic structure information accelerated by the Hefei‐NAMD‐S framework with non‐adiabatic molecular dynamics studies. Furthermore, as illustrated in Figure 1d, correlating the A‐ and B‐ site local polarization strengths with the non‐radiative recombination times obtained from non‐adiabatic molecular dynamics simulations enables a systematic investigation of the relationship between local polarization and non‐radiative recombination dynamics, thereby providing a basis for the subsequent results and mechanistic analysis. Additional details regarding the principles of the computational procedures of NAMD are provided in Sections S1.7.

### 2.2 Hefei‐NAMD‐S Prediction Accuracy and Computational Time

**FIGURE 2:**
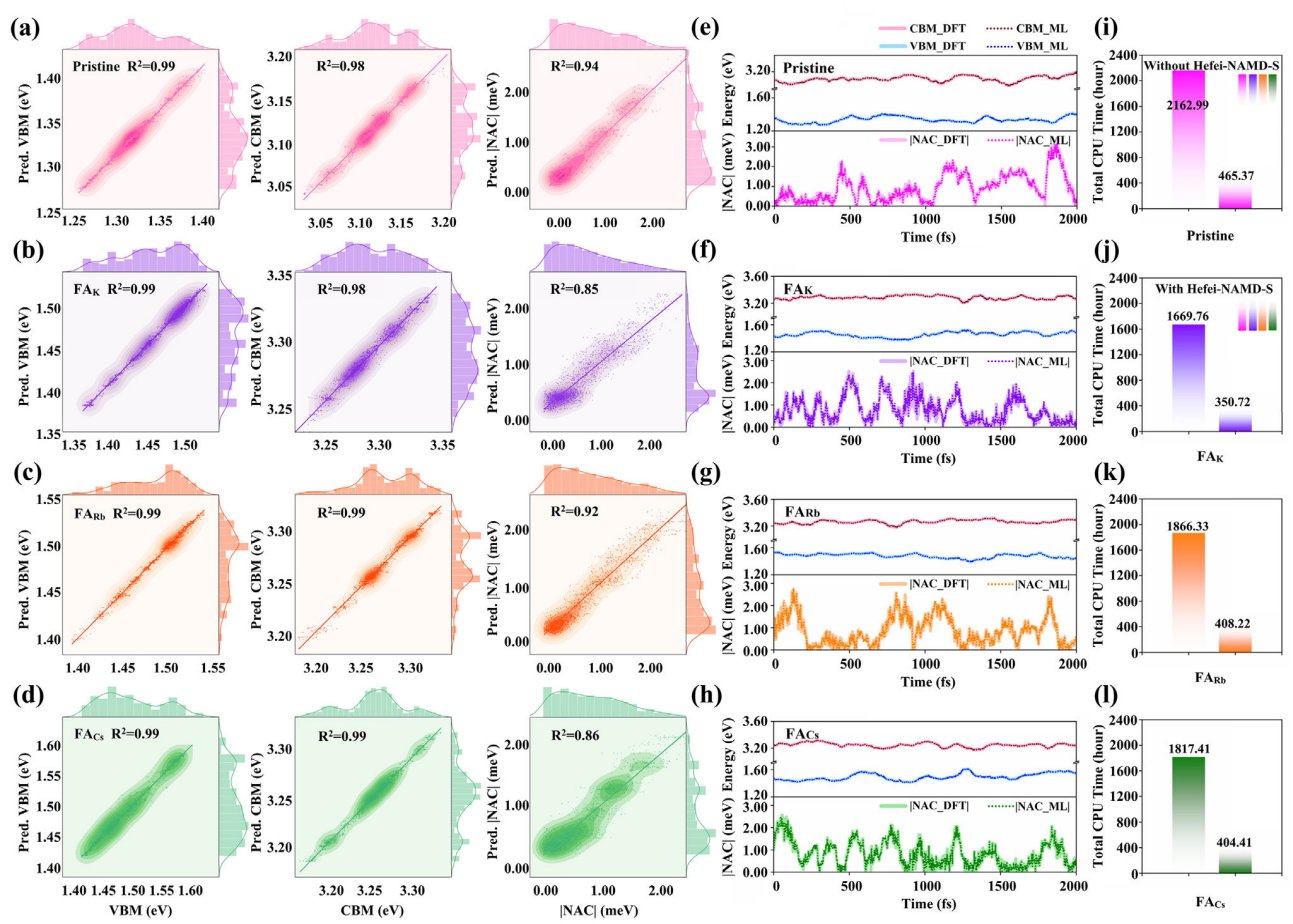
Performance of the machine learning stacked model on the test set, (a–d) kernel density plots illustrating the relationship between predicted and calculated values; (e–h) evolution of the CBM and VBM (upper panels) and |NAC| (lower panels) over a 2000 fs trajectory, where solid lines represent calculated values and dashed lines represent predicted values; (i–l) comparison of total CPU time consumption between simulations with and without the Hefei‐NAMD‐S framework, presented as histograms.

To evaluate the predictive capability of the stacked model, we analyzed the D_Test_ results for pristine and substitution‐doped FAPbI_3_ systems, as shown in Figure 2. Figure 2a–d shows kernel density distributions comparing the calculated and predicted values of VBM, CBM, and |NAC|. In these plots, the scatter points denote individual data samples, whereas the color shading and marginal histograms reflect the corresponding data densities. The coefficient of determination (R^2^) between the predicted and calculated values of the orbital energies is approximately 0.98, and the regression lines for the VBM and CBM almost overlap with the diagonal, indicating a high level of agreement between the predicted and reference values. In contrast, the |NAC| exhibits a slight degree of dispersion, with the lowest R^2^ being 0.85. This behavior is mainly attributed to the structural perturbations and complex wavefunction variations induced by doping, which increase the difficulty of accurately predicting the |NAC|. Nevertheless, the predictive performance of the stacked model for the |NAC| still surpasses that reported in previous studies [45, 56]. In addition, we further verified the generalizability of the model using the non‐perovskite titanium dioxide systems [57], and a more comprehensive evaluation of the prediction metrics is provided in Table S7. Subsequently, according to the equally spaced sampling rule used for data extraction, the predicted values corresponding to D_Train_ and D_Test_ were reinserted into the dynamical trajectory in their original temporal order. This procedure reconstructed a complete and continuous trajectory, from which the time evolution of the VBM, CBM, and |NAC| over a timescale of 2000 fs was obtained, as shown in Figure 2e–h. The evolution trajectories reconstructed from the predicted VBM and CBM values (dashed lines) remain consistently enclosed within those obtained from density functional theory (DFT) calculations (solid lines), with maximum deviations below 0.10 eV (see Figure S22), further demonstrating the high accuracy of the stacked model in predicting orbital energies. For |NAC|, the predicted trajectory shows reduced amplitude due to the underestimation of extrema. Combined with the analysis presented in Figures S22, over 83% of the D_Test_ prediction errors are less than 0.30 meV and still demonstrating robust generalization. Moreover, based on the comparisons between the evolution curves and the difference distributions, the potential risk of underfitting or overfitting in the stacked model can be effectively ruled out.

After evaluating the predictive performance of the stacked model, we further investigated the non‐adiabatic properties and analyzed the accuracy of the non‐adiabatic data obtained with and without the Hefei‐NAMD‐S framework. As summarized in Table S8, the maximum difference in NAC values remains below 0.003 meV for both the pristine and the substitution‐doped systems. In the substitutionally doped systems, the differences in the pure‐dephasing time obtained from the two simulation schemes are all below 0.10 fs, with a slightly larger difference of approximately 0.18 fs observed only in the pristine system. Overall, the results obtained from the Hefei‐NAMD‐S framework are in excellent agreement with those of the Hefei‐NAMD package, with relative deviations below 1.10%, demonstrating the high predictive accuracy of the framework and ensuring the reliability of subsequent NAMD simulations. Figure 2i–l presents a comparison of the total CPU time required by the two simulation approaches. All simulations were performed using 52 cores on Intel(R) Xeon(R) Gold 6278C processors running at 2.60 GHz. The statistical results indicate that approximately 78% of the computational time was saved in the pristine and substitutional doped systems using the stacked model approach. In addition, the use of the stacked model markedly decreases the demand for storage resources by eliminating the need to generate large intermediate files, thereby alleviating the associated hardware burden. Overall, the Hefei‐NAMD‐S framework constructed in this work not only ensures high predictive accuracy but also achieves substantial improvements in computational efficiency and resource utilization. More importantly, it provides a reliable foundation for establishing the correlation between A‐ and B‐ site local polarization and non‐radiative recombination in the subsequent analysis.

### 2.3 Electronic Structure

**FIGURE 3:**
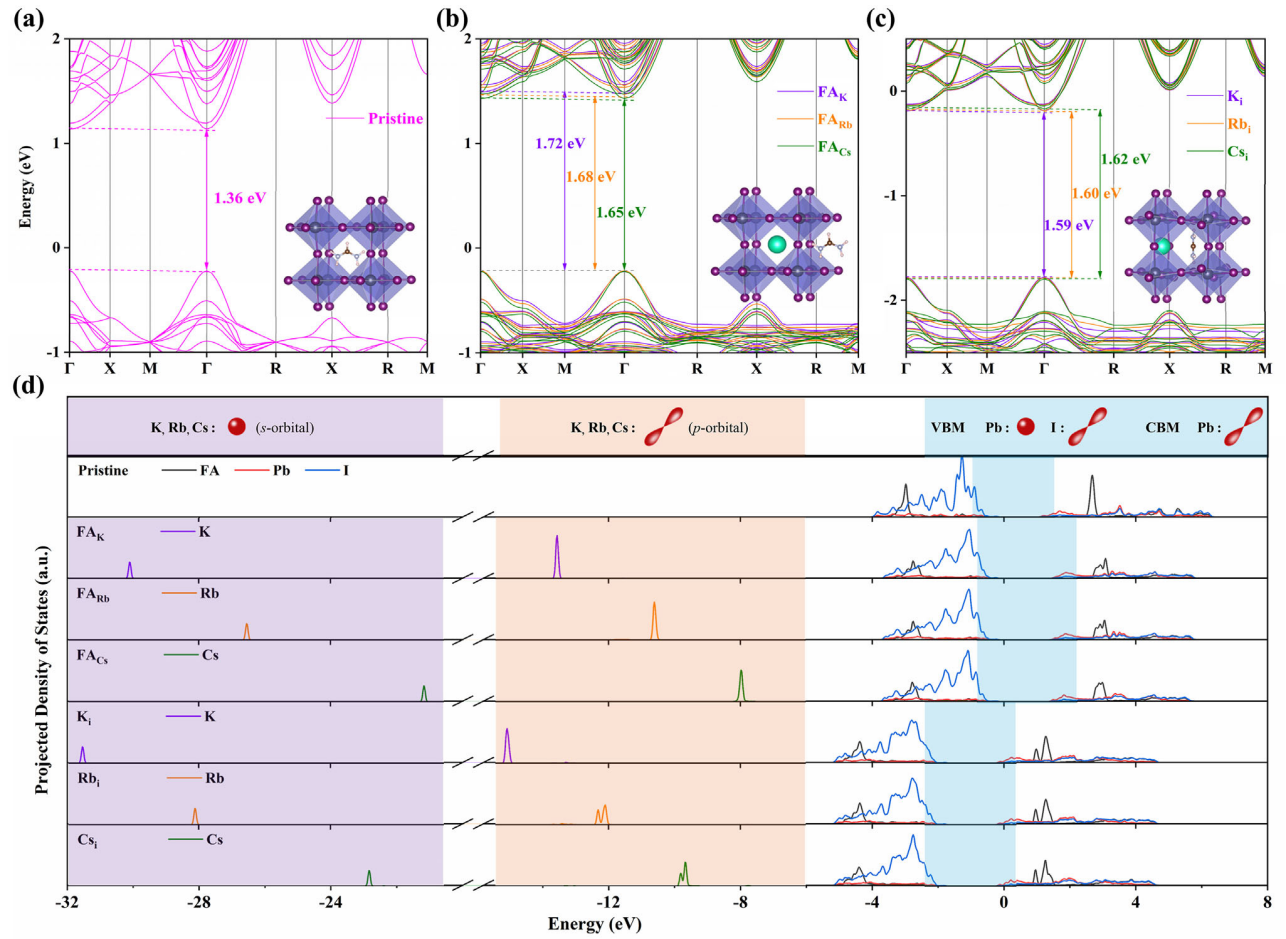
Band structures of halide perovskites for (a) the pristine system; (b) the substitutional doped system; (c) the interstitial doped system; and (d) projected density of states, where the purple, orange, and blue regions indicate the contributions of alkali metal *s*‐orbitals, *p*‐orbitals, and Pb/I atoms at the band edges, respectively. For each system, the energy zero is referenced to its own Fermi level.

Before investigating non‐radiative recombination, electronic structure analysis is essential for understanding the properties of the systems. First, structural optimization of the perovskite was performed at 0 K, and the optimized crystal structures along with the corresponding lattice parameters are shown in Figure S23 and Table S9. For each system, the energy zero is referenced to its own Fermi level. Figure 3a–c presents the band structure of the FAPbI_3_, and its alkali metal doped systems. The bandgap of the pristine system obtained from DFT calculations is 1.36 eV, in good agreement with the experimental value of 1.48 eV [58, 59, 60]. Moreover, a difference of approximately 0.10 eV between the theoretical and experimental band gaps is also considered reasonable [3, 61]. Notably, the band gaps of all doped systems are larger than that of the pristine system. According to crystallographic databases, the ionic radii of alkali metals K, Rb, and Cs are 1.64, 1.72, and 1.88 Å, respectively [62]. In the substitutional doped systems, the bandgap gradually decreases with increasing alkali metal ionic radius. In contrast, in the interstitial doped systems, the bandgap tends to increase as the ionic radius increases. These variations in the bandgap are attributed to differences in the extent of local lattice distortion, which is induced by the varying ionic radii of the alkali metals. Moreover, all perovskite systems exhibit a direct bandgap at the Γ point. The detailed methodology of the first‐principles simulations is provided in Section S2.1.

Subsequently, we analyzed the atomic and orbital contributions to energy levels, as shown in Figure 3d and Figure S31–S37. In these figures, the blue regions indicate that the VBM is mainly contributed by Pb‐*s* and I‐*p* orbitals, while the CBM is primarily composed of Pb‐*p* orbitals. In contrast, the contribution of the FA cation is mainly located at deeper energy levels, far from the band edges. The contributions from the alkali metal ions are more widely distributed, with the *s* and *p*‐orbital of the alkali metals appearing as purple and orange regions, respectively, in Figure 3d. Moreover, the contributions from the *s* and *p*‐orbitals gradually shift toward shallower energy levels as the ionic radius of the alkali metal increases. Although A‐site cations do not directly determine the band edge, they can induce short‐range interactions with the local Pb‐I lattice through hydrogen bonding and van der Waals forces, leading to localized lattice distortions that indirectly modulate the band. Moreover, due to their polar, A‐site cations may also exert long‐range interactions with charge carriers, thereby affecting their spatial distribution and regulating the non‐radiative recombination process.

### 2.4 Orbital Energy Evolution and Partial Charge Density

**FIGURE 4:**
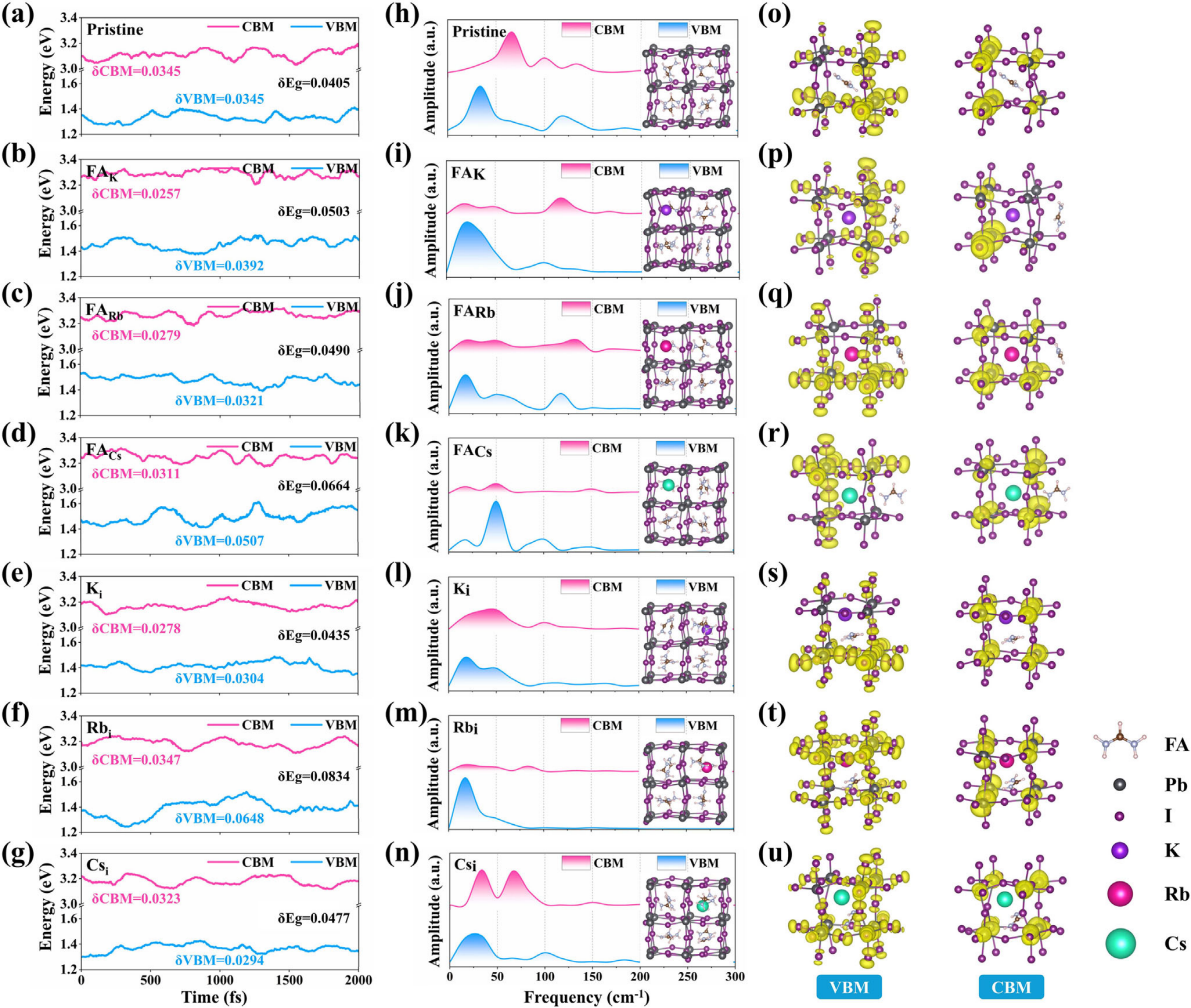
Based on predictions from the stacked model, (a–g) the evolution of Kohn–Sham orbital energies during 2000 fs, with the corresponding standard deviations of the CBM, VBM, and Eg fluctuations indicated in each panel (in eV); (h–n) the Fourier transforms of the CBM and VBM autocorrelation functions, with insets showing representative crystal structures extracted after completion of the 300 K heating process; and (o–u) partial charge density distributions of the VBM and CBM calculated using the corresponding representative 300 K structures.

Based on the above analysis of the electronic structures of the perovskite systems at 0 K, we subsequently heated each system to 300 K in the canonical ensemble, with the detailed heating process shown in Figure S39. Figure 4a–g shows the time evolution of the Kohn–Sham orbital energies along the 2000 fs dynamical trajectory. These results indicate that thermal lattice motion continuously modulates the band edge electronic states, causing the band edge energies and instantaneous band gap to exhibit different degrees of dynamic fluctuation. To compare the fluctuation amplitudes among different systems, we calculated the standard deviations of the band edge energies and band gap, denoted as δCBM, δVBM, and δEg, respectively. The detailed calculation procedure is provided in Section S2.2.

Figure 4b–d shows the evolution of the band edge energies and their corresponding standard deviations for the substitutionally doped systems with different alkali metal ions. Among them, the FA_Cs_ system exhibits the most pronounced fluctuation amplitudes, with δCBM, δVBM, and δEg values of 0.0311, 0.0507, and 0.0664 eV, respectively, indicating that substitutional doping with Cs ions induces the strongest perturbation of the band edge electronic states. In contrast, the FA_Rb_ system shows relatively smaller fluctuation amplitudes and weaker perturbation of the band edges, with δCBM, δVBM, and δEg values of 0.0279, 0.0321, and 0.0490 eV, respectively. Combined with the Fourier transform spectra shown in Figure 4j, the FA_Rb_ system exhibits relatively weak coupling between the band edge energies and the low‐frequency inorganic Pb‐I framework vibrations. Specifically, the low‐frequency peak near 30 cm^−1^ is typically associated with PbI_6_ octahedral distortion, the peak near 100 cm^−1^ mainly originates from Pb‐I bending and stretching vibrations, and the weak peaks in the 150–200 cm^−1^ region are generally related to the rotational or torsional motions of the FA organic cations [63, 64]. This relatively weak low‐frequency phonon coupling may help reduce the electron–phonon coupling strength, thereby improving the carrier lifetime. This result is consistent with the performance enhancement observed in previous experimental studies on Rb ion regulation in FAPbI_3_ perovskites [65, 66]. Wu et al. reported that the introduction of Rb ions can effectively reduce the defect density in FAPbI_3_ perovskites and suppress the non‐radiative recombination process. Accordingly, the device PCE increased from 23.26% to 25.14%, while V_OC_ and the fill factor (FF) improved to 1.184 V and 84.76%, respectively [66]. Further combining the partial charge densities shown in Figure 4p–r, it can be seen that the FA_Rb_ system does not exhibit the strongest electron wavefunction overlap, which may be related to its weaker band edge energy fluctuations and weaker low‐frequency phonon coupling.

By comparison, the interstitially doped systems exhibit different dynamical fluctuation characteristics. As shown in Figure 4e–g, the Rb_i_ system exhibits the strongest band edge energy and band gap fluctuations, with δCBM, δVBM, and δEg values of 0.0347, 0.0648, and 0.0834 eV, respectively, which are significantly higher than those of the K_i_ and Cs_i_ systems. This indicates that interstitial doping with Rb ions induces stronger dynamic perturbations of the band edge electronic states. Similarly, the Fourier transform spectra shown in Figure 4l–n reveals that the VBM of the Rb_i_ system exhibits more pronounced low‐frequency vibrations. These low‐frequency modes are mainly associated with PbI_6_ octahedral distortion and can directly modulate the band edge electronic states [63, 64]. Further analysis of the partial charge densities shown in Figure 4s–u indicates that the Rb_i_ system exhibits the largest electron wavefunction overlap between the VBM and CBM, which can enhance electron‐hole interactions and facilitate larger NAC values, as shown in Table 1. However, the Cs_i_ system exhibits a certain degree of complexity. Figure 4n shows that its CBM has relatively strong low‐frequency phonon coupling around 40 and 65 cm^−1^. At the same time, this system is characterized by relatively small band edge energy and band gap fluctuation amplitudes, together with a moderate degree of electron wavefunction overlap. Therefore, the non‐radiative recombination process in the Cs_i_ system may be competitively regulated by multiple physical mechanisms and require further investigation.

**TABLE 1:** In the pristine and doped systems, the local polarizations at the A‐ and B‐sites (µC/cm^2^), the non‐adiabatic couplings (meV), the pure‐dephasing time (fs), and the non‐radiative electron‐hole recombination time (ns) are presented.

		Pristine	FA_K_	FA_Rb_	FA_Cs_	K_i_	Rb_i_	Cs_i_
Local Polarization	A‐site	0.18	0.63	0.50	0.31	0.94	1.48	1.73
	B‐site	5.41	2.87	3.08	2.36	3.06	5.72	6.37
NAC		0.31	0.26	0.29	0.27	0.27	0.37	0.23
Pure‐dephasing Time		16.55	13.26	13.49	9.98	15.36	7.90	13.94
Non‐radiative Recombination Time		99.86	156.93	280.80	91.16	97.36	31.83	271.59

### 2.5 Relationship Between Local Polarization and Non‐radiative Recombination

Alkali metal doping can induce the displacement of A‐site cations and further trigger lattice distortion of the inorganic Pb‐I octahedra. The displacement of A‐site cations can modify the carrier distribution in the local environment, whereas the distortion of Pb‐I octahedra more directly affects the distribution of band edge electronic states. Therefore, alkali metal doping can regulate the carrier distribution around both A‐ and B‐sites, thereby altering the local polarization strength. To quantitatively evaluate these effects, we calculated the local polarization at both the A‐ and the B‐site based on the Born effective charge tensor, ionic displacements, and cage volume. This approach captures short‐range dipole variations arising from lattice distortions and charge redistribution, and the corresponding computational details are provided in Section 2.3. In addition, to avoid the influence of instantaneous local polarization caused by thermal fluctuations, the ionic positions in the 300 K dynamical trajectories were averaged [31]. This averaging treatment can reduce short‐lived or instantaneous fluctuations while preserving long‐term or persistent ionic displacements and distortion behaviors. Therefore, it does not weaken the overall contribution of structural fluctuations to local polarization.

**FIGURE 5:**
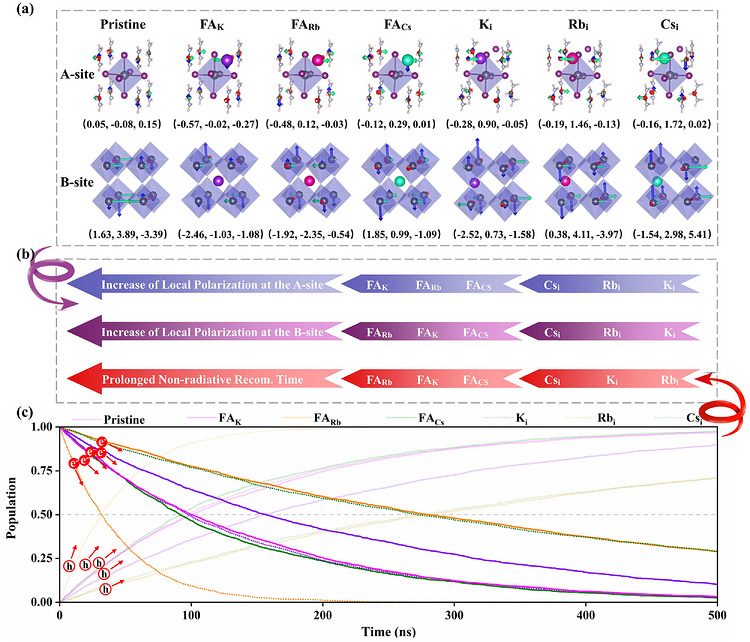
The pristine and doped systems include (a) the components of local polarization at the A‐ and B‐sites along the *x*, *y*, and *z* directions; (b) the relationship between local polarization and non‐radiative recombination time; and (c) the evolution of electron and hole populations during the non‐radiative recombination process.

As shown in Table 1, the A‐site local polarization in the substitutionally doped systems exhibits a trend opposite to the ionic radius of the alkali metals. This behavior may arise from the increased spatial occupation of the A‐site cation, which reduces the polarization intensity per unit volume. In the interstitial doped systems, as illustrated in Figure 5a, the displacement of the A‐site cation increases with the ionic radius of the dopant, leading to an enhancement in local polarization. Meanwhile, we also note that the magnitude of the A‐site local polarization is generally small across all systems. In contrast, the B‐site polarization is significantly larger in both magnitude and variation, indicating higher sensitivity to structural perturbations. Unlike the A‐site, the B‐site local polarization does not exhibit a monotonic trend with respect to the ionic radius of the alkali metal dopant. This is likely because A‐site doping does not directly perturb the B‐site; instead, it influences the PbI_6_ octahedral framework and thereby modifies the B‐site polarization indirectly. Among the systems studied, FA_Rb_ and Cs_i_ exhibit the highest B‐site local polarization in the substitutional and interstitial doping, respectively. Overall, changes in carrier distribution may enhance local polarization and generate local potential differences, thereby further influencing the non‐radiative recombination process. Notably, compared with the A‐site, the B‐site exhibits stronger local polarization and is more sensitive to lattice perturbations. Therefore, local polarization, particularly associated with the B‐site, should be considered as one of the important factors when interpreting the differences in excited‐state dynamics among perovskite systems.

Although we have inferred that local polarization may be a potential factor influencing the non‐radiative recombination process, further validation is still required. To this end, we performed NAMD simulations on FAPbI_3_ and its alkali metal‐doped systems, followed by a comprehensive analysis of the excited‐state dynamics by NAC and pure‐dephasing time. NAC reflects the inelastic scattering associated with energy exchange between two subsystems, whereas elastic scattering is described by the pure‐dephasing time [18, 46]. As shown in Table 1, the combination of a relatively small NAC value of 0.23 meV and a fast pure‐dephasing time of 13.94 fs makes Cs_i_ a promising perovskite system, comparable to FA_Rb_. Figure 5b further illustrates the relationship between local polarization and non‐radiative recombination time, constructed by directly correlating the results presented in Figure 5a,c. In the substitutionally doped systems, the non‐radiative recombination time increases markedly with the enhancement of B‐site local polarization. Among these systems, FA_Rb_ achieves an exceptionally long non‐radiative recombination time of 280.80 ns, along with a polarization strength of 3.08 µC/cm^2^. The beneficial role of Rb substitution in suppressing non‐radiative recombination has been confirmed in numerous experimental and theoretical studies [65, 66, 67]. A similar relationship is observed in the interstitial doped systems. The Cs_i_ system, which exhibits a B‐site polarization strength of 6.37 µC/cm^2^, shows a non‐radiative recombination time of 271.59 ns, which is considerably longer than that of the pristine system, as shown in Figure 5c. Despite the different doping configurations, both FA_Rb_ and Cs_i_ develop pronounced B‐site local polarization, and both systems substantially prolong the non‐radiative electron‐hole recombination time to approximately 280 ns, nearly 2.8 times that of the pristine lattice. Overall, systems exhibiting stronger B‐site local polarization tend to show longer non‐radiative recombination times. However, the influence of A‐site local polarization on the non‐radiative process cannot be denied; it is simply weaker in magnitude than that of the B‐site. These results suggest that local polarization is one of the important factors influencing the non‐radiative recombination process, with B‐site polarization showing a more evident association with non‐radiative recombination behavior. This view is supported by the experimental study of Qin et al. on the twinning tetragonal phase of perovskite, where nonuniform strain‐induced lattice polarization near distorted domain walls promoted charge separation, thereby contributing to the suppression of non‐radiative recombination and a significant enhancement in the device V_OC_ [30]. Elucidating this association not only provides a more comprehensive understanding of excited‐state dynamics in perovskite systems but also establishes a theoretical basis for material design through local polarization modulation.

## 3 Conclusion

In summary, this work focuses on addressing the challenges of computational resources and time costs in NAMD simulations by proposing and constructing the Hefei‐NAMD‐S prediction framework based on stacked models. FAPbI_3_ and its alkali metal‐doped perovskites were selected as research systems. The difference in NAC values between simulations with and without the Hefei‐NAMD‐S framework remains within 0.003 meV, while that in pure‐dephasing time is generally below 0.10 fs. Overall, all discrepancies are confined within 1.10% of the results obtained from the Hefei‐NAMD package. While achieving comparable accuracy, the Hefei‐NAMD‐S framework reduces computational cost and runtime by approximately 78%. In addition, when applied to non‐perovskite systems, the framework still exhibits promising predictive capability, further demonstrating its comprehensive advantages in terms of accuracy, stability, and generalizability. Beyond the methodological advances, this study further elucidates the regulatory role of A‐ and B‐site local polarization in the excited‐state dynamics of perovskites. Alkali metal doping disrupts the symmetry of the crystal structure, inducing lattice distortions and charge redistribution, which in turn lead to variations in the local polarization around both the A‐ and the B‐site cations. Quantitative analysis shows that B‐site local polarization exhibits higher intensity and greater sensitivity to structural perturbations than that at the A‐site; however, this does not exclude the contribution of A‐site local polarization to the non‐radiative recombination process, whose influence is simply weaker by comparison. Further NAMD simulations indicate that systems with stronger B‐site local polarization generally tend to exhibit longer non‐radiative recombination time. Both FA_Rb_ and Cs_i_ exhibit long non‐radiative recombination times of approximately 280 ns, nearly 2.8 times that of the pristine system, and previous experimental studies have also confirmed that Rb ion regulation improves the non‐radiative recombination time as well as photovoltaic parameters, including V_OC_ and FF. Overall, this work suggests that B‐site local polarization is one of the important factors influencing non‐radiative recombination in halide perovskites. This insight not only deepens the understanding of excited‐state dynamical mechanisms in these materials but also provides a theoretical foundation for designing perovskite systems through local polarization modulation.

## Author Contributions

**Bing Yang**: data curation, writing – original draft, formal analysis, methodology. **Xiaoli Wei**: data curation, validation, formal analysis, writing – original draft. **Yi Liu**: formal analysis. **Bo Cai**: writing – review and editing, conceptualization, methodology, funding acquisition, supervision, project administration. **Wei Shen**: formal analysis. **Yan Yang**: formal analysis. **Lihui Liu**: formal analysis. **Shufen Chen**: writing – review and editing, conceptualization, funding acquisition, supervision. **Junmin Xia**: formal analysis. **Xinghai Zhu**: formal analysis. **Kun Cao**: formal analysis. **Pengfei Xia**: formal analysis. **Jin Zhao**: writing – review and editing, conceptualization, methodology, software, supervision. **Siyu Chen**: conceptualization, methodology, supervision, writing – review and editing, project administration, funding acquisition.

## Conflicts of Interest

The authors declare no conflicts of interest.

## Supporting information




**Supporting File**: advs75903‐sup‐0001‐SuppMat.docx.

## Data Availability

The data that support the findings of this study are available from the corresponding author upon reasonable request. The code used in the current study is available at GitHub (https://github.com/BingYang‐NJUPT/Hefei‐NAMD‐S)
