# Supplementary material for: Machine Learning Accelerated Non‐Adiabatic Molecular Dynamics Elucidates Local Polarization Effects on Non‐radiative Recombination in Halide Perovskites
Source: Adv Sci (Weinh). 2026 May 29:e75903. Online ahead of print. doi: 10.1002/advs.75903 (PMC13336369; doi:10.1002/advs.75903)
Supplement: Supplementary file 1 — Supporting File: advs75903‐sup‐0001‐SuppMat.docx. [file ADVS-9999-e75903-s001.docx]

**Supporting Information**

**Machine Learning Accelerated Non-adiabatic Molecular Dynamics Elucidates Local Polarization Effects on Non-radiative Recombination in Halide Perovskites**

*Bing Yang, Xiaoli Wei, Bo Cai^*^, Yan Yang, Xinghai Zhu, Yi Liu, Junmin Xia, Lihui Liu, Kun Cao, Wei Shen, Pengfei Xia, Shufen Chen^*^, Siyu Chen^*^ and Jin Zhao*

E-mail: [iambcai@njupt.edu.cn](mailto:iambcai@njupt.edu.cn); [iamsfchen@njupt.edu.cn](mailto:iamsfchen@njupt.edu.cn); sc2090@cam.ac.uk

B. Y., X. W., and B. C. contributed equally to this work.

**Contents**

[Section S1: Details of the Hefei-NAMD-S Framework 3](#_Toc214979162)

[Section S1.1: Database Structure 3](#_Toc214979163)

[Section S1.2: Feature Descriptors and Target Properties 4](#_Toc214979164)

[Section S1.3: Feature Engineering 5](#_Toc214979165)

[Section S1.4: Hefei-NAMD-S Framework 19](#_Toc214979166)

[Section S1.5: Equations of the Hefei-NAMD-S Framework 22](#_Toc214979167)

[Section S1.6: Scoring Methods for Evaluation Metrics 23](#_Toc214979168)

[Section S1.7: Non-adiabatic Molecular Dynamics Simulations 25](#_Toc214979169)

[Section S2: First-principles Calculation 27](#_Toc214979170)

[Section S2.1: Computational Methods for Electronic Structure 27](#_Toc214979171)

[Section S2.2: Computational Methods for Non-adiabatic Properties 36](#_Toc214979172)

[Section S2.3: Computational Methods for Local Polarization 42](#_Toc214979173)

[References 43](#_Toc214979174)

# Section S1: Details of the Hefei-NAMD-S Framework

The specific computational procedures involved in driving non-adiabatic molecular dynamics (NAMD) processes within the Hefei-NAMD-S framework will be discussed in detail step by step.

## Section S1.1: Database Structure

The database was constructed from 2000 step adiabatic molecular dynamics trajectories extracted from formamidinium lead iodide (FAPbI_3_) and its alkali metal doped systems. The database was partitioned using an equal-interval interpolation method, and the sampling rule for the train set (D_Train_) was defined as follows:

|  | $D_{Train}=4n-i, n\in\left[ 1, 500 \right], i\in[1, 4]$ | (1) |
| --- | --- | --- |

Here, *i* represent any fixed value within the interval [1, 4]. Based on equation (1), 500 D_Train_ data points were obtained, and the remaining 1500 data points from the adiabatic molecular dynamics trajectories were assigned to the test set (D_Test_). Based on this sampling strategy, the training configurations are distributed across the entire dynamical trajectory rather than being concentrated within a continuous segment, allowing the model to more comprehensively capture the structural fluctuations throughout the full trajectory. Four types of feature descriptors were extracted using the feature extraction functions within the Hefei-NAMD-S framework, namely atomic coordinates, atomic forces, bond lengths, and energy. To more accurately simulate the non-radiative recombination process, it was necessary to select the most representative properties as the target properties for machine learning (ML). Non-adiabatic coupling (NAC) and pure-dephasing time together determine the non-radiative recombination process. Specifically, NAC depends on the bandgap, electron-phonon coupling strength, and nuclear velocity, whereas the pure-dephasing time is governed by the amplitude of fluctuations in the bandgap relative to its mean value. Taking these factors into account, excited-state properties (valence band maximum, VBM; conduction band minimum, CBM; and the absolute value of the NAC factor, |NAC|) were selected as the target properties. VBM and CBM represent the band structure of the system, critically influencing the distribution and transitions of excited-state carriers, while |NAC| quantifies the coupling strength between electronic states, serving as a key parameter in describing non-radiative recombination processes. The goal was to predict the target properties of D_Test_ using the stacked model, thereby completing the excited-state properties along the entire adiabatic molecular dynamics trajectories and substantially reducing the computational resources and time costs required during the molecular dynamics process. In addition, it should be emphasized that the machine learning predictions are not performed in a recursive manner. Instead, each prediction is independent, and the prediction of each target property at a given time step does not rely on the result from the previous time step. Therefore, no progressive error accumulation occurs during the prediction of long-time dynamical trajectories.

## Section S1.2: Feature Descriptors and Target Properties

The database consists of four types of feature descriptors: atomic coordinates, atomic forces, bond lengths, and energy, and three target properties: VBM, CBM, and |NAC|. The explanations for the ML inputs are as follows:

| Atomic Coordinates | x_i_ | The atomic coordinate of the atom labeled i along the x-direction. |
| --- | --- | --- |
|  | y_i_ | The atomic coordinate of the atom labeled i along the y-direction. |
|  | z_i_ | The atomic coordinate of the atom labeled i along the z-direction. |
| Atomic Forces | Fx_i_ | The atomic force of the atom labeled i along the x-direction. |
|  | Fy_i_ | The atomic force of the atom labeled i along the y-direction. |
|  | Fz_i_ | The atomic force of the atom labeled i along the z-direction. |
| Bond Lengths | A_i_-A_j_ | The bond length between atoms labeled i and j. |
| Energy | Energy | System energy |
|  | Fermi | System Fermi level |
| Target Properties | VBM | Valence band maximum |
|  | CBM | Conduction band minimum |
|  | \|NAC\| | The absolute value of the non-adiabatic coupling factor |

## Section S1.3: Feature Engineering

In view of the importance of feature engineering, we conducted feature collection, cleaning, selection, and scaling in sequence. As one of the most widely used feature selection methods, Pearson correlation ranks and selects descriptors by quantifying their correlations with the target properties. Without relying on a specific machine learning model, this approach can efficiently remove redundant features, reduce data dimensionality, and improve the stability of model training. This feature selection strategy is particularly important for our work because it directly links feature descriptors with target properties, thereby helping to establish the relationships between structural features and the corresponding electronic and non-adiabatic properties. Another commonly used feature extraction method is principal component analysis (PCA), which uses linear transformations to convert the original features into a set of linearly uncorrelated features and thereby helps identify the dominant features influencing the target properties. However, because PCA is based on linear projections, it may be less effective for datasets involving nonlinear relationships. In addition, feature importance methods based on machine learning models, such as random forest feature importance, are also widely used. Although these methods can capture nonlinear relationships, their feature selection results depend on the model training process and may be affected by the data distribution and feature types. Therefore, after considering computational efficiency, interpretability, and stability, we selected Pearson correlation as the feature selection method in this work.

We compared the predictive performance of the model when the top 10, top 20, and top 30 descriptors were selected, as shown in **Table S1-S3**. The results show that the model achieves good predictive performance for both VBM and CBM on the D_Train_ and D_Test_. The most pronounced difference in predictive performance is observed for |NAC|. For example, in the Cs_i_ system, the coefficient of determination (R^2^) value increases from approximately 0.60 when using the top 10 or top 30 descriptors to 0.85 when using the top 20 descriptors. In addition, although the predictive performance obtained using the top 30 descriptors is slightly better than, or nearly comparable to, that obtained using the top 20 descriptors in a few cases, selecting 30 descriptors inevitably increases the computational cost while providing only marginal improvement in prediction accuracy. Together with the evaluation based on root means square error (RMSE) and mean absolute error (MAE), these results strongly support the reliability and rationality of selecting the top 20 descriptors for machine learning prediction. As shown in **Figures S1-S21**, Pearson correlation analysis was employed to alleviate the curse of dimensionality, and the top 20 ranked feature descriptors were selected as the inputs for the subsequent regression models. Additionally, to eliminate the influence of differences in scales among features on model performance, all data were normalized.

Table S1. Coefficient of determination (R^2^) prediction evaluation results for the three target properties in the pristine and doped systems using different numbers of features selected by Pearson correlation.

| **R^2^** |  | **Train set** | | | **Test set** | | |
| --- | --- | --- | --- | --- | --- | --- | --- |
|  |  | **Top 10** | **Top 20** | **Top 30** | **Top 10** | **Top 20** | **Top 30** |
| **Pristine** | **VBM** | 0.97 | 0.98 | 0.98 | 0.98 | 0.99 | 0.99 |
|  | **CBM** | 0.97 | 0.97 | 0.98 | 0.98 | 0.98 | 0.99 |
|  | **\|NAC\|** | 0.90 | 0.90 | 0.91 | 0.89 | 0.94 | 0.95 |
| **FAK** | **VBM** | 0.99 | 0.99 | 0.98 | 0.99 | 0.99 | 0.99 |
|  | **CBM** | 0.94 | 0.95 | 0.96 | 0.98 | 0.98 | 0.98 |
|  | **\|NAC\|** | 0.69 | 0.70 | 0.72 | 0.83 | 0.85 | 0.85 |
| **FARb** | **VBM** | 0.99 | 0.98 | 0.98 | 0.99 | 0.99 | 0.99 |
|  | **CBM** | 0.95 | 0.97 | 0.98 | 0.97 | 0.99 | 0.99 |
|  | **\|NAC\|** | 0.83 | 0.81 | 0.83 | 0.92 | 0.92 | 0.91 |
| **FACs** | **VBM** | 0.97 | 0.99 | 0.98 | 0.99 | 0.99 | 0.99 |
|  | **CBM** | 0.98 | 0.98 | 0.98 | 0.99 | 0.99 | 0.99 |
|  | **\|NAC\|** | 0.77 | 0.78 | 0.79 | 0.87 | 0.86 | 0.88 |
| **Ki** | **VBM** | 0.99 | 0.98 | 0.98 |  |  |  |
|  | **CBM** | 0.98 | 0.98 | 0.98 |  |  |  |
|  | **\|NAC\|** | 0.79 | 0.80 | 0.79 |  |  |  |
| **Rbi** | **VBM** | 0.99 | 0.99 | 0.99 |  |  |  |
|  | **CBM** | 0.99 | 0.99 | 0.99 |  |  |  |
|  | **\|NAC\|** | 0.88 | 0.90 | 0.89 |  |  |  |
| **Csi** | **VBM** | 0.99 | 0.98 | 0.98 |  |  |  |
|  | **CBM** | 0.98 | 0.99 | 0.99 |  |  |  |
|  | **\|NAC\|** | 0.61 | 0.85 | 0.62 |  |  |  |

Table S2. Root means square error (RMSE) prediction evaluation results for the three target properties in the pristine and doped systems using different numbers of features selected by Pearson correlation.

| **RMSE** |  | **Train set** | | | **Test set** | | |
| --- | --- | --- | --- | --- | --- | --- | --- |
|  |  | **Top 10** | **Top 20** | **Top 30** | **Top 10** | **Top 20** | **Top 30** |
| **Pristine** | **VBM** | 0.04 | 0.03 | 0.03 | 0.02 | 0.02 | 0.02 |
|  | **CBM** | 0.03 | 0.04 | 0.03 | 0.02 | 0.03 | 0.02 |
|  | **\|NAC\|** | 0.06 | 0.07 | 0.06 | 0.07 | 0.06 | 0.05 |
| **FAK** | **VBM** | 0.02 | 0.03 | 0.03 | 0.01 | 0.02 | 0.02 |
|  | **CBM** | 0.04 | 0.04 | 0.04 | 0.02 | 0.03 | 0.03 |
|  | **\|NAC\|** | 0.12 | 0.12 | 0.12 | 0.09 | 0.09 | 0.09 |
| **FARb** | **VBM** | 0.03 | 0.03 | 0.03 | 0.02 | 0.02 | 0.02 |
|  | **CBM** | 0.05 | 0.04 | 0.03 | 0.03 | 0.02 | 0.02 |
|  | **\|NAC\|** | 0.09 | 0.10 | 0.09 | 0.07 | 0.06 | 0.07 |
| **FACs** | **VBM** | 0.05 | 0.05 | 0.04 | 0.02 | 0.03 | 0.02 |
|  | **CBM** | 0.03 | 0.03 | 0.03 | 0.02 | 0.02 | 0.02 |
|  | **\|NAC\|** | 0.10 | 0.10 | 0.10 | 0.08 | 0.08 | 0.08 |
| **Ki** | **VBM** | 0.03 | 0.03 | 0.02 |  |  |  |
|  | **CBM** | 0.03 | 0.02 | 0.02 |  |  |  |
|  | **\|NAC\|** | 0.11 | 0.10 | 0.11 |  |  |  |
| **Rbi** | **VBM** | 0.02 | 0.02 | 0.02 |  |  |  |
|  | **CBM** | 0.02 | 0.03 | 0.02 |  |  |  |
|  | **\|NAC\|** | 0.08 | 0.07 | 0.07 |  |  |  |
| **Csi** | **VBM** | 0.03 | 0.03 | 0.03 |  |  |  |
|  | **CBM** | 0.03 | 0.03 | 0.02 |  |  |  |
|  | **\|NAC\|** | 0.13 | 0.07 | 0.13 |  |  |  |

Table S3. Mean absolute error (MAE) prediction evaluation results for the three target properties in the pristine and doped systems using different numbers of features selected by Pearson correlation.

| **MAE** |  | **Train set** | | | **Test set** | | |
| --- | --- | --- | --- | --- | --- | --- | --- |
|  |  | **Top 10** | **Top 20** | **Top 30** | **Top 10** | **Top 20** | **Top 30** |
| **Pristine** | **VBM** | 0.03 | 0.03 | 0.02 | 0.02 | 0.01 | 0.01 |
|  | **CBM** | 0.03 | 0.03 | 0.02 | 0.02 | 0.02 | 0.02 |
|  | **\|NAC\|** | 0.05 | 0.05 | 0.05 | 0.05 | 0.04 | 0.04 |
| **FAK** | **VBM** | 0.02 | 0.02 | 0.03 | 0.01 | 0.02 | 0.02 |
|  | **CBM** | 0.03 | 0.03 | 0.03 | 0.02 | 0.02 | 0.02 |
|  | **\|NAC\|** | 0.09 | 0.09 | 0.09 | 0.07 | 0.07 | 0.07 |
| **FARb** | **VBM** | 0.02 | 0.02 | 0.02 | 0.02 | 0.01 | 0.01 |
|  | **CBM** | 0.03 | 0.03 | 0.02 | 0.02 | 0.02 | 0.01 |
|  | **\|NAC\|** | 0.07 | 0.08 | 0.07 | 0.05 | 0.05 | 0.05 |
| **FACs** | **VBM** | 0.03 | 0.03 | 0.03 | 0.02 | 0.02 | 0.02 |
|  | **CBM** | 0.02 | 0.02 | 0.02 | 0.01 | 0.02 | 0.02 |
|  | **\|NAC\|** | 0.08 | 0.08 | 0.08 | 0.06 | 0.06 | 0.06 |
| **Ki** | **VBM** | 0.02 | 0.02 | 0.02 |  |  |  |
|  | **CBM** | 0.02 | 0.02 | 0.02 |  |  |  |
|  | **\|NAC\|** | 0.08 | 0.08 | 0.08 |  |  |  |
| **Rbi** | **VBM** | 0.01 | 0.01 | 0.01 |  |  |  |
|  | **CBM** | 0.02 | 0.02 | 0.02 |  |  |  |
|  | **\|NAC\|** | 0.06 | 0.06 | 0.06 |  |  |  |
| **Csi** | **VBM** | 0.02 | 0.02 | 0.02 |  |  |  |
|  | **CBM** | 0.02 | 0.02 | 0.02 |  |  |  |
|  | **\|NAC\|** | 0.10 | 0.06 | 0.10 |  |  |  |


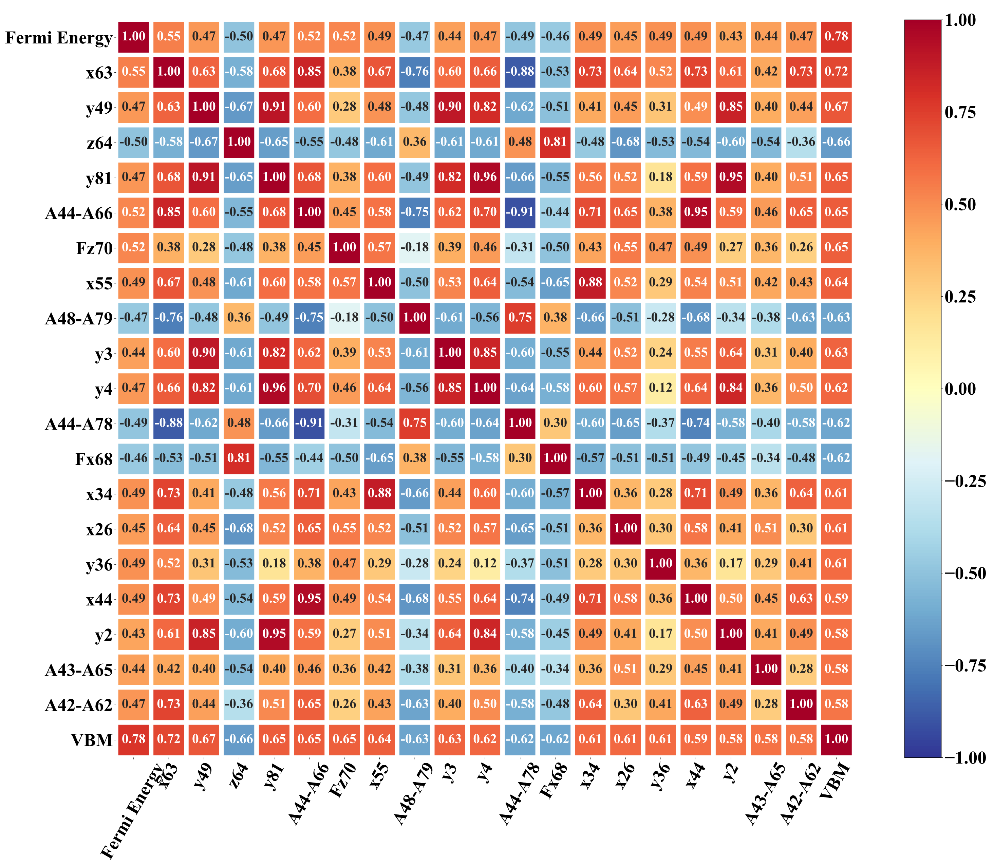


Figure S1. Heatmap of the correlation between the VBM and the top 20 feature descriptors ranked by Pearson correlation coefficient in the pristine system.


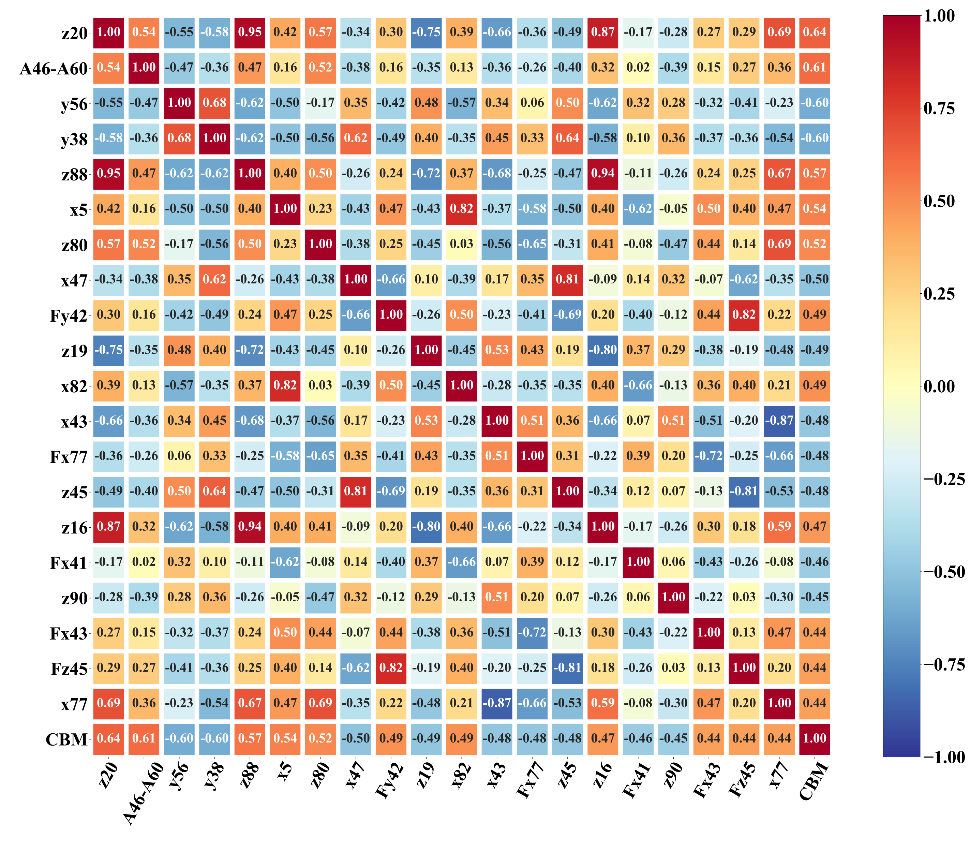


Figure S2. Heatmap of the correlation between the CBM and the top 20 feature descriptors ranked by Pearson correlation coefficient in the pristine system.


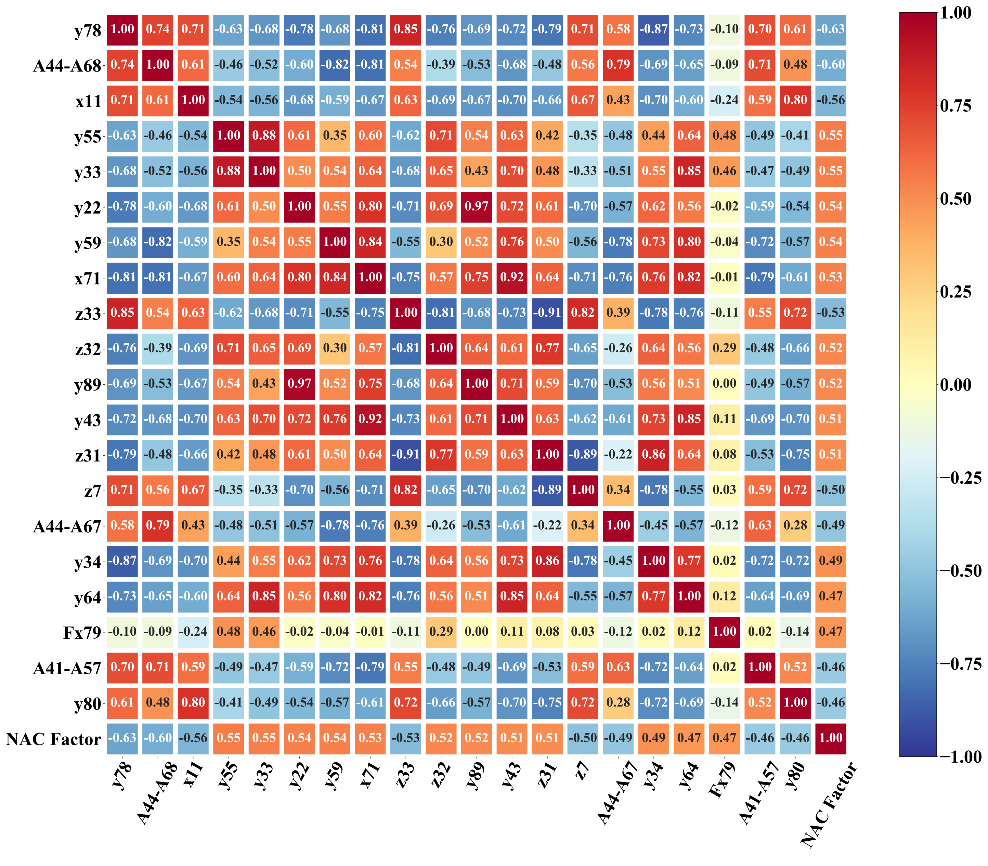


Figure S3. Heatmap of the correlation between the |NAC| and the top 20 feature descriptors ranked by Pearson correlation coefficient in the pristine system.


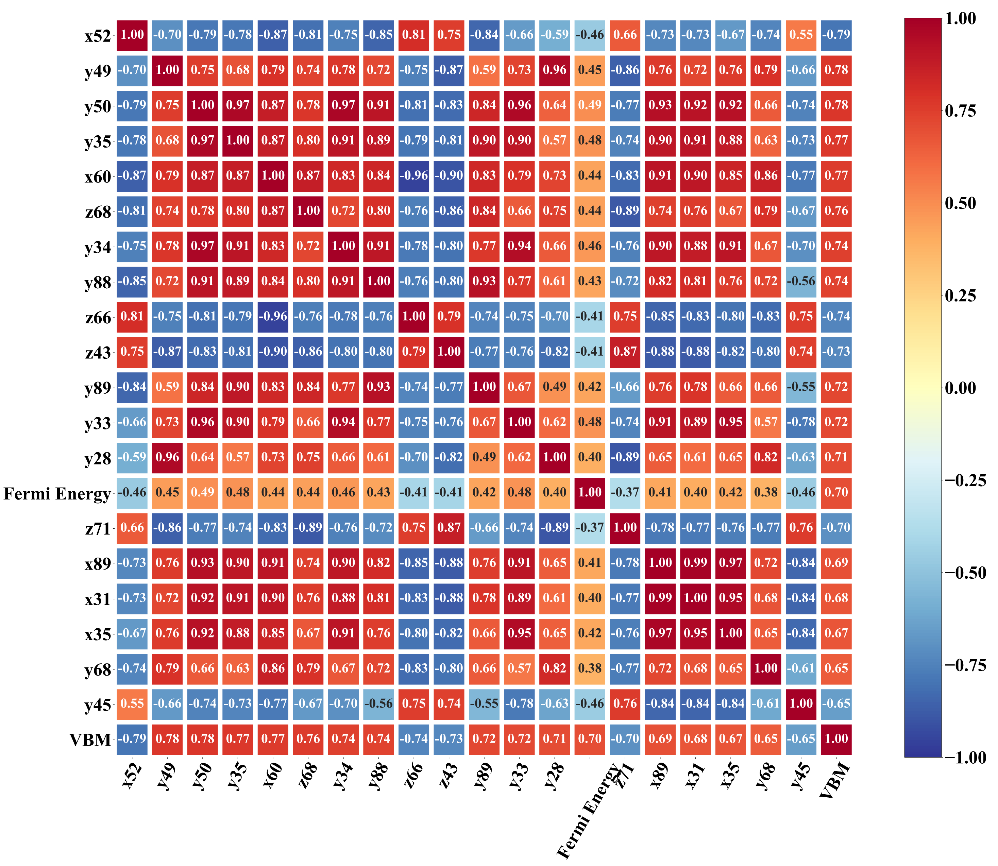


Figure S4. Heatmap of the correlation between the VBM and the top 20 feature descriptors ranked by Pearson correlation coefficient in the FA_K_ system.


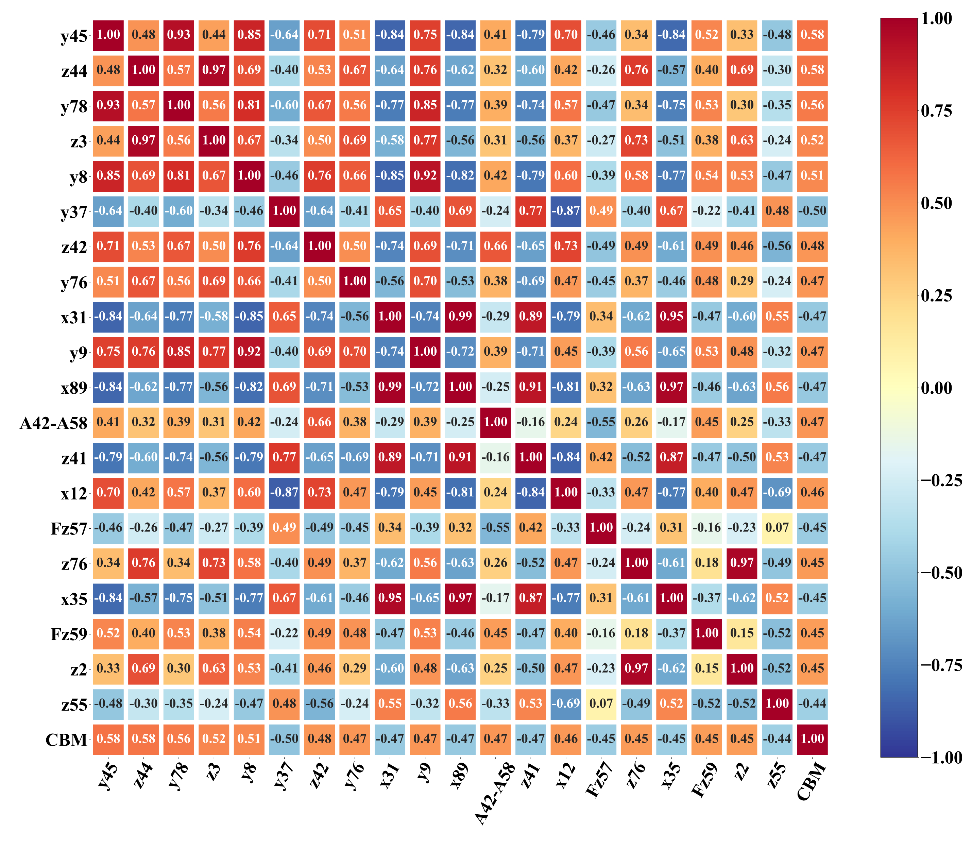


Figure S5. Heatmap of the correlation between the CBM and the top 20 feature descriptors ranked by Pearson correlation coefficient in the FA_K_ system.


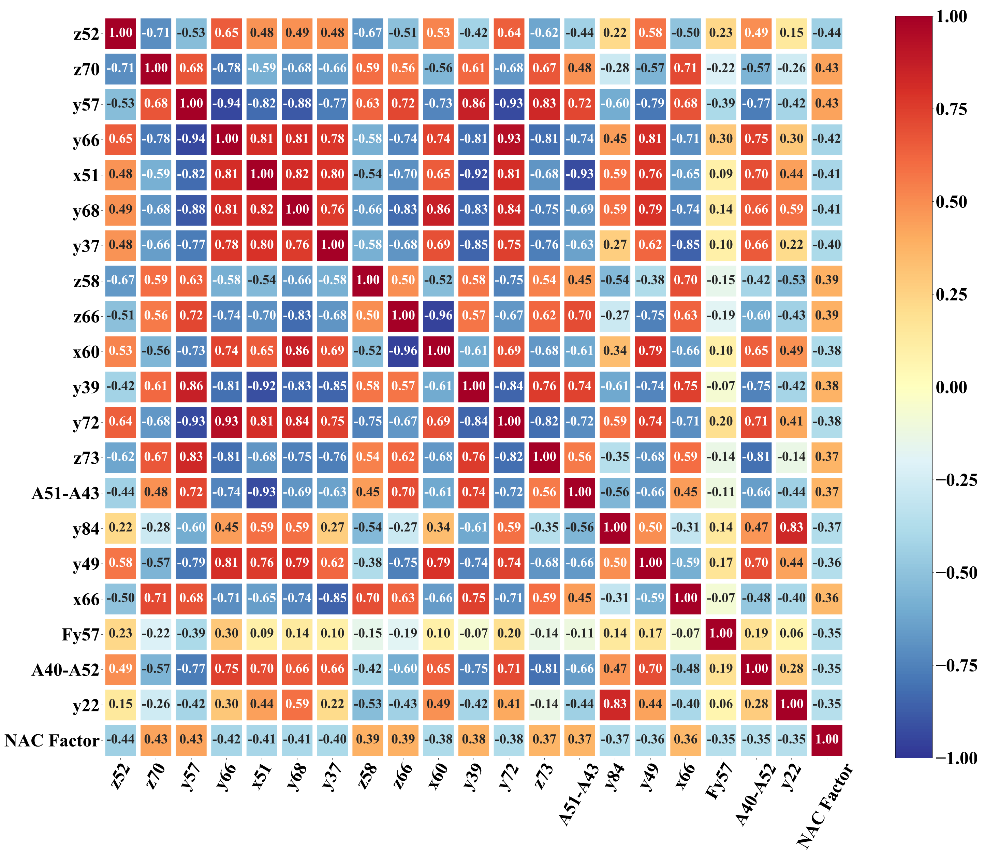


Figure S6. Heatmap of the correlation between the |NAC| and the top 20 feature descriptors ranked by Pearson correlation coefficient in the FA_K_ system.


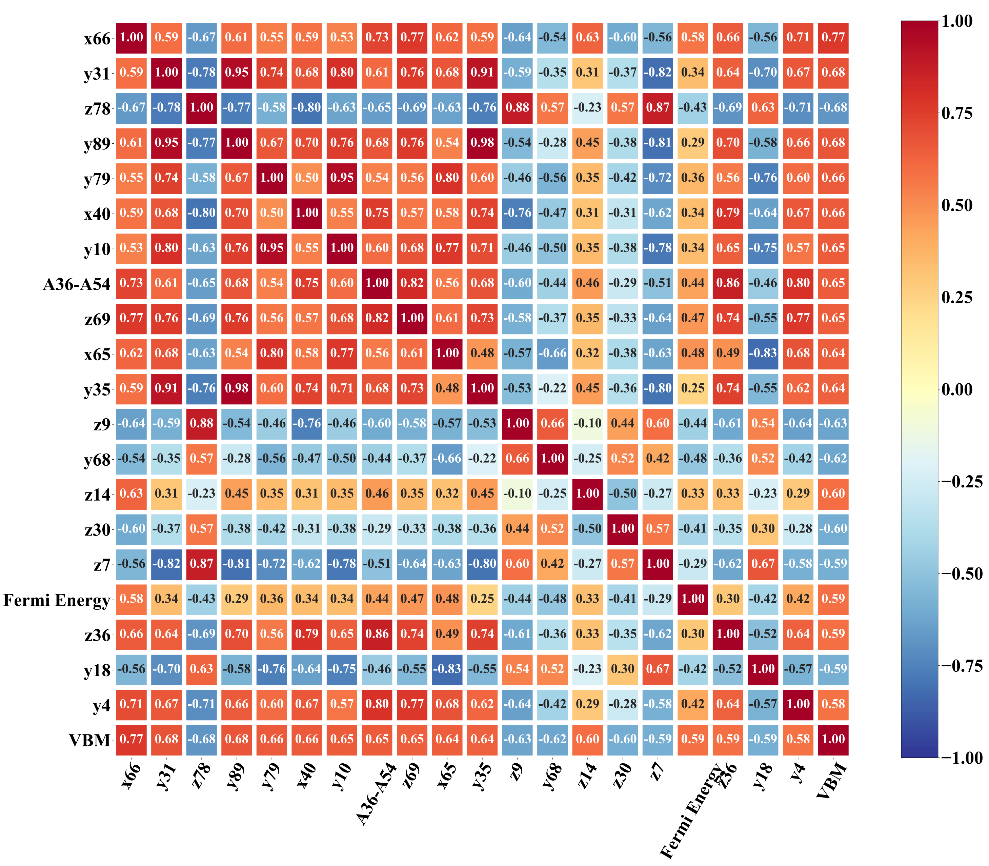


Figure S7. Heatmap of the correlation between the VBM and the top 20 feature descriptors ranked by Pearson correlation coefficient in the FA_Rb_ system.


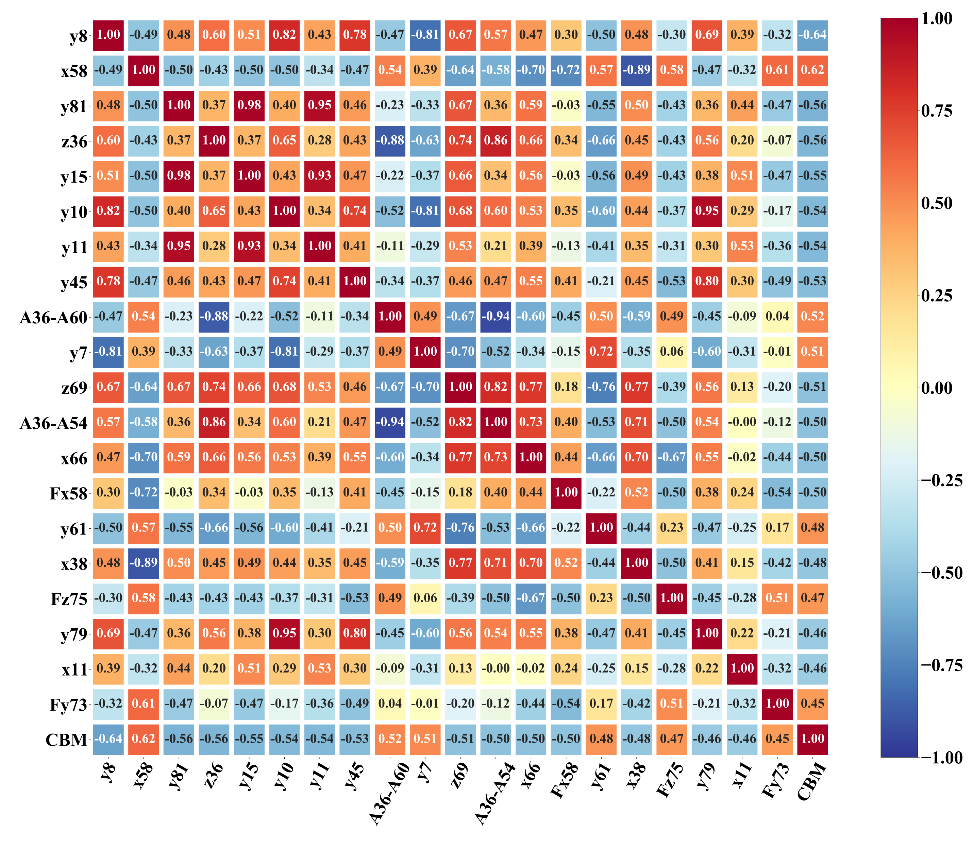


Figure S8. Heatmap of the correlation between the CBM and the top 20 feature descriptors ranked by Pearson correlation coefficient in the FA_Rb_ system.


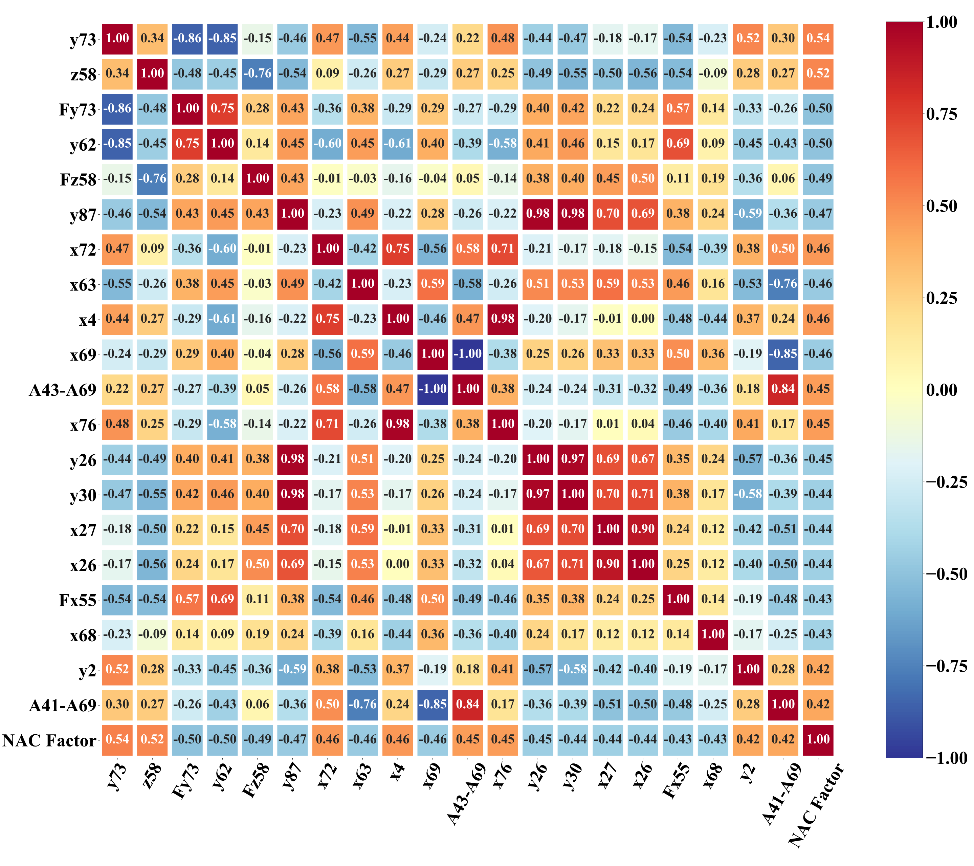


Figure S9. Heatmap of the correlation between the |NAC| and the top 20 feature descriptors ranked by Pearson correlation coefficient in the FA_Rb_ system.


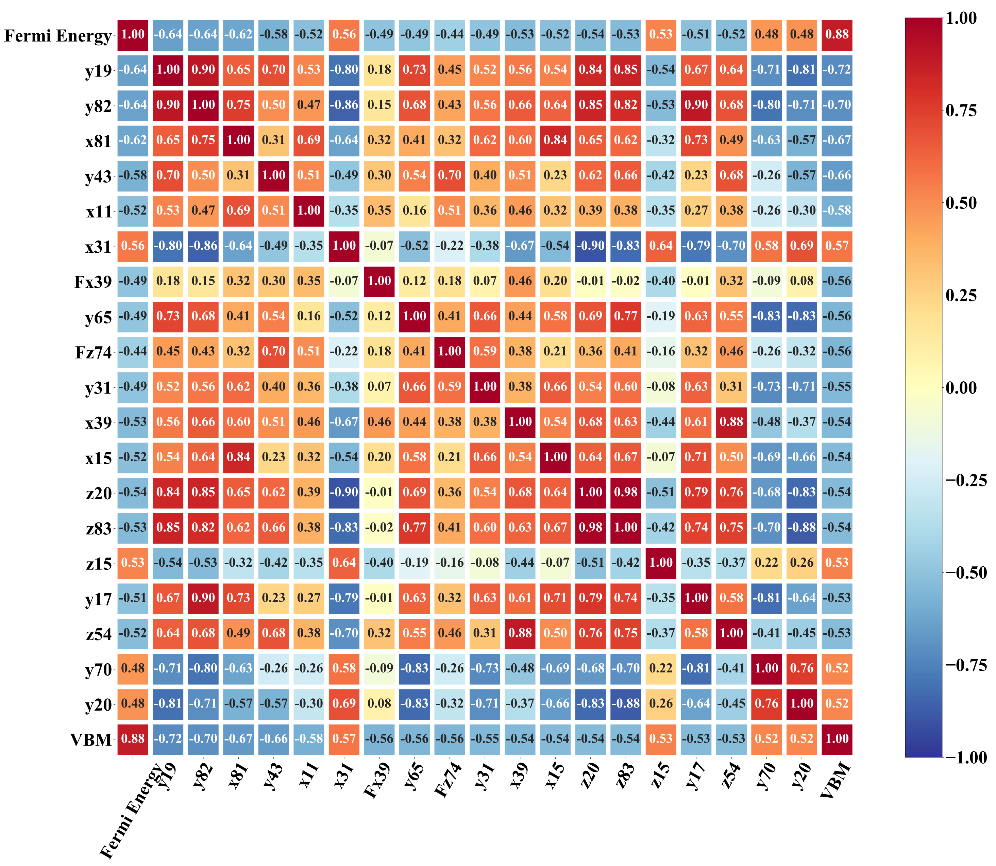


Figure S10. Heatmap of the correlation between the VBM and the top 20 feature descriptors ranked by Pearson correlation coefficient in the FA_Cs_ system.


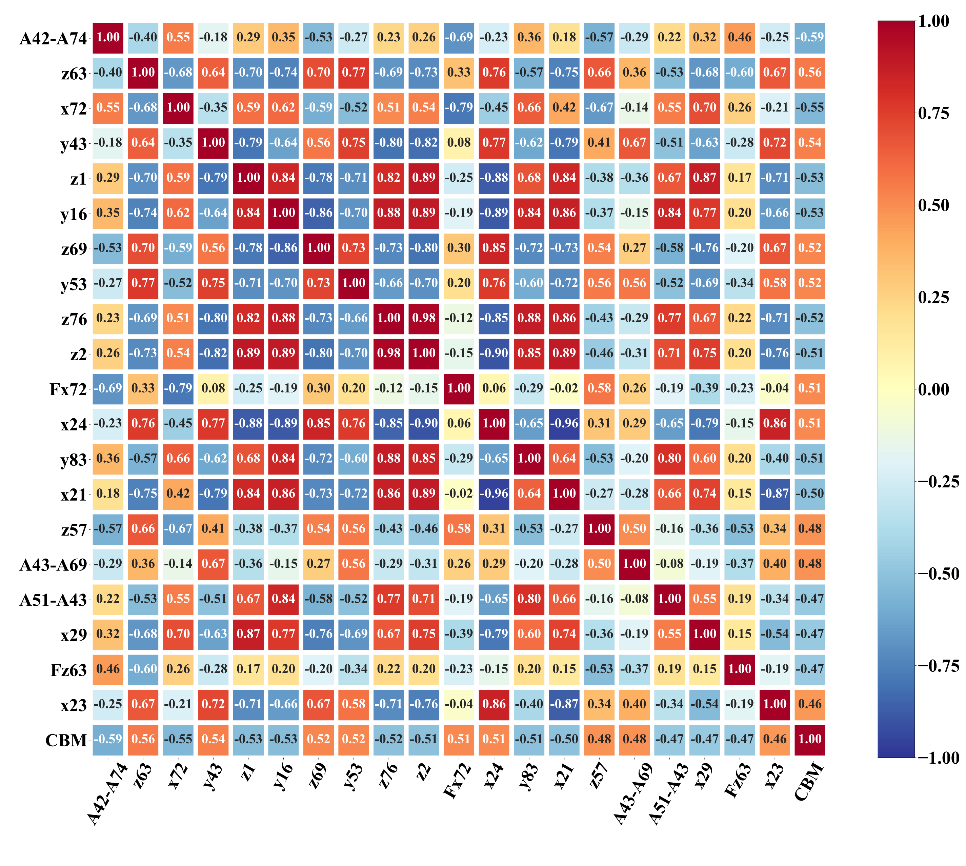


Figure S11. Heatmap of the correlation between the CBM and the top 20 feature descriptors ranked by Pearson correlation coefficient in the FA_Cs_ system.


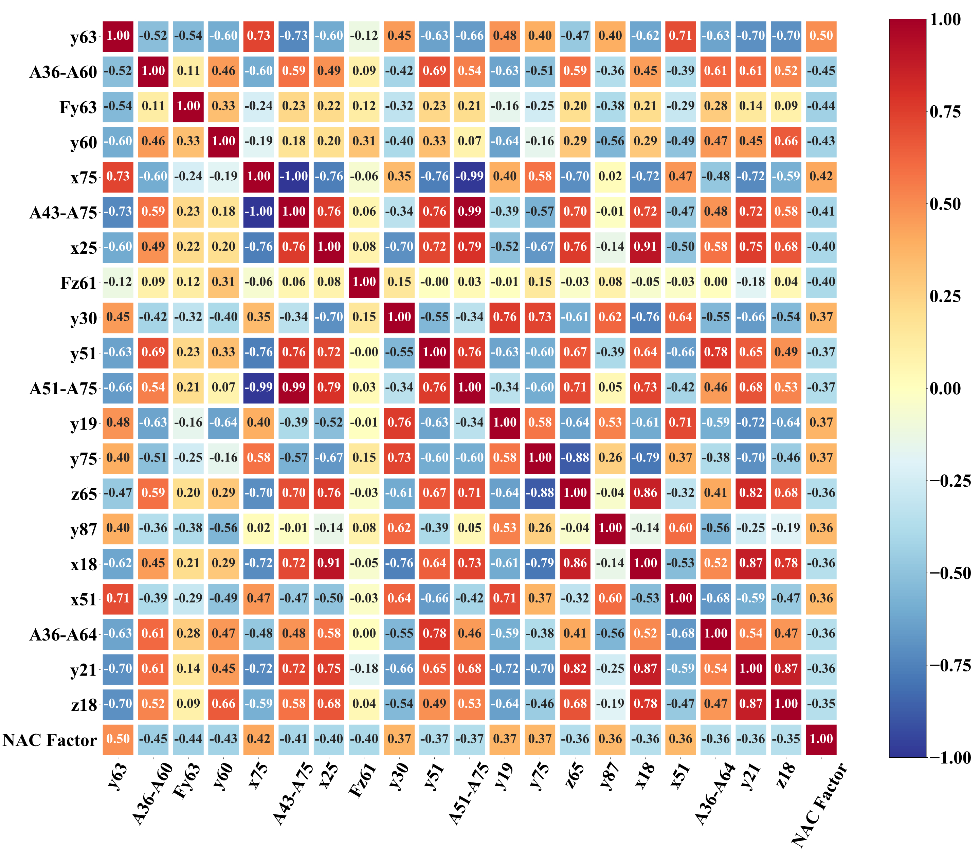


Figure S12. Heatmap of the correlation between the |NAC| and the top 20 feature descriptors ranked by Pearson correlation coefficient in the FA_Cs_ system.


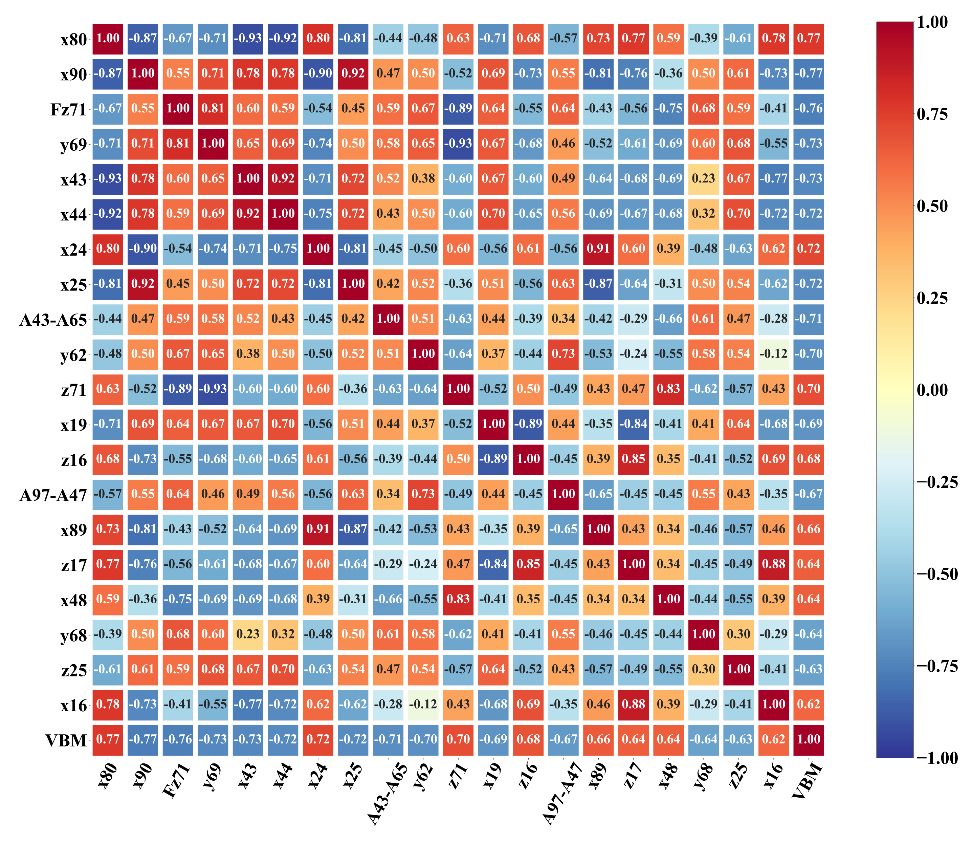


Figure S13. Heatmap of the correlation between the VBM and the top 20 feature descriptors ranked by Pearson correlation coefficient in the K_i_ system.


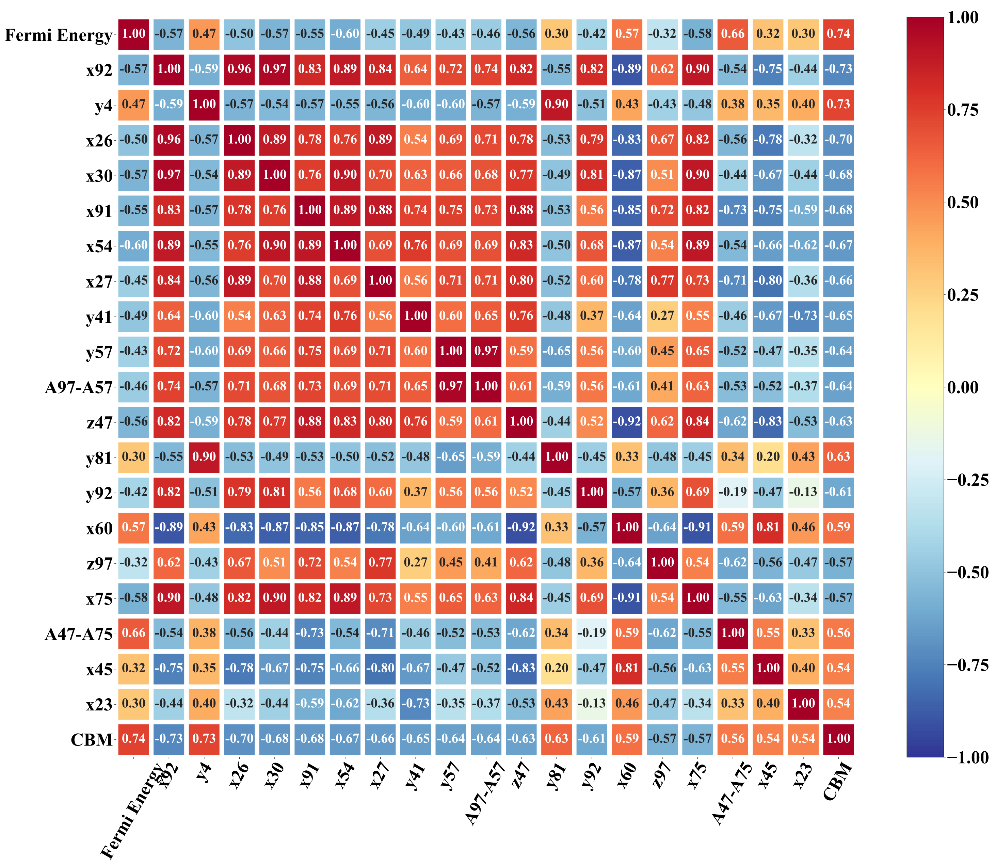


Figure S14. Heatmap of the correlation between the CBM and the top 20 feature descriptors ranked by Pearson correlation coefficient in the K_i_ system.


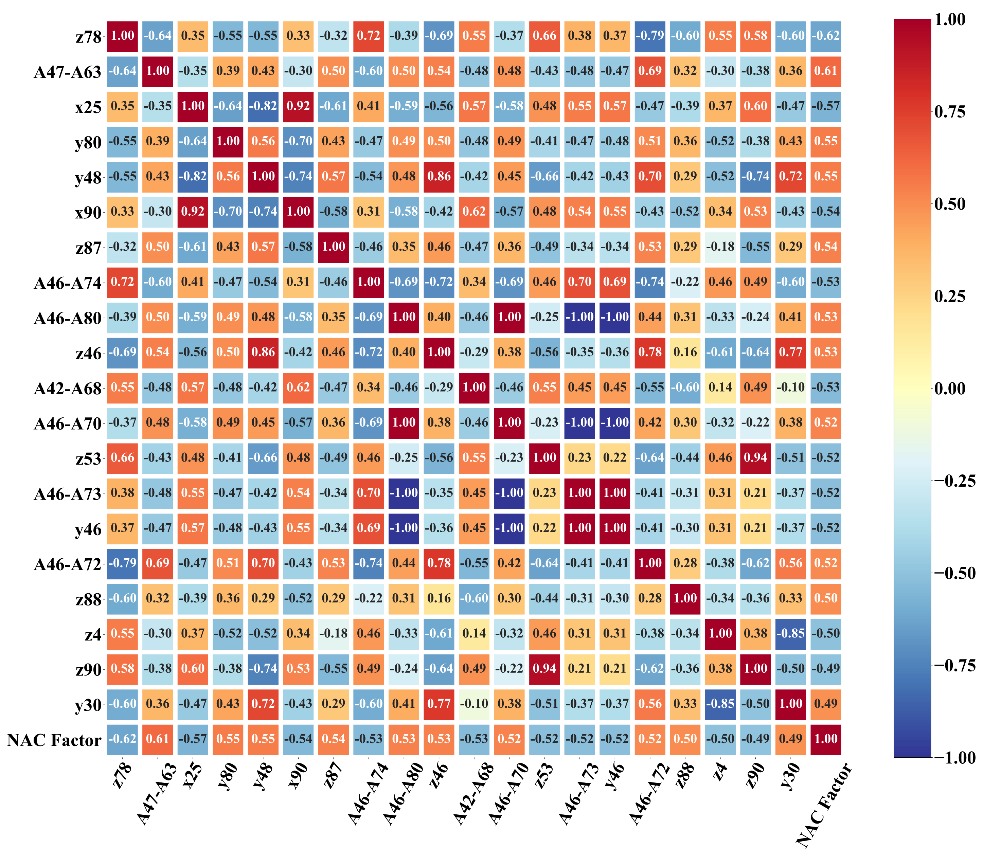


Figure S15. Heatmap of the correlation between the |NAC| and the top 20 feature descriptors ranked by Pearson correlation coefficient in the K_i_ system.


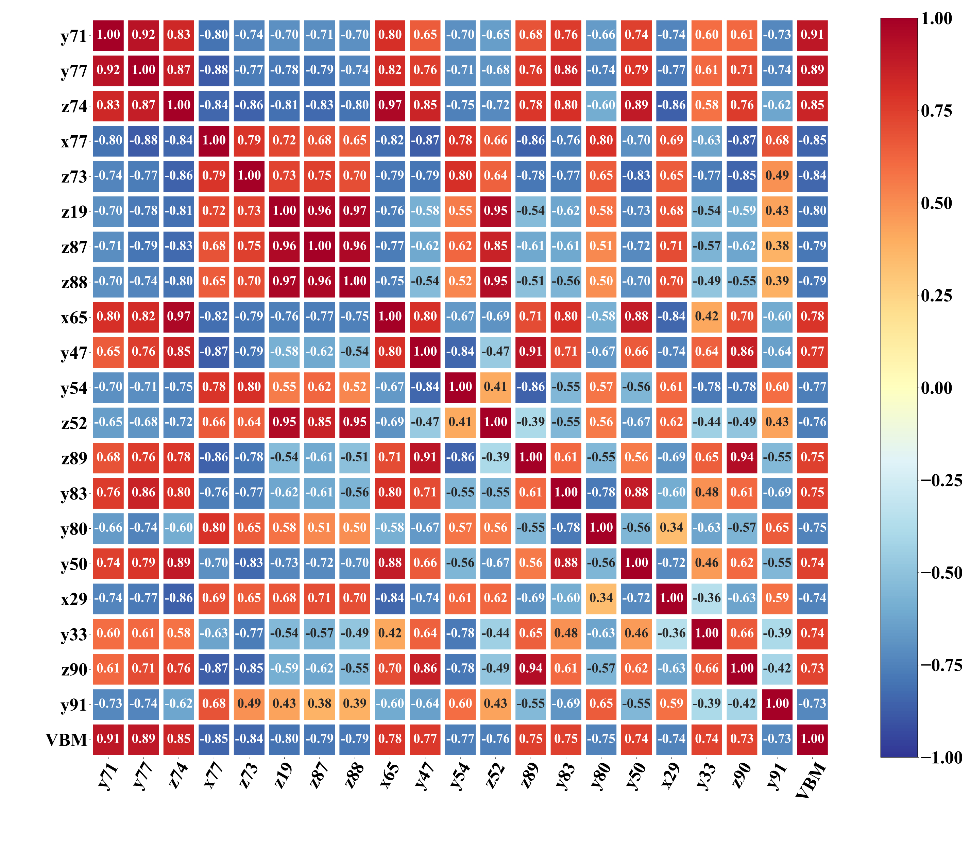


Figure S16. Heatmap of the correlation between the VBM and the top 20 feature descriptors ranked by Pearson correlation coefficient in the Rb_i_ system.


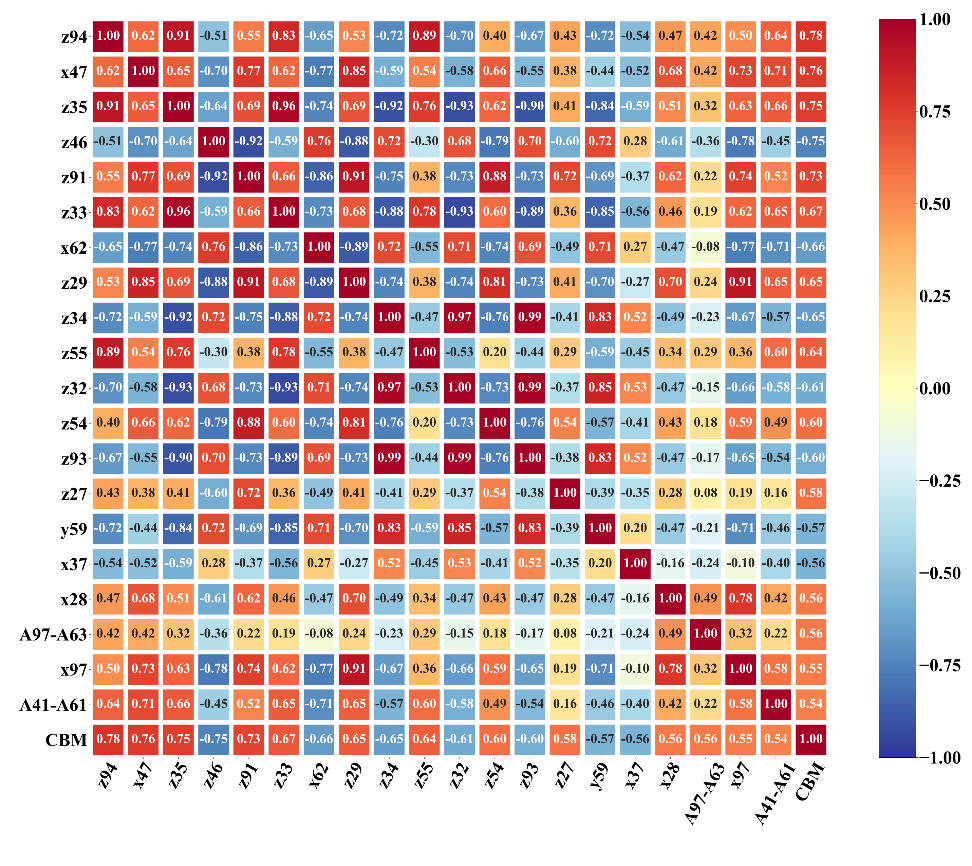


Figure S17. Heatmap of the correlation between the CBM and the top 20 feature descriptors ranked by Pearson correlation coefficient in the Rb_i_ system.


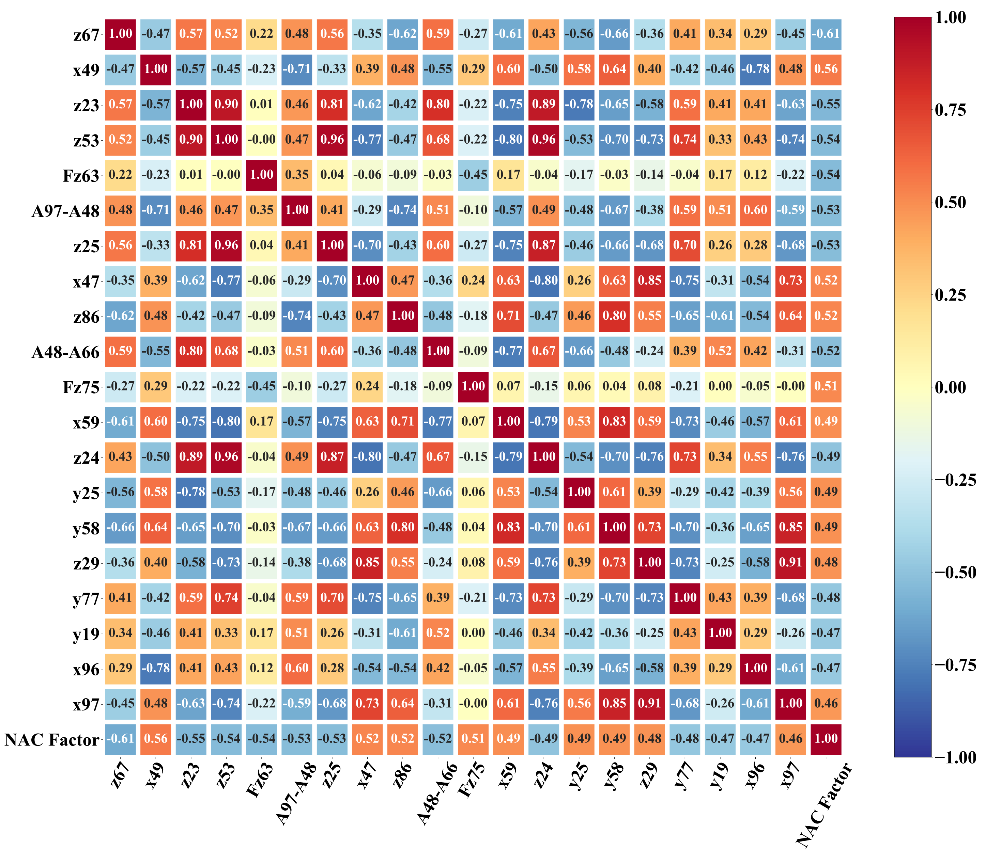


Figure S18. Heatmap of the correlation between the |NAC| and the top 20 feature descriptors ranked by Pearson correlation coefficient in the Rb_i_ system.


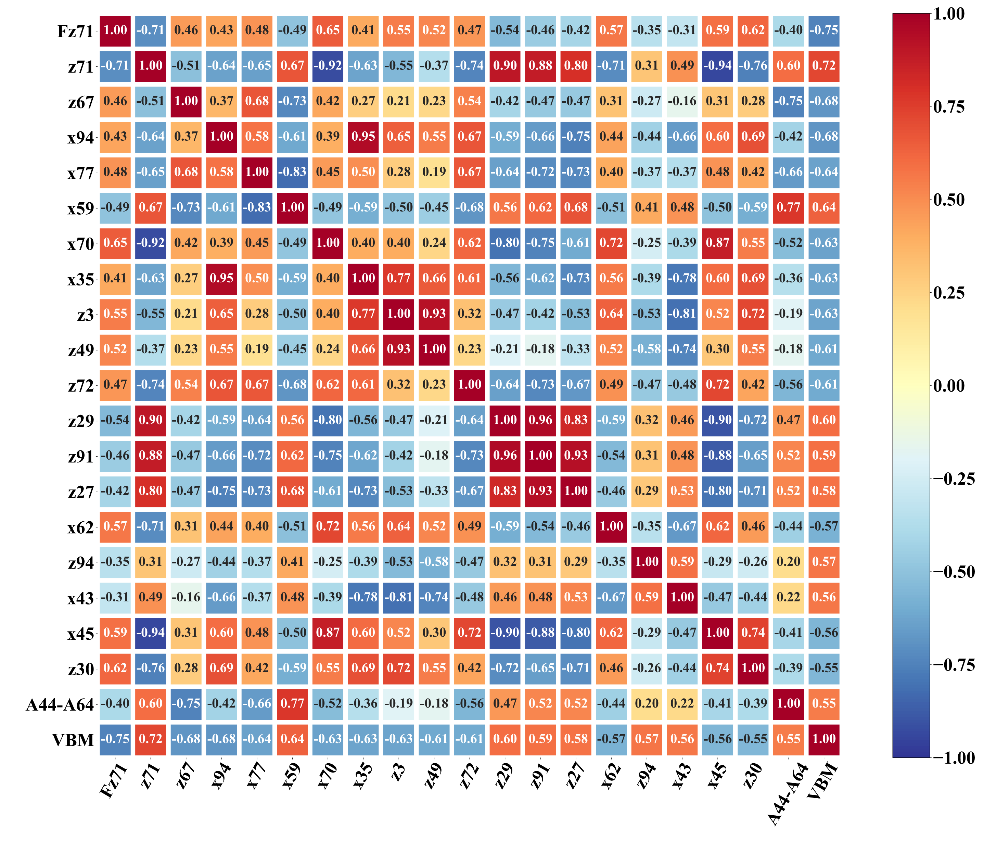


Figure S19. Heatmap of the correlation between the VBM and the top 20 feature descriptors ranked by Pearson correlation coefficient in the Cs_i_ system.


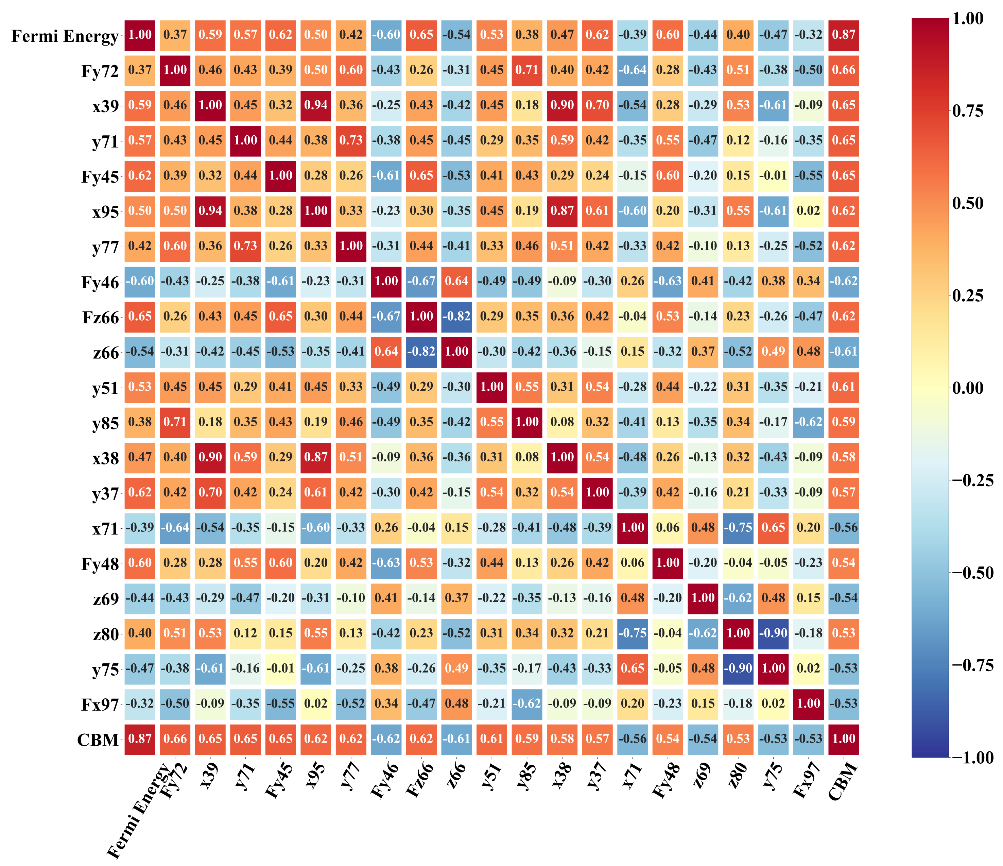


Figure S20. Heatmap of the correlation between the CBM and the top 20 feature descriptors ranked by Pearson correlation coefficient in the Cs_i_ system.


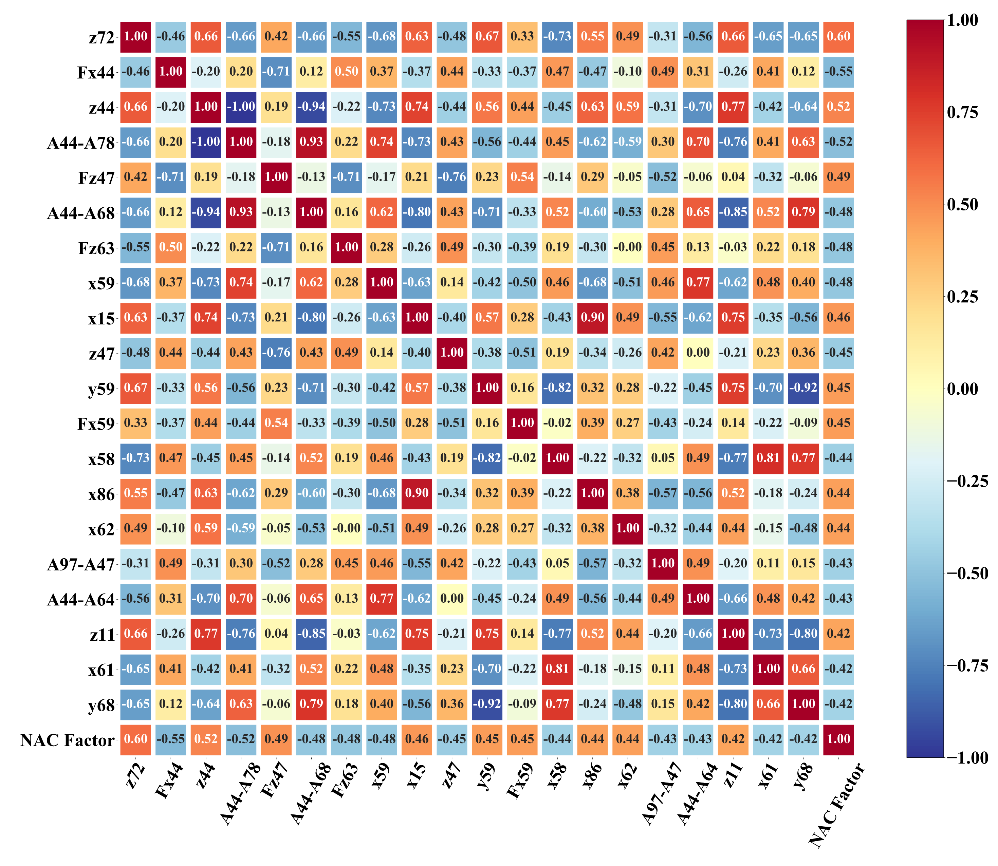


Figure S21. Heatmap of the correlation between the |NAC| and the top 20 feature descriptors ranked by Pearson correlation coefficient in the Cs_i_ system.

## Section S1.4: Hefei-NAMD-S Framework

At the core of the Hefei-NAMD-S framework lies the construction of the stacked model. RF^[1-2]^ and multilayer perceptron (MLP)^[3-4]^ algorithms were selected as base models, while RF was also employed as the meta model. The model architecture is illustrated in **Figure 1b**. RF, a representative ensemble algorithm based on decision trees, excels at capturing nonlinear relationships in the dataset but exhibits certain limitations when addressing complex feature interactions. In contrast, MLP, as a neural network algorithm, can autonomously learn deep representations from dataset, although it tends to be more sensitive to hyperparameter configurations. By integrating these two algorithms with distinct learning mechanisms, their complementary advantages can be fully utilized to uncover intrinsic correlations within the dataset, thereby substantially improving the predictive performance of the stacked model. The fundamental principle of the stacked model is that the meta model conducts a secondary prediction based on the outputs of the base models to generate the final result.

Next, the working principle of the stacked model is introduced. First, the RF and MLP base models are trained, hyperparameter optimization (**Tables S4-S6**), and validated via cross-validation using the train set, with each model generating its own prediction results. Subsequently, the prediction results from the base models are used as new feature inputs for the RF meta model, which is then subjected to training, hyperparameter optimization, and cross-validation to further learn the relationship between the base model predictions and the calculated values, ultimately producing the final prediction results. To avoid information leakage during the stacking process, the training inputs for the meta model were constructed from out-of-fold predictions obtained through five-fold cross validation, rather than from direct predictions of the base models on the full D_Train_. This strategy also ensures the independence between the D_Train_ and D_Test_. After the training process, the test set is input into the stacked model configured with the optimal hyperparameters to predict the target properties. Overall, the stacked model generates the final predictions by performing a secondary prediction on the outputs of the base models through the meta model.

Table S4. Optimal hyperparameters for machine learning stacked model for VBM prediction, obtained via grid search.

| **VBM** | |  | **Pristine** | **FA_K_** | **FA_Rb_** | **FA_Cs_** | **K_i_** | **Rb_i_** | **Cs_i_** |
| --- | --- | --- | --- | --- | --- | --- | --- | --- | --- |
| **Stacking model** | **Base model_RF** | **n_estimators** | 50 | 100 | 150 | 150 | 50 | 100 | 150 |
|  |  | **max_features** | 'sqrt' | 'sqrt' | 'sqrt' | 'auto' | 'sqrt' | 'sqrt' | 'sqrt' |
|  |  | **max_depth** | 10 | 30 | 10 | 20 | 10 | 30 | 10 |
|  |  | **min_samples_split** | 3 | 2 | 4 | 2 | 3 | 3 | 5 |
|  |  | **min_samples_leaf** | 1 | 1 | 1 | 3 | 1 | 1 | 1 |
|  | **Base model_MLP** | **hidden_layer_sizes** | (100, 100) | (150, 150) | (100, 100) | (100, 100) | (150, 150) | (50, 50) | (150, 150) |
|  |  | **max_iter** | 400 | 400 | 600 | 400 | 400 | 400 | 400 |
|  |  | **activation** | 'relu' | 'relu' | 'tanh' | 'tanh' | 'relu' | 'tanh' | 'tanh' |
|  |  | **solver** | 'adam' | 'adam' | 'sgd' | 'adam' | 'adam' | 'sgd' | 'sgd' |
|  |  | **alpha** | 0.0001 | 0.1 | 0.1 | 0.0001 | 0.001 | 0.0001 | 0.01 |
|  |  | **learning_rate** | 'constant' | 'constant' | 'constant' | 'constant' | 'constant' | 'adaptive' | 'constant' |
|  | **Meta model_RF** | **n_estimators** | 50 | 100 | 150 | 150 | 50 | 100 | 150 |
|  |  | **max_features** | 'sqrt' | 'sqrt' | 'sqrt' | 'auto' | 'sqrt' | 'sqrt' | 'sqrt' |
|  |  | **max_depth** | 10 | 30 | 10 | 20 | 10 | 30 | 10 |
|  |  | **min_samples_split** | 3 | 2 | 4 | 2 | 3 | 3 | 5 |
|  |  | **min_samples_leaf** | 1 | 1 | 1 | 3 | 1 | 1 | 1 |

Table S5. Optimal hyperparameters for machine learning stacked model for CBM prediction, obtained via grid search.

| **CBM** | |  | **Pristine** | **FA_K_** | **FA_Rb_** | **FA_Cs_** | **K_i_** | **Rb_i_** | **Cs_i_** |
| --- | --- | --- | --- | --- | --- | --- | --- | --- | --- |
| **Stacking model** | **Base model_RF** | **n_estimators** | 100 | 50 | 50 | 50 | 150 | 50 | 200 |
|  |  | **max_features** | 'sqrt' | 'auto' | 'sqrt' | 'sqrt' | 'sqrt' | 'auto' | 'auto' |
|  |  | **max_depth** | 20 | 10 | 10 | 20 | 20 | 10 | 20 |
|  |  | **min_samples_split** | 2 | 4 | 5 | 2 | 2 | 2 | 3 |
|  |  | **min_samples_leaf** | 3 | 1 | 2 | 2 | 2 | 1 | 1 |
|  | **Base model_MLP** | **hidden_layer_sizes** | (150, 150) | (50, 50) | (150, 150) | (150, 150) | (100, 100) | (100, 100) | (150, 150) |
|  |  | **max_iter** | 400 | 400 | 600 | 400 | 400 | 400 | 400 |
|  |  | **activation** | 'relu' | 'tanh' | 'tanh' | 'tanh' | 'relu' | 'tanh' | 'relu' |
|  |  | **solver** | 'sgd' | 'adam' | 'sgd' | 'sgd' | 'adam' | 'adam' | 'adam' |
|  |  | **alpha** | 0.1 | 0.1 | 0.1 | 0.0001 | 0.001 | 0.01 | 0.001 |
|  |  | **learning_rate** | 'adaptive' | 'constant' | 'constant' | 'adaptive' | 'constant' | 'constant' | 'constant' |
|  | **Meta model_RF** | **n_estimators** | 100 | 50 | 50 | 50 | 150 | 50 | 200 |
|  |  | **max_features** | 'sqrt' | 'auto' | 'sqrt' | 'sqrt' | 'sqrt' | 'auto' | 'auto' |
|  |  | **max_depth** | 20 | 10 | 10 | 20 | 20 | 10 | 20 |
|  |  | **min_samples_split** | 2 | 4 | 5 | 2 | 2 | 2 | 3 |
|  |  | **min_samples_leaf** | 3 | 1 | 2 | 2 | 2 | 1 | 1 |

Table S6. Optimal hyperparameters for machine learning stacked model for |NAC| prediction, obtained via grid search.

| **\|NAC\|** | | | **Pristine** | **FA_K_** | **FA_Rb_** | **FA_Cs_** | **K_i_** | **Rb_i_** | **Cs_i_** |
| --- | --- | --- | --- | --- | --- | --- | --- | --- | --- |
| **Stacking model** | **Base model_RF** | **n_estimators** | 50 | 50 | 50 | 50 | 50 | 50 | 200 |
|  |  | **max_features** | 'sqrt' | 'sqrt' | 'sqrt' | 'sqrt' | 'sqrt' | 'sqrt' | 'sqrt' |
|  |  | **max_depth** | 30 | 10 | 10 | 10 | 30 | 10 | 20 |
|  |  | **min_samples_split** | 5 | 3 | 4 | 2 | 4 | 2 | 2 |
|  |  | **min_samples_leaf** | 1 | 1 | 1 | 4 | 1 | 3 | 3 |
|  | **Base model_MLP** | **hidden_layer_sizes** | (150, 150) | (50, 50) | (50, 50) | (150, 150) | (100, 100) | (150, 150) | (50, 50) |
|  |  | **max_iter** | 600 | 400 | 600 | 600 | 600 | 400 | 600 |
|  |  | **activation** | 'relu' | 'relu' | 'relu' | 'tanh' | 'relu' | 'relu' | 'relu' |
|  |  | **solver** | 'sgd' | 'adam' | 'adam' | 'sgd' | 'adam' | 'adam' | 'adam' |
|  |  | **alpha** | 0.1 | 0.0001 | 0.1 | 0.1 | 0.001 | 0.001 | 0.1 |
|  |  | **learning_rate** | 'adaptive' | 'constant' | 'constant' | 'adaptive' | 'constant' | 'constant' | 'constant' |
|  | **Meta model_RF** | **n_estimators** | 50 | 50 | 50 | 50 | 50 | 50 | 200 |
|  |  | **max_features** | 'sqrt' | 'sqrt' | 'sqrt' | 'sqrt' | 'sqrt' | 'sqrt' | 'sqrt' |
|  |  | **max_depth** | 30 | 10 | 10 | 10 | 30 | 10 | 20 |
|  |  | **min_samples_split** | 5 | 3 | 4 | 2 | 4 | 2 | 2 |
|  |  | **min_samples_leaf** | 1 | 1 | 1 | 4 | 1 | 3 | 3 |

## Section S1.5: Equations of the Hefei-NAMD-S Framework

The defining equations of the stacked model within the Hefei-NAMD-S framework are as follows:

1. Definition of the train set for the base models:

|  |  | $D_{Base}=\left( x_{1}, y_{1} \right), \left( x_{2}, y_{2} \right), \left( x_{3}, y_{3} \right),\ldots, (x_{n}, y_{n})$ | (2) |
| --- | --- | --- | --- |

The predicted results of the base models RF and MLP:

|  |  | $P_{RF}={B_{RF}(x}_{1}),{B_{RF}(x}_{2}), {B_{RF}(x}_{3}),\ldots, {B_{RF}(x}_{n})$ | (3) |
| --- | --- | --- | --- |
|  |  | $P_{MLP}={B_{MLP}(x}_{1}),{B_{MLP}(x}_{2}), {B_{MLP}(x}_{3}),\ldots, {B_{MLP}(x}_{n})$ | (4) |

1. If the base model RF algorithm consists of *k* decision trees, denoted as $T_{1}^{Base},T_{2}^{Base},T_{3}^{Base},\ldots,T_{k}^{Base}$, the prediction value is given by:

|  |  | $B_{RF}\left( x \right)=\frac{1}{k}\sum_{j=1}^{k} T_{j}^{Base}(x)$ | (5) |
| --- | --- | --- | --- |

Here, $T_{j}\left( x \right)$ represents the prediction result of the *j*-th decision tree for the input *x*.

1. If the base model MLP algorithm consists of *L* layers of neurons, the forward propagation expression for the *l*-th layer is given by:

|  | $B_{MLP}\left( x \right)$ | $=\mathcal{z}^{L}$ | (6) |
| --- | --- | --- | --- |
|  |  | $=f^{l}\left( \mathcal{w}^{l}\cdot\mathcal{z}^{l-1}+b^{l} \right) l=1, 2, 3,\ldots, L$ | (7) |

Here, $\mathcal{z}^{L}$ represents the output prediction of the outermost layer (the *L*-th layer); $f^{l}$ is the activation function of the *l*-th layer; $\mathcal{w}^{l}$ and $b^{l}$ are the weights and biases of the *l*-th layer, respectively. $\mathcal{z}^{l-1}$ denotes the output of the preceding layer(i.e., the (*l-1*)-th layer).

1. The train set for the meta model is constructed based on the outputs of the base models.

$$D_{Meta}=\left( B_{RF}\left( x_{1} \right),B_{MLP}\left( x_{1} \right),y_{1} \right),\ldots,\left( B_{RF}\left( x_{n} \right),B_{MLP}\left( x_{n} \right),y_{n} \right)$$

If the meta model consists of *K* decision trees, denoted as $T_{1}^{Meta},T_{2}^{Meta},T_{3}^{Meta},\ldots,T_{K}^{Meta}$, the prediction value is given by:

|  | $M_{RF}\left( x \right)$ | $=M(B_{RF}\left( x \right), B_{MLP}\left( x \right))$ | (8) |
| --- | --- | --- | --- |
|  |  | $=\frac{1}{K}\sum_{i=1}^{K} T_{i}^{Meta}(B_{RF}\left( x \right), B_{MLP}\left( x \right))$ | (9) |

1. Based on the above computational formulas, the calculation formula for the stacked model within the Hefei-NAMD-S framework is given by:

|  | $S\left( x \right)$ | $=M_{RF}\left( x \right)$ | (10) |
| --- | --- | --- | --- |
|  |  | $=M(B_{RF}\left( x \right), B_{MLP}\left( x \right))$ | (11) |
|  |  | $=\frac{1}{K}\sum_{i=1}^{K} T_{i}^{Meta}(B_{RF}\left( x \right), B_{MLP}\left( x \right))$ | (12) |
|  |  | $=\frac{1}{K}\sum_{i=1}^{K} T_{i}^{Meta}(\frac{1}{k}\sum_{j=1}^{k} T_{j}^{Base},f^{l}(\mathcal{w}^{l}\cdot\mathcal{z}^{l-1}+b^{l}))$ | (13) |

## Section S1.6: Scoring Methods for Evaluation Metrics

To evaluate the predictive performance of the stacked model on regression tasks, the R^2^, RMSE, and MAE were selected as the evaluation metrics, which are defined by the following formulas:

|  | $R^{2}=1-\frac{\sum_{i=1}^{n} {(y_{i}-x_{i})}^{2}}{\sum_{i=1}^{n} {(x_{i}-\bar{x})}^{2}}$ | (14) |
| --- | --- | --- |
|  | $RMSE=\sqrt{\frac{1}{n}\sum_{i=1}^{n} {(y_{i}-x_{i})}^{2}}$ | (15) |
|  | $MAE=\frac{1}{n}\sum_{i=1}^{n} \left\vert y_{i}-x_{i} \right\vert$ | (16) |

Here, *x_i_* and *y_i_* represent the calculated and predicted values of the target properties (VBM, CBM, and |NAC|) used in this work, and $\bar{x}$ denotes the mean value of the calculated target properties. The evaluation range of R^2^ is [0, 1], where a value closer to 1 indicates better fitting performance of the model. If R^2^ is less than 0, it indicates that the predictive ability of the model is even worse than simply using the mean value. RMSE is used to measure the standard deviation of the prediction errors, being more sensitive to outliers. In contrast, MAE reflects the average deviation between the predicted and calculated values, is less sensitive to outliers, and is more suitable for evaluating the average level of prediction errors. By employing these evaluation metrics, a more comprehensive assessment of the predictive performance of the ML models can be achieved.

Table S7. The prediction performance for the three target properties was assessed using the coefficient of determination (R^2^), root mean square error (RMSE) and mean absolute error (MAE) on the train (inside the parentheses)and test sets (outside the parentheses) of the pristine system, doped systems, and the validation system (TiO_2_).

|  |  | **R^2^** | **RMSE** | **MAE** |
| --- | --- | --- | --- | --- |
| **Pristine** | **VBM** | 0.99 (0.98) | 0.02 (0.03) | 0.01 (0.03) |
|  | **CBM** | 0.98 (0.97) | 0.03 (0.04) | 0.02 (0.03) |
|  | **\|NAC\|** | 0.94 (0.90) | 0.06 (0.07) | 0.04 (0.05) |
| **FA_K_** | **VBM** | 0.99 (0.99) | 0.02 (0.03) | 0.02 (0.02) |
|  | **CBM** | 0.98 (0.95) | 0.03 (0.04) | 0.02 (0.03) |
|  | **\|NAC\|** | 0.85 (0.70) | 0.09 (0.12) | 0.07 (0.09) |
| **FA_Rb_** | **VBM** | 0.99 (0.98) | 0.02 (0.03) | 0.01 (0.02) |
|  | **CBM** | 0.99 (0.97) | 0.02 (0.04) | 0.02 (0.03) |
|  | **\|NAC\|** | 0.92 (0.81) | 0.06 (0.10) | 0.05 (0.08) |
| **FA_Cs_** | **VBM** | 0.99 (0.99) | 0.03 (0.05) | 0.02 (0.03) |
|  | **CBM** | 0.99 (0.98) | 0.02 (0.03) | 0.02 (0.02) |
|  | **\|NAC\|** | 0.86 (0.78) | 0.08 (0.10) | 0.06 (0.08) |
| **K_i_** | **VBM** | (0.98) | (0.03) | (0.02) |
|  | **CBM** | (0.98) | (0.02) | (0.02) |
|  | **\|NAC\|** | (0.80) | (0.10) | (0.08) |
| **Rb_i_** | **VBM** | (0.99) | (0.02) | (0.01) |
|  | **CBM** | (0.99) | (0.03) | (0.02) |
|  | **\|NAC\|** | (0.90) | (0.07) | (0.06) |
| **Cs_i_** | **VBM** | (0.98) | (0.03) | (0.02) |
|  | **CBM** | (0.99) | (0.03) | (0.02) |
|  | **\|NAC\|** | (0.85) | (0.07) | (0.06) |
| **TiO_2_** | **VBM** | 0.99 (0.99) | 0.02 (0.02) | 0.006 (0.007) |
|  | **VBM-1** | 0.99 (0.98) | 0.02 (0.03) | 0.010 (0.020) |
|  | **\|NAC\|** | 0.98 (0.97) | 0.03 (0.04) | 0.000 (0.010) |


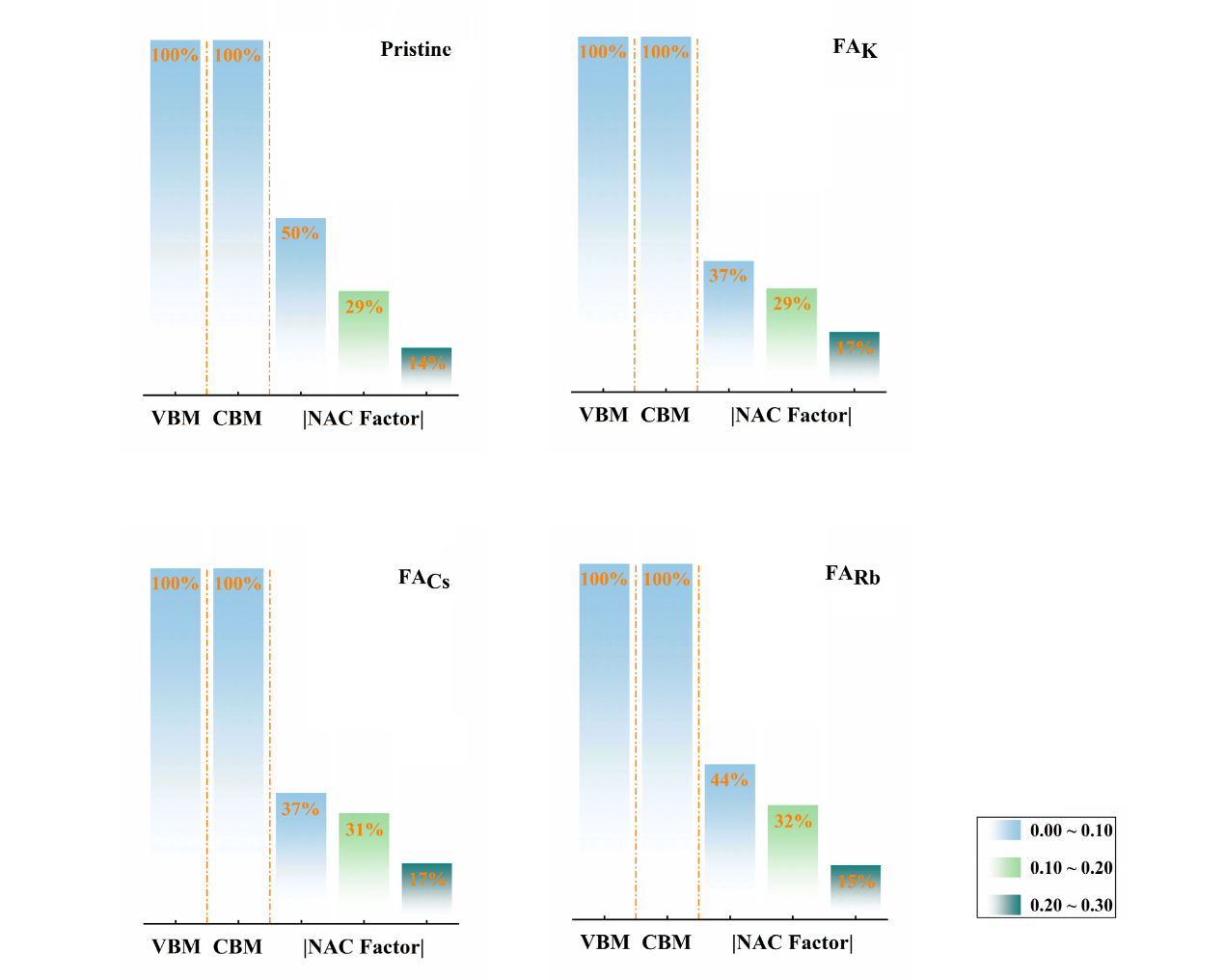


Figure S22. Histogram of the deviations between the predicted and computed values for the pristine and alkali metal substitutionally doped perovskite systems.

## Section S1.7: Non-adiabatic Molecular Dynamics Simulations

The training of the stacked model was completed using 500 D_Train_ data points, followed by the input of 1500 D_Test_ data points to obtain the predicted values of the target properties. Considering that normalization was applied during feature engineering, the predicted values were subsequently inverse normalized to recover the predicted values in the original scale. Then, according to the equally spaced sampling rule used for data extraction, the predicted values corresponding to D_Train_ and D_Test_ were reinserted into the dynamical trajectory in their original temporal order, thereby reconstructing a complete and continuous trajectory. NAMD simulations for FAPbI_3_ and its alkali metal doped systems were performed using the Hefei-NAMD package.

|  | $\vert NAC\vert=\vert\left\langle\varphi_{m}\left\vert\frac{\partial}{\partial t} \right\vert\varphi_{n} \right\rangle\vert=\vert\frac{\left\langle\varphi_{m}\left\vert\nabla_{R}H \right\vert\varphi_{n} \right\rangle}{\varepsilon_{m}-\varepsilon_{n}}\dot{R\vert}$ | (17) |
| --- | --- | --- |
|  | $NAC=\bar{\sum\vert NAC\vert}\cdot\frac{\hbar}{2}$ | (18) |

Here, |NAC| refers to the absolute value of the NAC, which determines the probability of hopping between electronic states. $\varphi_{m}$，$\varphi_{n}$，$\varepsilon_{m}$ and $\varepsilon_{n}$ represent the wavefunctions and eigenvalues of the initial state *m* and final state *n*, respectively, and *H* denotes the Hamiltonian of the Kohn-Sham orbitals. $\left\langle\varphi_{m}\left| \nabla_{R}H \right|\varphi_{n} \right\rangle$ corresponds to the electron-phonon coupling strength, ${(\varepsilon}_{m}-\varepsilon_{n})$ represents the energy difference between Kohn-Sham orbitals, and $\dot{R}$ denotes the nuclear velocity.^[5]^ According to equation,^[6]^ NAC is calculated based on the overlap between two Kohn-Sham orbital levels at adjacent ionic time steps.

Table S8. Non-adiabatic coupling (meV), pure-dephasing time (fs), and non-radiative electron-hole recombination time (ns) in pristine and doped systems.

|  | **With Hefei-NAMD-S** | |  | **Without Hefei-NAMD-S** | |  | Recombination time |
| --- | --- | --- | --- | --- | --- | --- | --- |
|  | NAC | Pure-dephasing time |  | NAC | Pure-dephasing time |  |  |
| **Pristine** | 0.3078 | 16.5528 |  | 0.3108 | 16.7353 |  | 99.8600 |
| **FA_K_** | 0.2593 | 13.2594 |  | 0.2611 | 13.2374 |  | 156.9300 |
| **FA_Rb_** | 0.2872 | 13.4859 |  | 0.2867 | 13.3910 |  | 280.8000 |
| **FA_Cs_** | 0.2727 | 9.9791 |  | 0.2698 | 10.0166 |  | 91.1600 |
| **K_i_** | 0.2681 | 15.3633 |  |  |  |  | 97.3600 |
| **Rb_i_** | 0.3652 | 7.9040 |  |  |  |  | 31.8300 |
| **Cs_i_** | 0.2260 | 13.9430 |  |  |  |  | 271.5900 |

# Section S2: First-principles Calculation

## Section S2.1: Computational Methods for Electronic Structure

Crystal structure optimization, electronic structure calculations, and adiabatic molecular dynamics simulations for FAPbI_3_ and its doped systems were performed using the Vienna *ab initio* Simulation Package.^[7-8]^ The exchange-correlation effects were described using the Perdew-Burke-Ernzerhof (PBE) functional within the framework of the generalized gradient approximation.^[9]^ Although the PBE functional is known to underestimate the bandgap, it was selected in this work for the following reasons. First, numerous studies have shown that the bandgap obtained by combining spin-orbit coupling (SOC) with the GW method is comparable to that the PBE functional, due to the mutual cancellation of the effects introduced by SOC and GW corrections.^[10-13]^ Furthermore, we additionally calculated the band gap of FAPbI_3_ using the PBE0+SOC approach. The results show that the band gap obtained from PBE0+SOC is 1.53 eV, differing from the experimental value of 1.48 eV by only 0.05 eV. In comparison, the band gap calculated using PBE is 1.36 eV, with a deviation of 0.12 eV from the experimental value. The relatively small discrepancies between the calculated and experimental band gaps obtained from both methods indicate that, although the PBE method slightly underestimates the band gap, it can still reasonably describe the relative changes in electronic structure among different systems. Second, considering the large number of systems studied and the need to solve tens of thousands of Kohn-Sham equations, applying the SOC+GW or PBE0+SOC method during the adiabatic molecular dynamics process would impose enormous challenges in terms of computational resources and time costs.^[14-15]^ The electron-nucleus interactions were treated using the projector-augmented wave method,^[16-17]^ and the single-particle wavefunctions were expanded on a plane-wave basis set with a cutoff energy of 450 eV. Brillouin zone sampling was conducted using a *k*-point grid with an accuracy of 0.03×2π/Å within the Gamma scheme. The van der Waals interactions were accounted for using the DFT-D3 correction proposed by Grimme *et al*.^[18]^ All perovskite systems were optimized using the conjugate gradient algorithm, with the energy convergence criterion set to 10^-5^ eV and the Hellmann-Feynman force convergence criterion set to 0.015 eV/Å. During the adiabatic molecular dynamics process, the energy convergence criterion was tightened to 10^-8^ eV to more accurately capture the strong anharmonic of the perovskite systems.

Table S9. Lattice parameters of the 2×2×2 supercells for pristine and doped systems.

|  | **a (Å)** | **b (Å)** | **c (Å)** | ***α* (°)** | ***β* (°)** | ***γ* (°)** | **Volume (Å^3^)** |
| --- | --- | --- | --- | --- | --- | --- | --- |
| **Pristine** | 12.58 | 12.73 | 12.93 | 90.00 | | | 2069.91 |
| **FA_K_** | 12.62 | 12.60 | 12.62 |  |  |  | 2007.23 |
| **FA_Rb_** | 12.63 | 12.60 | 12.65 |  |  |  | 2013.78 |
| **FA_Cs_** | 12.67 | 12.61 | 12.69 |  |  |  | 2027.15 |
| **K_i_** | 12.48 | 12.83 | 12.99 |  |  |  | 2078.21 |
| **Rb_i_** | 12.51 | 12.82 | 13.02 |  |  |  | 2087.50 |
| **Cs_i_** | 12.53 | 12.81 | 13.06 |  |  |  | 2097.26 |


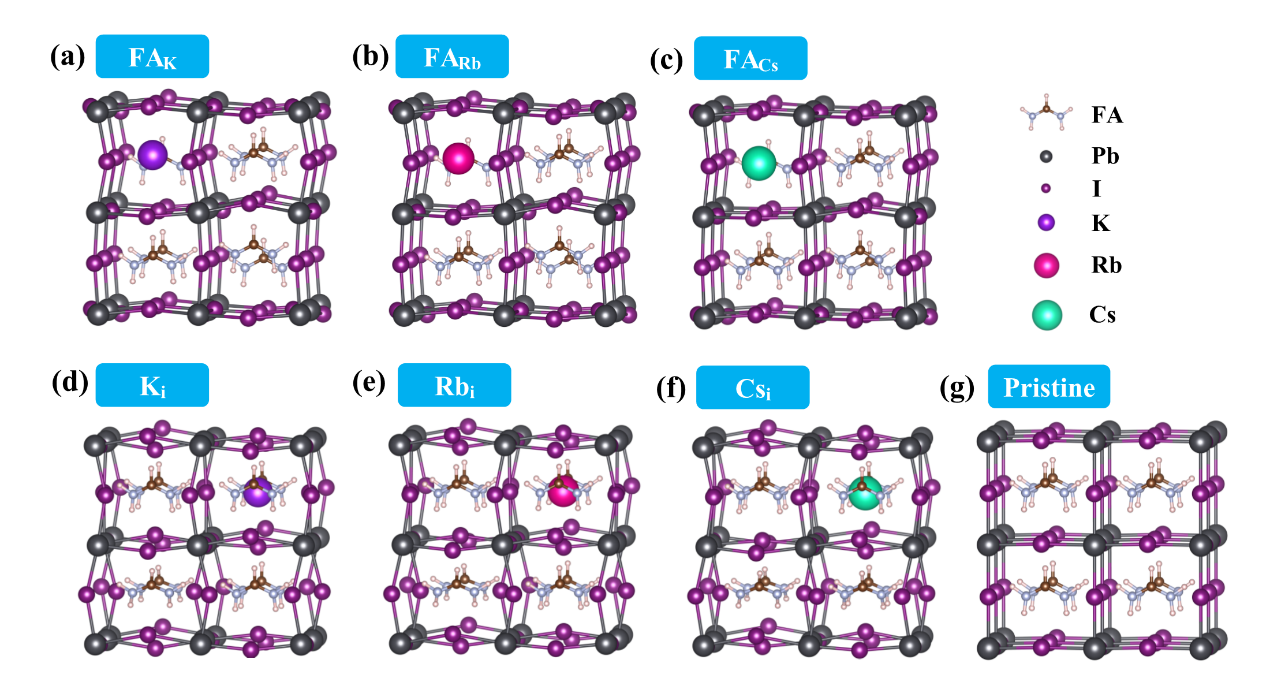


Figure S23. Optimized crystal structures of the pristine and doped systems at 0 K.


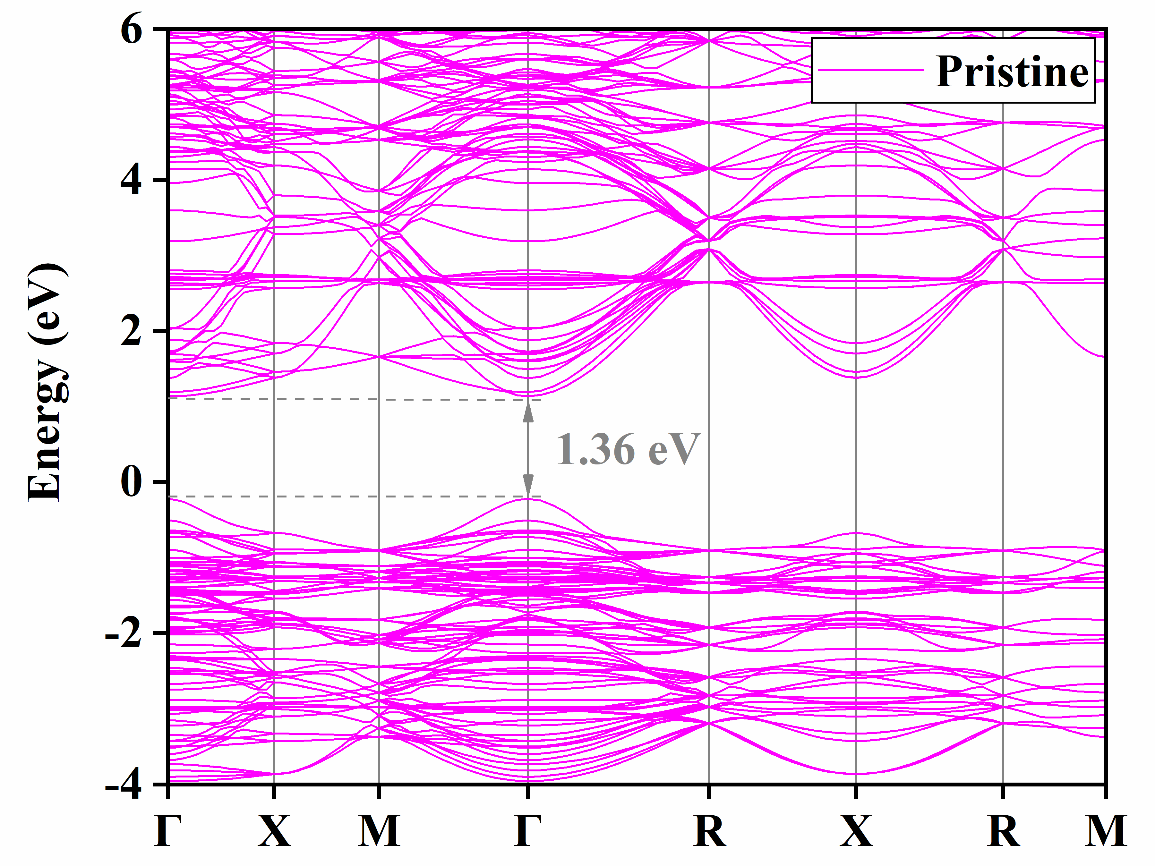


Figure S24. Band structure of the pristine system, with the Fermi level set as the energy reference.


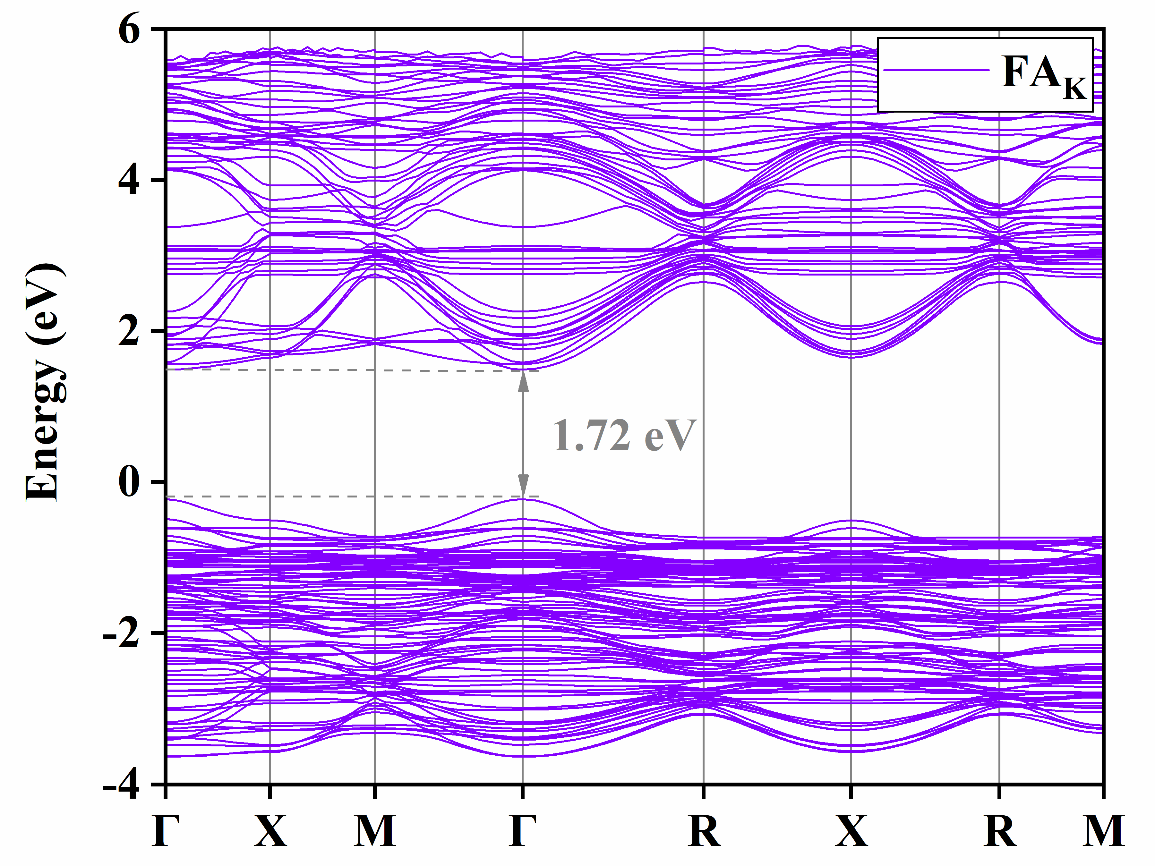


Figure S25. Band structure of the FA_K_ system, with the Fermi level set as the energy reference.


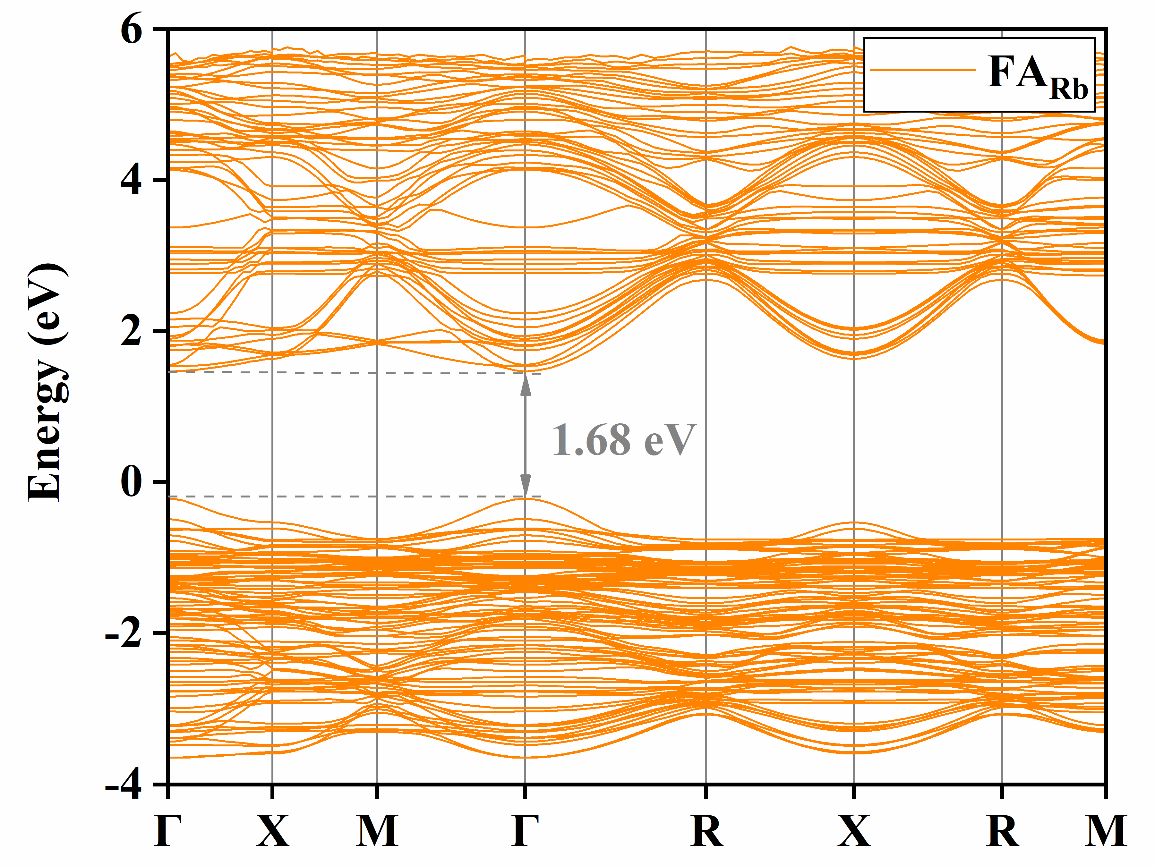


Figure S26. Band structure of the FA_Rb_ system, with the Fermi level set as the energy reference.


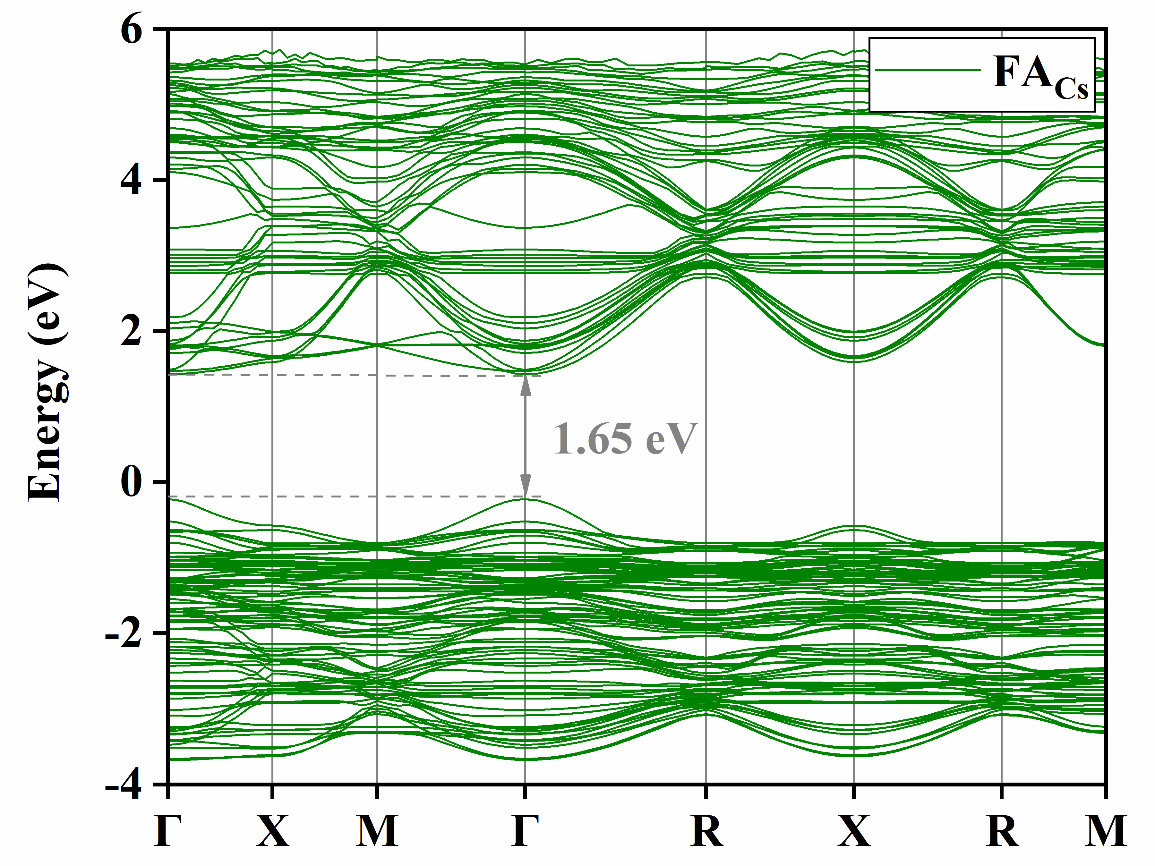


Figure S27. Band structure of the FA_Cs_ system, with the Fermi level set as the energy reference.


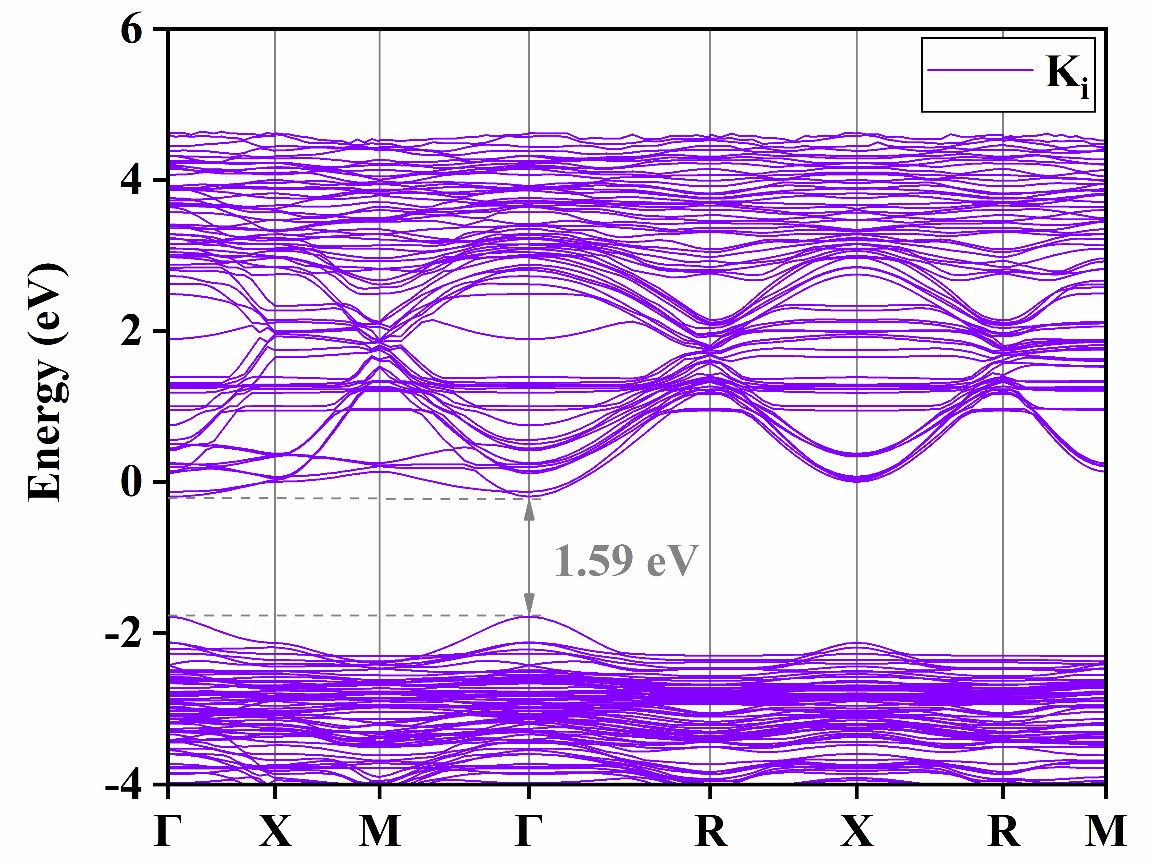


Figure S28. Band structure of the K_i_ system, with the Fermi level set as the energy reference.


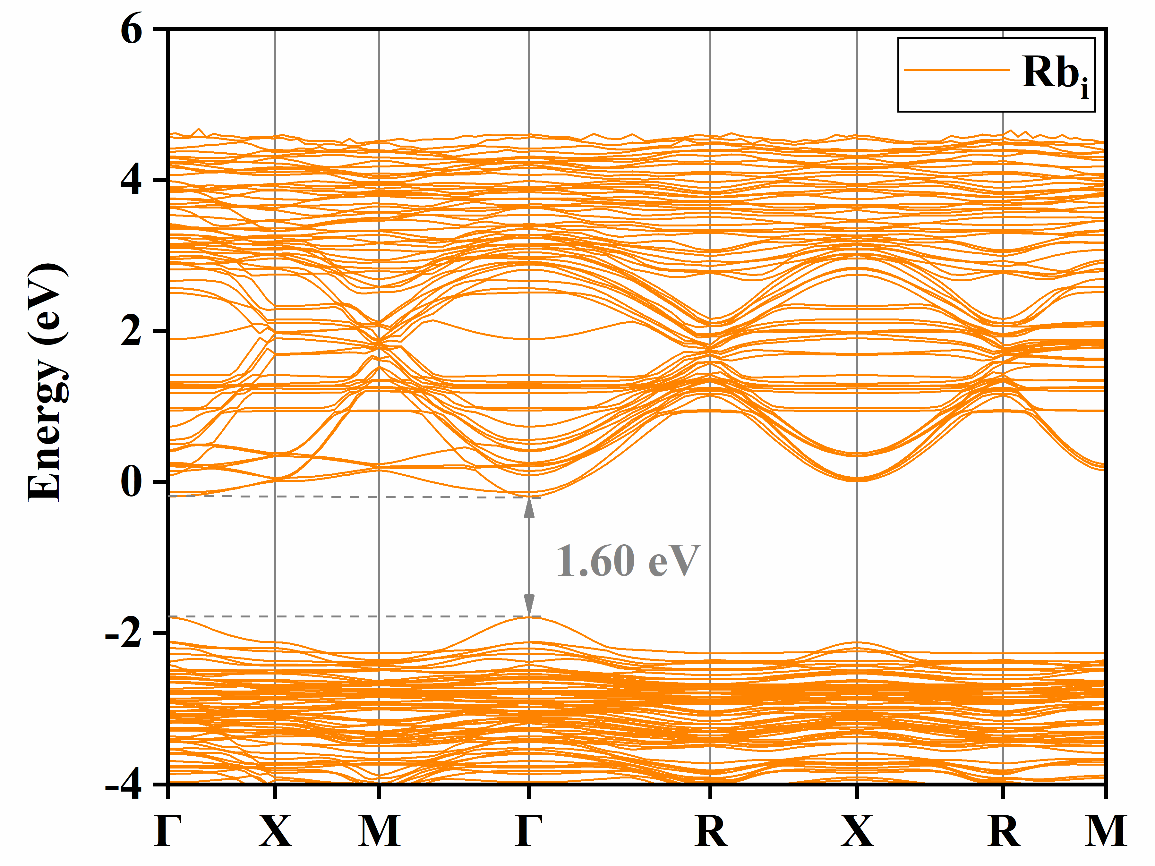


Figure S29. Band structure of the Rb_i_ system, with the Fermi level set as the energy reference.


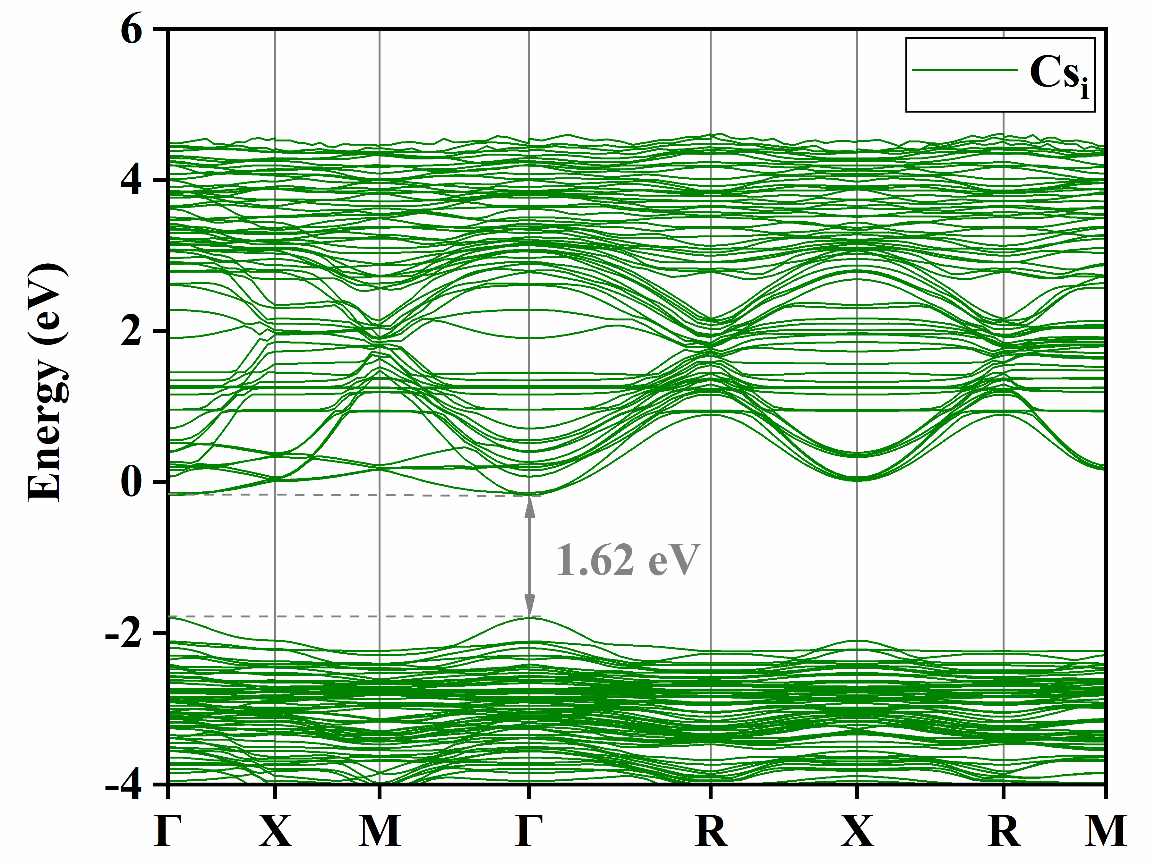


Figure S30. Band structure of the Cs_i_ system, with the Fermi level set as the energy reference.


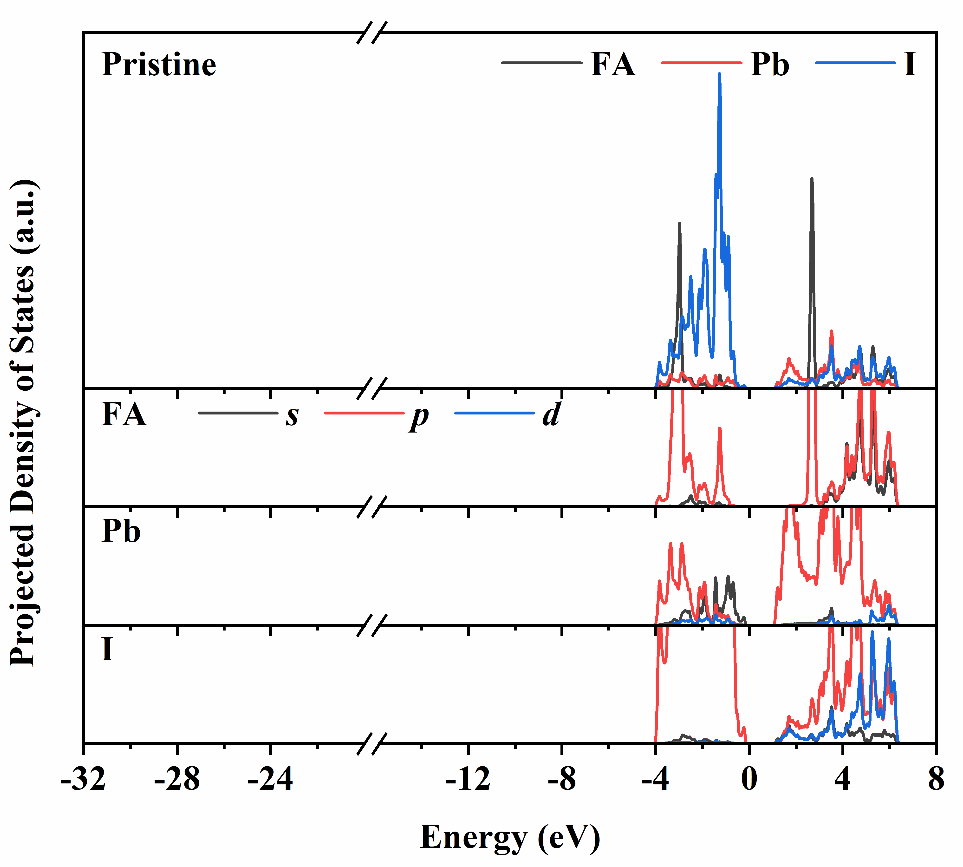


Figure S31. Projected density of states of the pristine system.


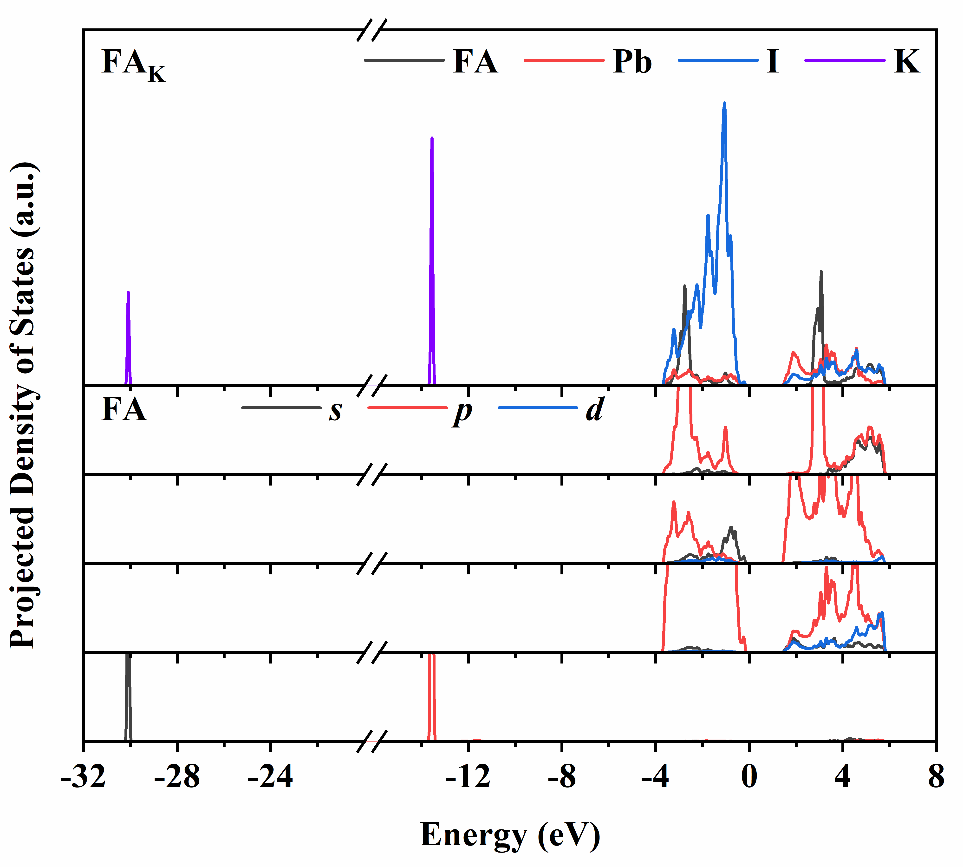


Figure S32. Projected density of states of the FA_K_ system.


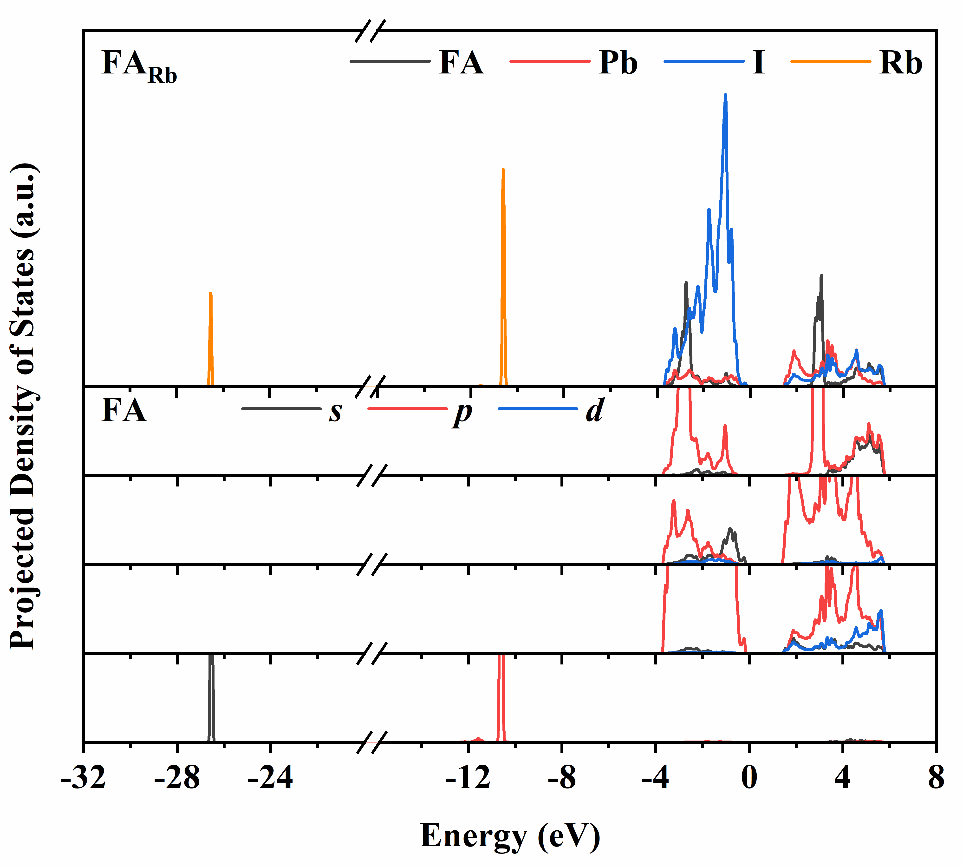


Figure S33. Projected density of states of the FA_Rb_ system.


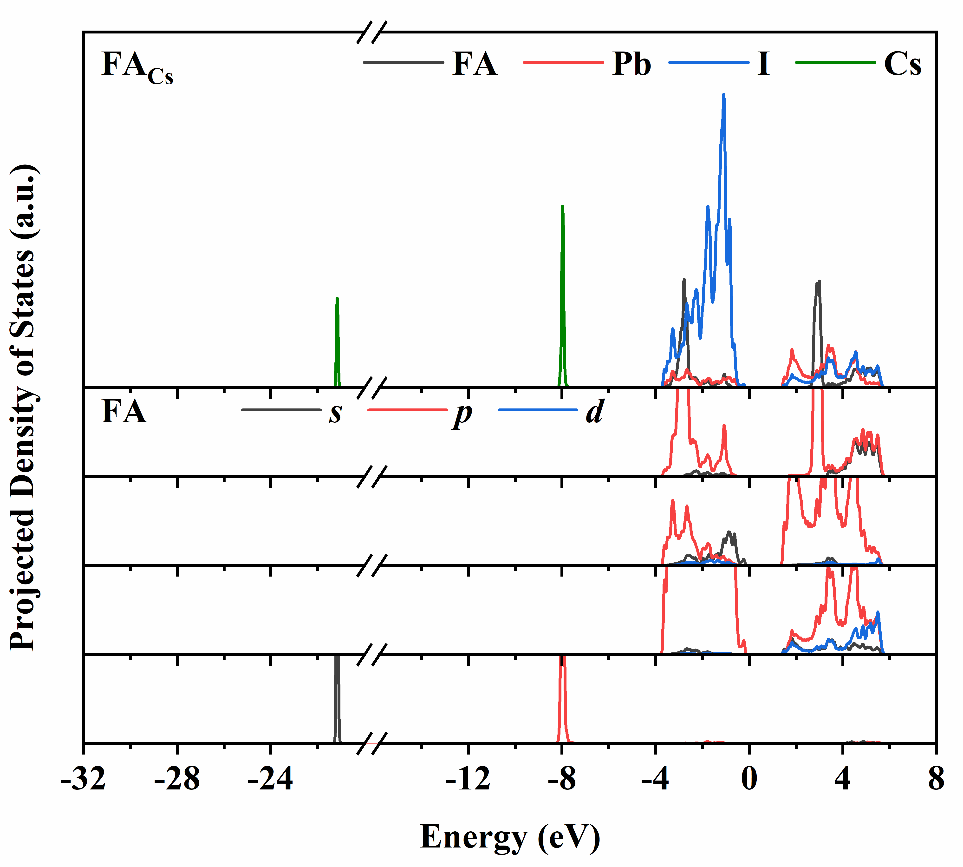


Figure S34. Projected density of states of the FA_Cs_ system.


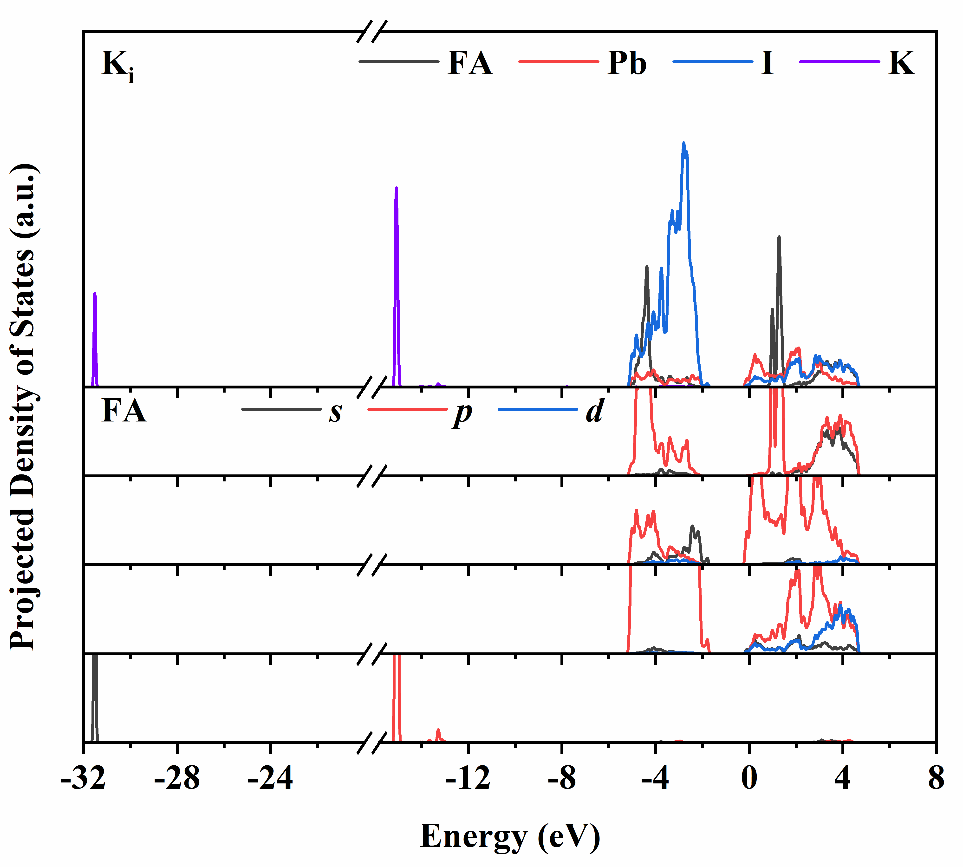


Figure S35. Projected density of states of the K_i_ system.


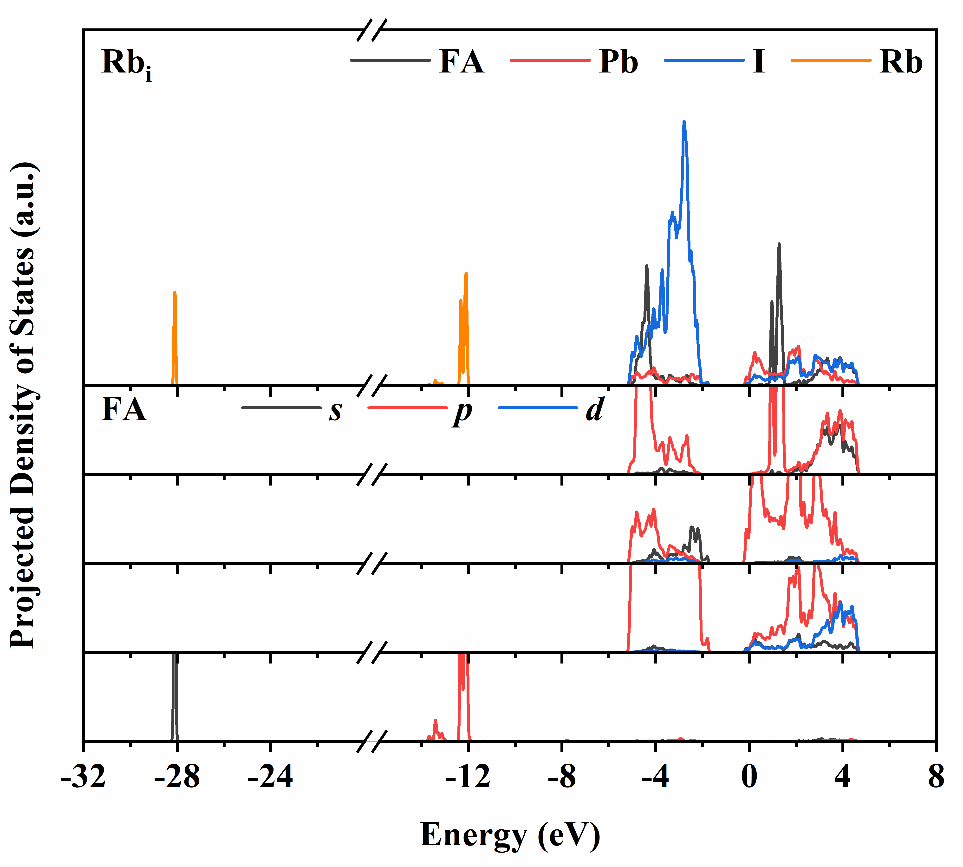


Figure S36. Projected density of states of the Rb_i_ system.


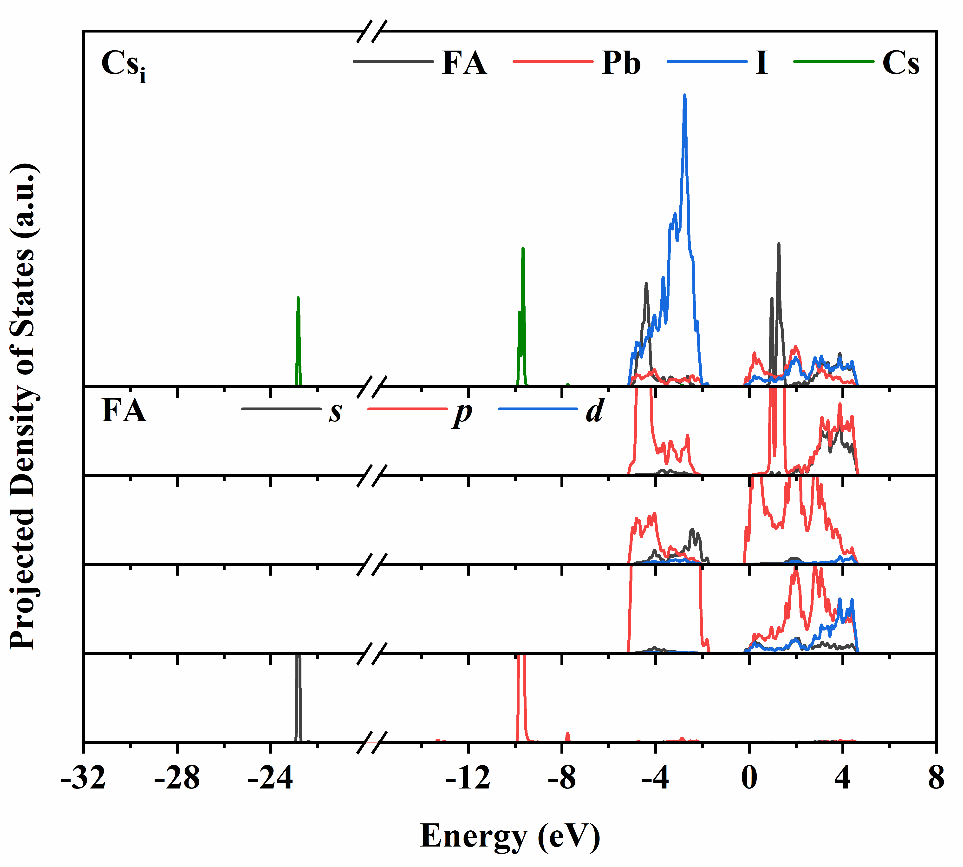


Figure S37. Projected density of states of the Cs_i_ system.

## Section S2.2: Computational Methods for Non-adiabatic Properties

Ab initio NAMD simulations were performed using the Hefei-NAMD package,^[6, 19-21]^ following the decoherence-induced surface hopping algorithm within the framework of time-dependent Kohn-Sham theory under a mixed quantum-classical scheme.^[22]^ The light and fast-moving electrons were treated quantum mechanically, whereas the heavy and slow-moving nuclei were treated classically. Before NAMD simulations, FAPbI_3_ and its doped systems were heated to 300 K using velocity scaling under the canonical ensemble. Subsequently, adiabatic molecular dynamics trajectories of 3000 fs were generated under the microcanonical ensemble with a time step of 1 fs. To eliminate fluctuations originating from the thermalization stage, the nuclear positions from the last 2000 fs were extracted, ensuring that the collected trajectories correspond to the thermally equilibrated part of the system. Finally, the NAMD simulation results were obtained by averaging over five randomly sampled initial structures, with each initial structure evolving up to 500 ns and the Hamiltonian propagated through 500 iterations.

To balance computational efficiency and dynamical accuracy, the 2000 fs adiabatic molecular dynamics trajectory was periodically extended using a head to tail stitching strategy. The trajectory already contains the essential time dependent information required for non-adiabatic molecular dynamics simulations, including energy level fluctuations, non-adiabatic couplings, and phase evolution. In this way, the effective non-adiabatic molecular dynamics evolution time was extended to 500 ns. It should be noted that direct stitching may introduce abrupt changes at the boundaries, such as discontinuities in energy levels, non-adiabatic couplings, and phases. Therefore, boundary continuity corrections were applied during the stitching process to avoid abrupt changes and ensure the stability of long-time nonadiabatic molecular dynamics simulations.

To quantitatively characterize the fluctuation amplitudes of the band edge energies and band gap along the dynamical trajectory, we calculated the standard deviations of the CBM, VBM, and instantaneous band gap (Eg) over the 2000 fs trajectory. At each time step, the Eg is defined as:

|  | $E_{g}(t)=E_{CBM}(t)-E_{VBM}(t)$ | (19) |
| --- | --- | --- |

Where $E_{CBM}(t)$ and $E_{VBM}(t)$ represent the CBM and VBM energies at time t, respectively. Subsequently, the standard deviations of $E_{CBM}(t)$, $E_{VBM}(t)$ and $E_{g}(t)$ were calculated to characterize their fluctuation amplitudes during the dynamical trajectory evolution. The standard deviation was calculated as follows:

|  | $\delta=\sqrt{\frac{1}{N}\sum_{t=1}^{N} {(E_{t}-\bar{E})}^{2}}$ | (20) |
| --- | --- | --- |

where $E_{t}$ represents the energy of $E_{CBM}(t)$, $E_{VBM}(t)$, or $E_{g}(t)$; *N* is the total number of times in the dynamical trajectory; $\bar{E}$ is the average value of the corresponding energy over the entire trajectory. Accordingly, the fluctuation amplitudes of the CBM, VBM, and Eg are denoted as δCBM, δVBM, and δEg, respectively.


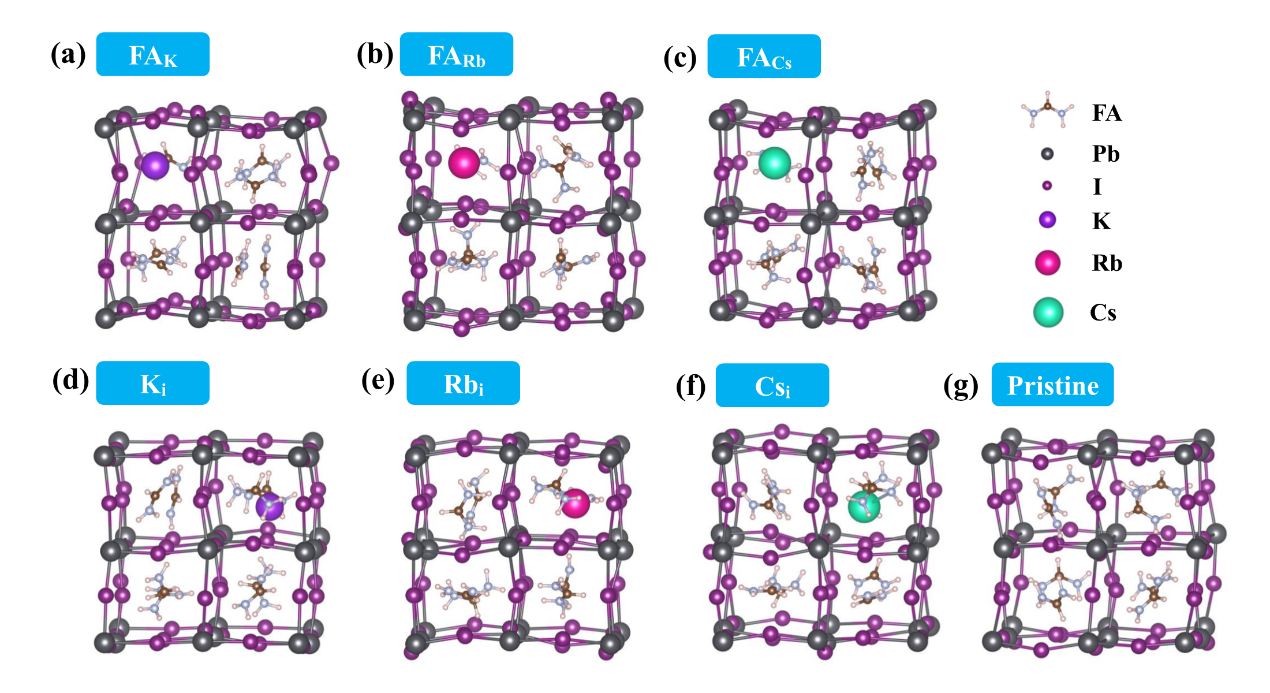


Figure S38. Crystal structures of the pristine and doped systems after canonical ensemble molecular dynamics simulations, heated to 300 K.


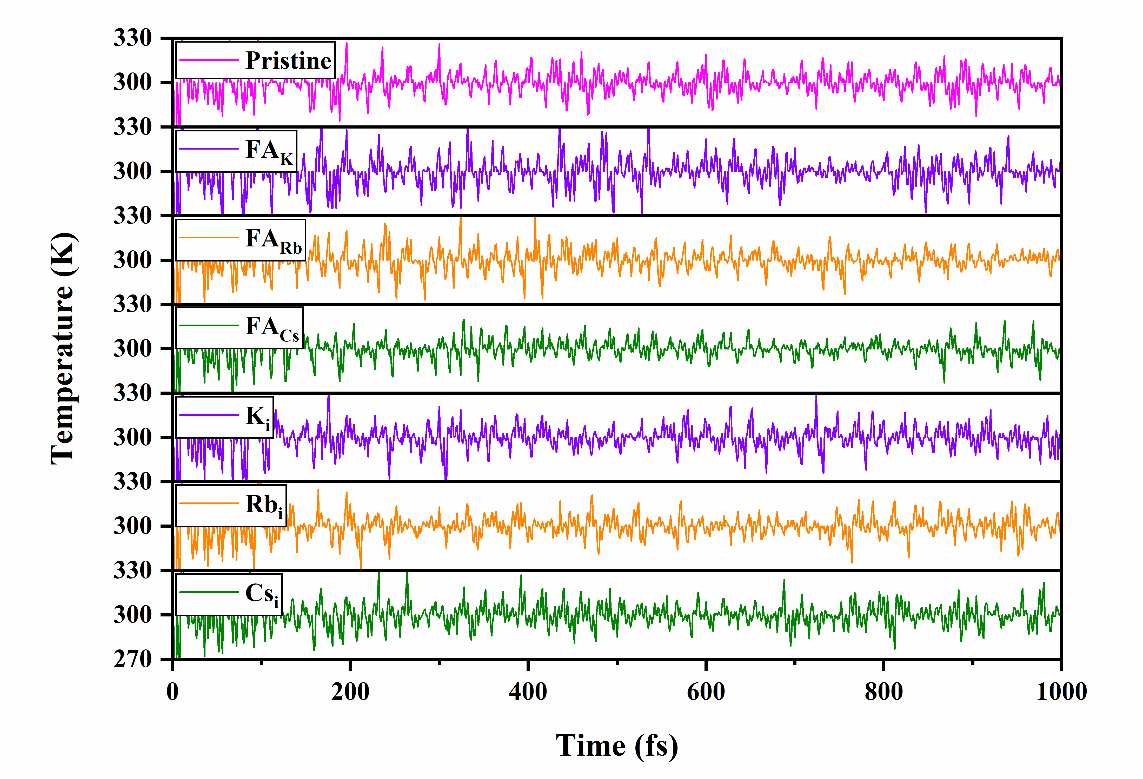


Figure S39. Temperature convergence during the canonical ensemble molecular dynamics simulations of the pristine and doped systems. The final temperature is considered converged when it falls within 10% of the target value, indicating that the heated crystal structures are reasonable.


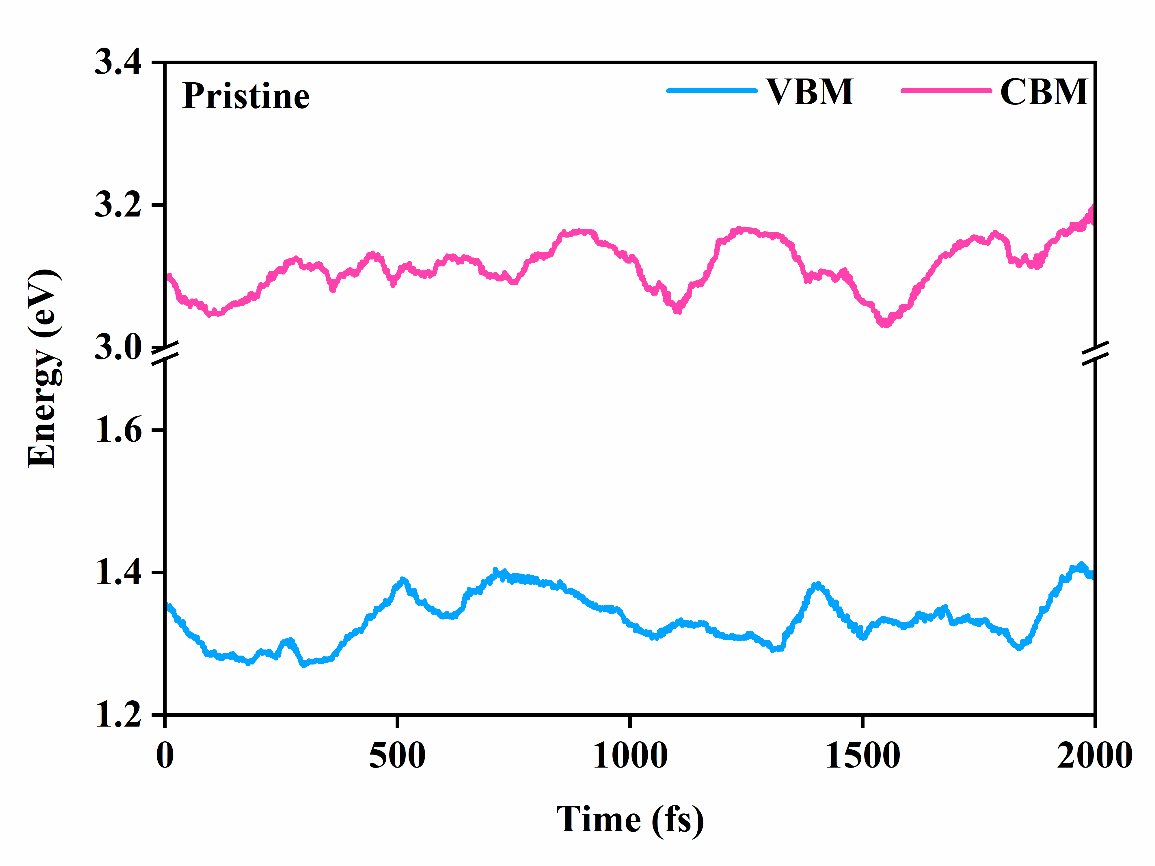


Figure S40. Based on the predictions of the stacked model, the evolution of Kohn-Sham orbital energies in the pristine system over 2000 fs.


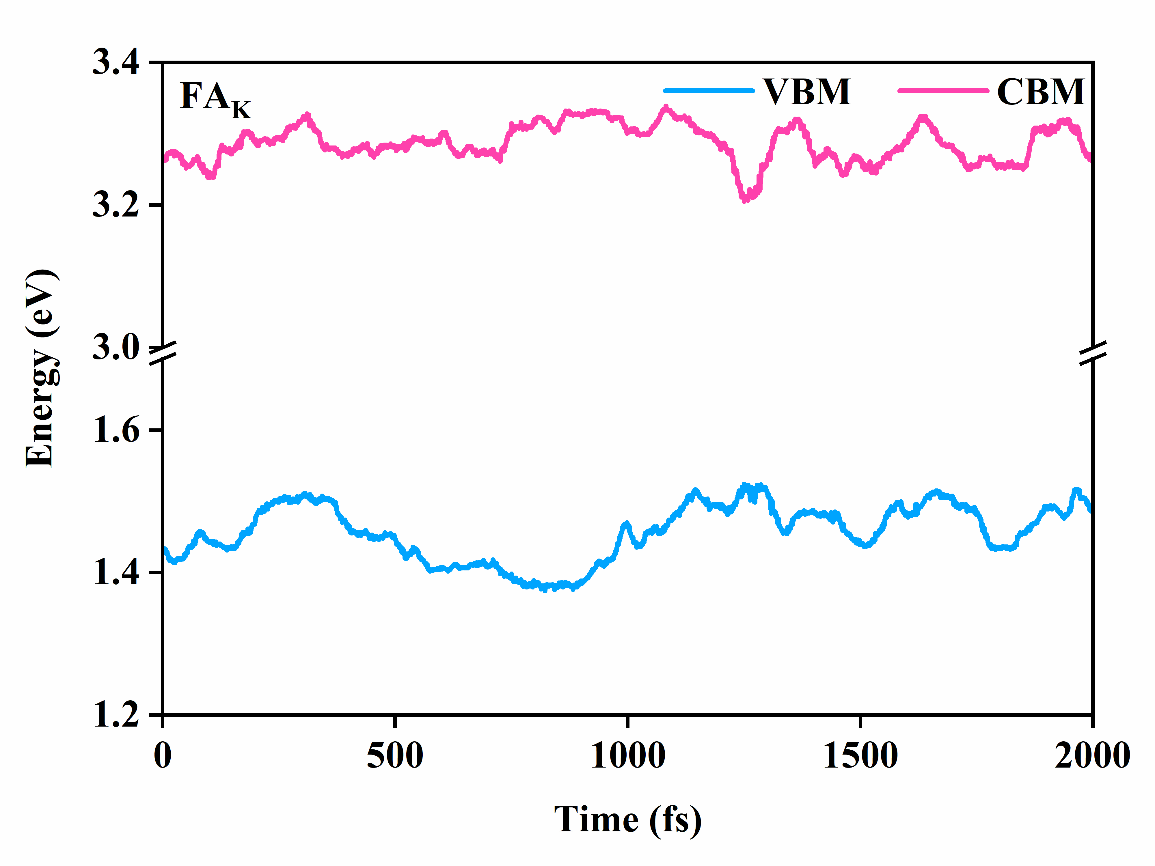


Figure S41. Based on the predictions of the stacked model, the evolution of Kohn-Sham orbital energies in the FA_K_ system over 2000 fs.


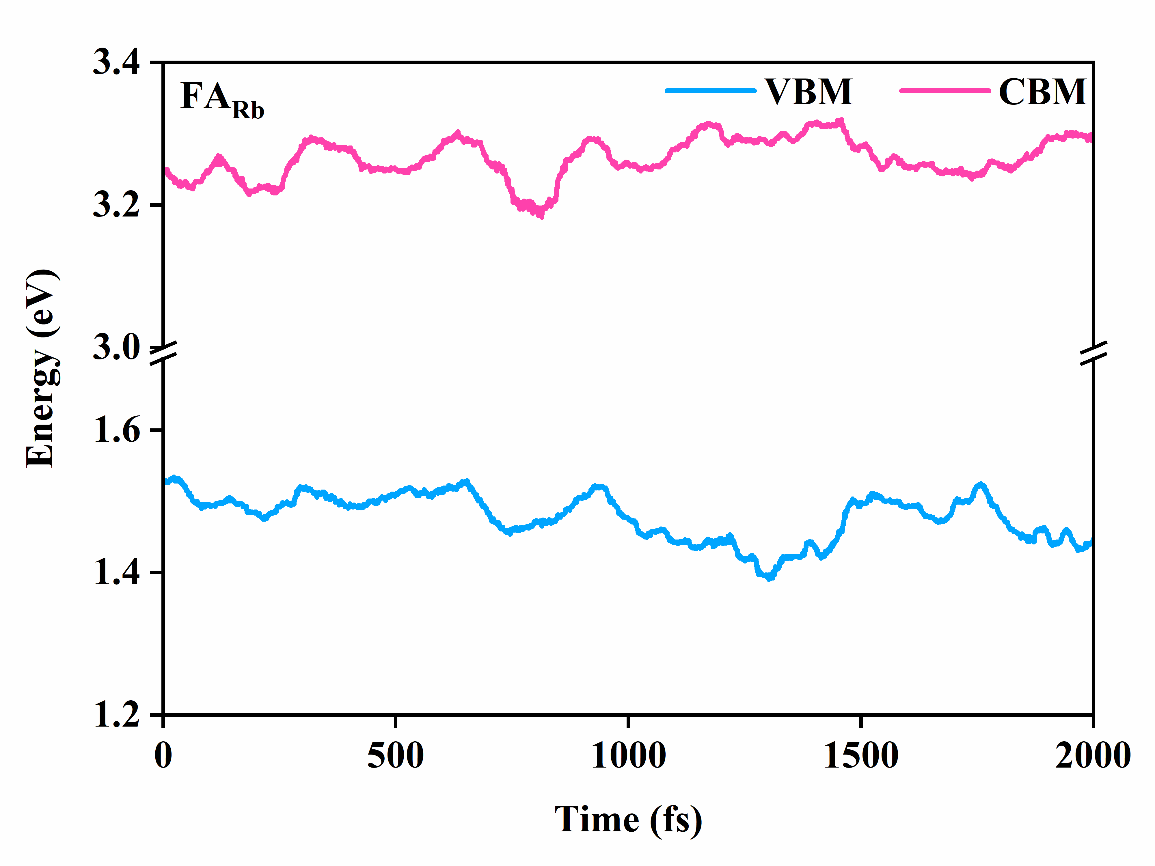


Figure S42. Based on the predictions of the stacked model, the evolution of Kohn-Sham orbital energies in the FA_Rb_ system over 2000 fs.


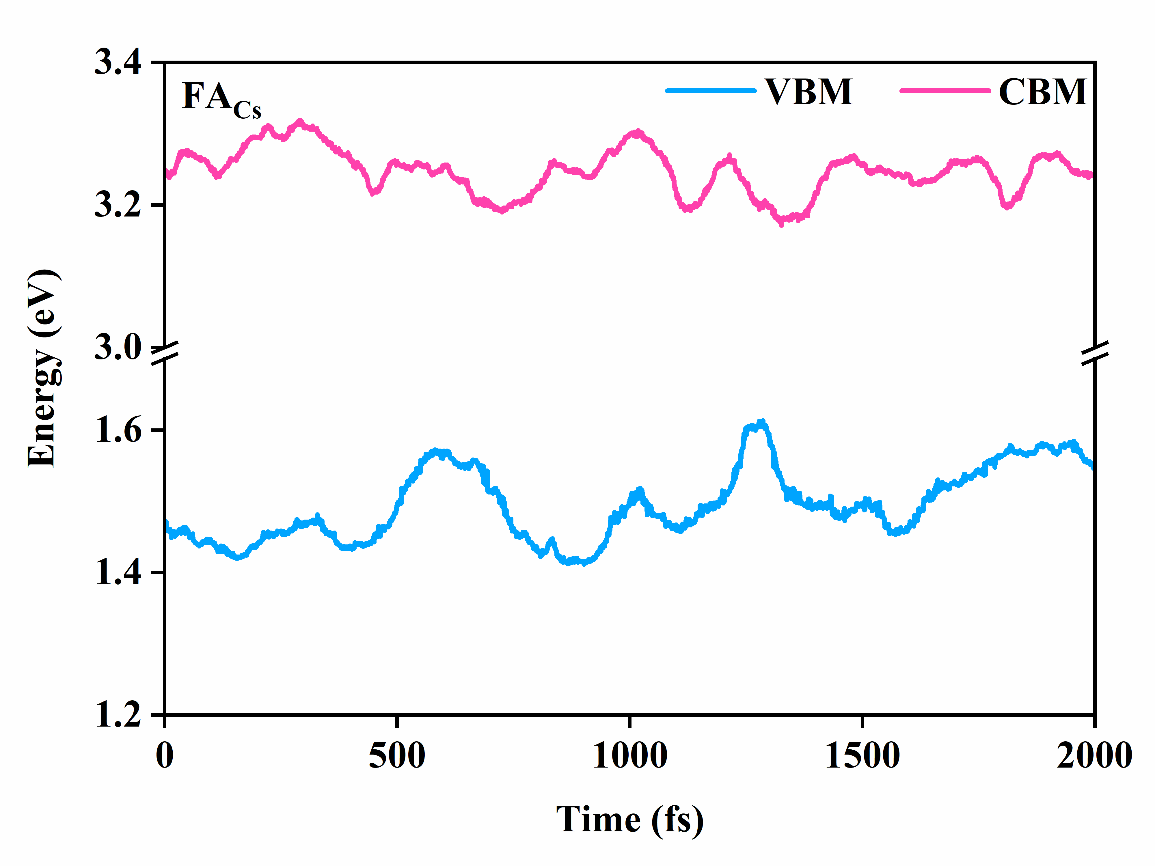


Figure S43. Based on the predictions of the stacked model, the evolution of Kohn-Sham orbital energies in the FA_Cs_ system over 2000 fs.


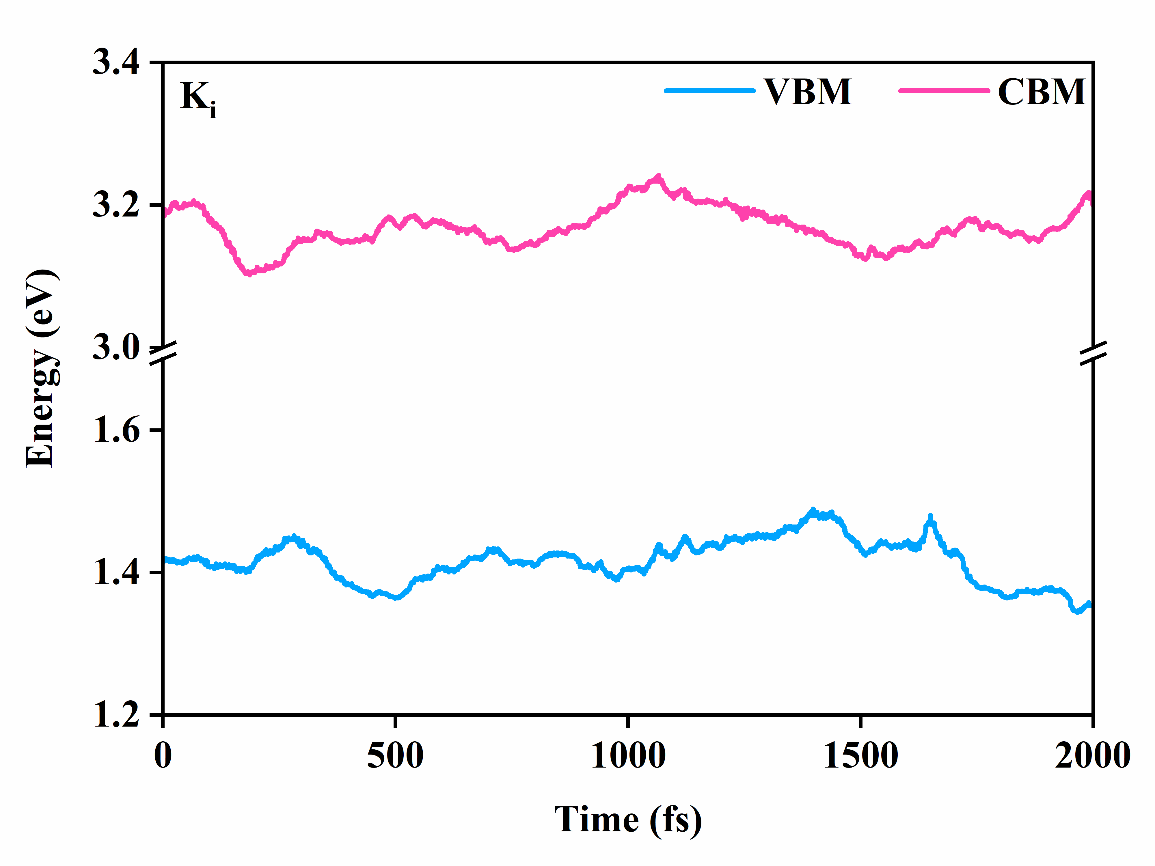


Figure S44. Based on the predictions of the stacked model, the evolution of Kohn-Sham orbital energies in the K_i_ system over 2000 fs.


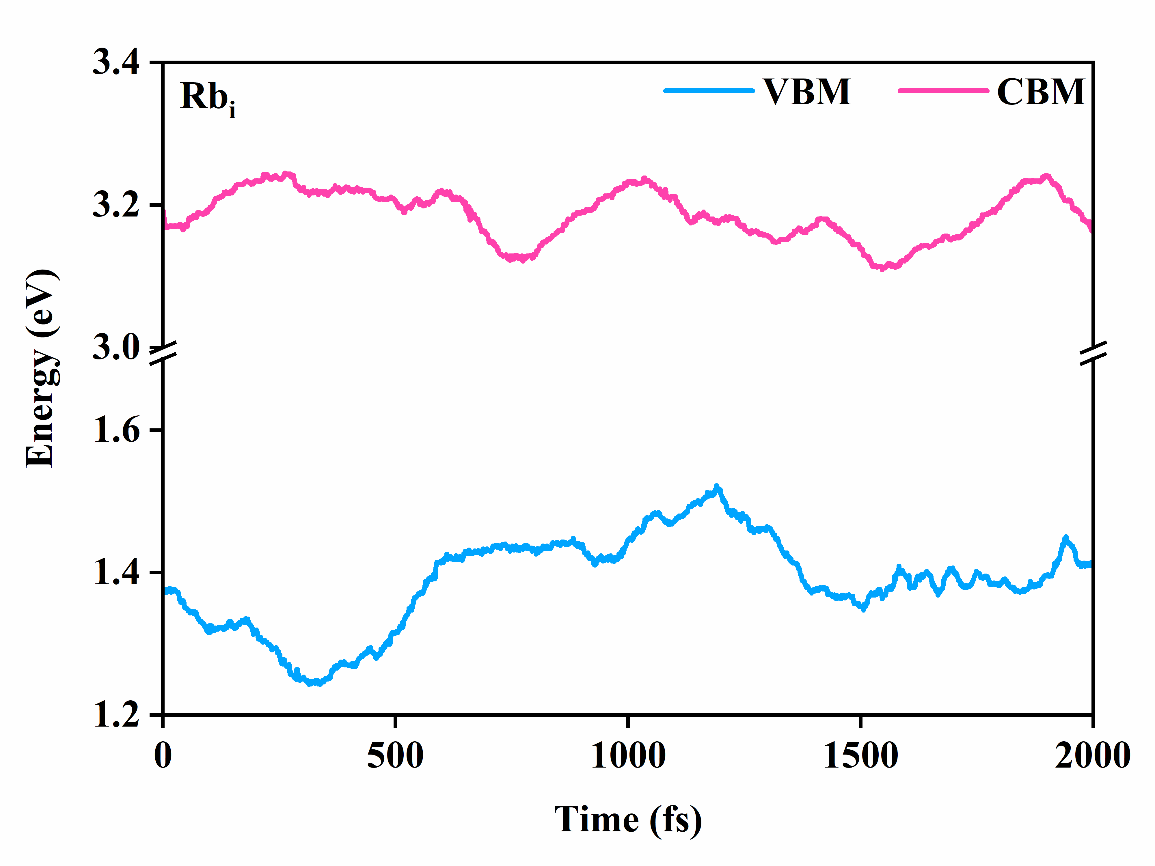


Figure S45. Based on the predictions of the stacked model, the evolution of Kohn-Sham orbital energies in the Rb_i_ system over 2000 fs.


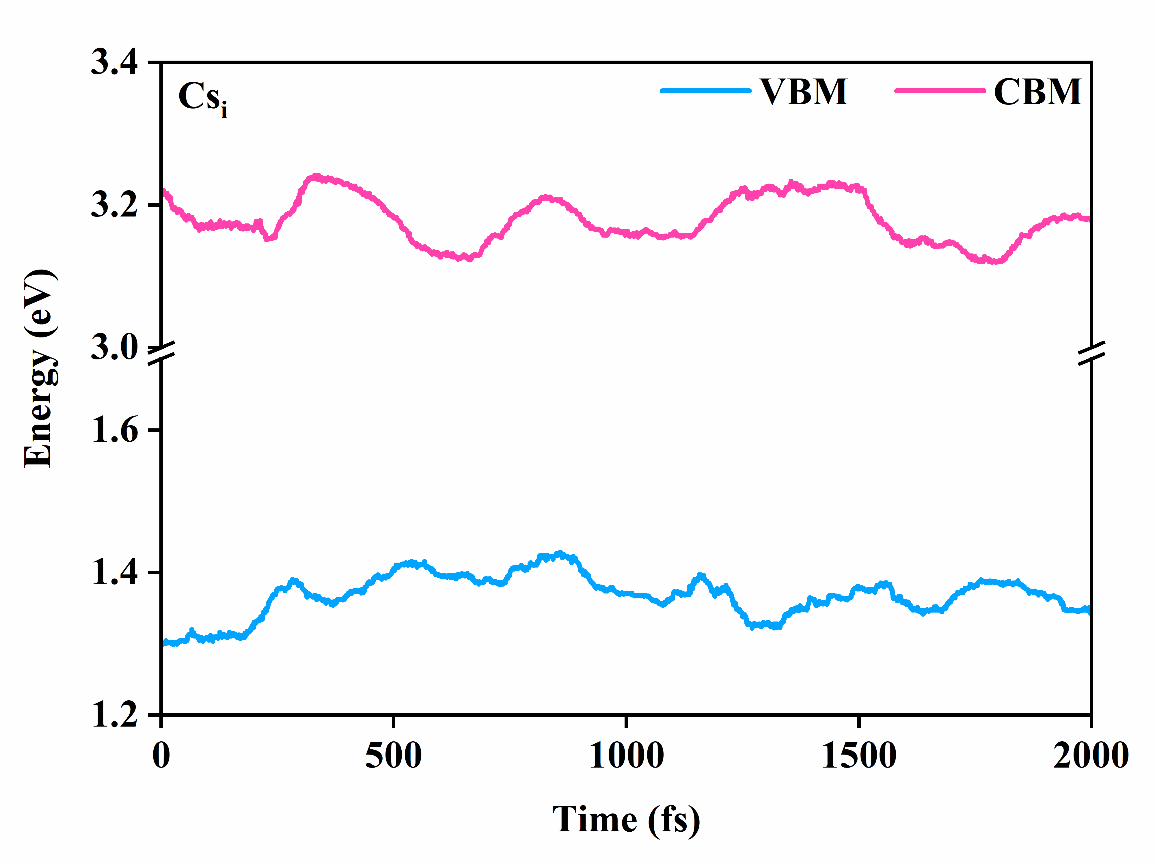


Figure S46. Based on the predictions of the stacked model, the evolution of Kohn-Sham orbital energies in the Cs_i_ system over 2000 fs.

## Section S2.3: Computational Methods for Local Polarization

The local polarization in perovskite systems essentially originates from symmetrical breaking induced by lattice distortions. To quantitatively describe the local polarization strength at the A and B sites of the perovskite lattice, the local polarization is calculated using the following expression:^[23]^

|  | $P=\frac{q*d}{V}$ | (21) |
| --- | --- | --- |

Where P denotes the local polarization strength at the A or B site; q is the Born effective charge tensor; d represents the displacement of the A or B site cation relative to the center of its corresponding cage; and V is the cage volume. Here, the cage refers to the void formed by the surrounding octahedra for the A site cation, while for the B site, it corresponds to the volume of the octahedron itself. To eliminate instantaneous local polarization arising from thermal fluctuations, the ionic positions along the 300 K molecular dynamics trajectories are averaged prior to the calculation.^[23]^

# References

[1] L. Breiman, *Mach. learn.* **2001**, 45, 5.

[2] S. B. Kotsiantis, *Artif. Intell. Rev.* **2013**, 39, 261.

[3] D. E. Rumelhart, G. E. Hinton, R. J. Williams, *Nature* **1986**, 323, 533.

[4] R. Durbin, D. E. Rumelhart, *Neural Comput.* **1989**, 1, 133.

[5] K. G. Reeves, A. Schleife, A. A. Correa, Y. Kanai, *Nano Lett.* **2015**, 15, 6429.

[6] Q. Zheng, W. Chu, C. Zhao, L. Zhang, H. Guo, Y. Wang, X. Jiang, J. Zhao, *WIREs Comput. Mol. Sci.* **2019**, 9, e1411.

[7] G. Kresse, J. Furthmuller, *Comp. Mater. Sci.* **1996**, 6, 15.

[8] G. Kresse, J. Hafner, *Phys. Rev. B* **1993**, 47, 558.

[9] J. P. Perdew, K. Burke, M. Ernzerhof, *Phys. Rev. Lett.* **1997**, 78, 1396.

[10] W. Chu, Q. Zheng, O. V. Prezhdo, J. Zhao, W. A. Saidi, *Sci. Adv.* **2020**, 6, eaaw7453.

[11] P. Umari, E. Mosconi, F. De Angelis, *Sci. Rep.* **2014**, 4, 4467.

[12] X. Zhao, H. Lu, W.-H. Fang, R. Long, *Nanoscale* **2022**, 14, 4644.

[13] L. Qiao, X. Sun, R. Long, *J. Phys. Chem. Lett.* **2019**, 10, 672.

[14] R. Long, J. Liu, O. V. Prezhdo, *J. Am. Chem. Soc.* **2016**, 138, 3884.

[15] W. Li, J. Liu, F. Q. Bai, H. X. Zhang, O. V. Prezhdo, *ACS Energy Lett.* **2017**, 2, 1270.

[16] P. E. Blochl, *Phys. Rev. B* **1994**, 50, 17953.

[17] G. Kresse, D. Joubert, *Phys. Rev. B* **1999**, 59, 1758.

[18] S. Grimme, J. Antony, S. Ehrlich, H. Krieg, *J. Chem. Phys.* **2010**, 132, 154104.

[19] L. Zhang, Q. Zheng, Y. Xie, Z. Lan, O. V. Prezhdo, W. A. Saidi, J. Zhao, *Nano Lett.* **2018**, 18, 1592.

[20] W. Chu, W. A. Saidi, Q. Zheng, Y. Xie, Z. Lan, O. V. Prezhdo, H. Petek, J. Zhao, *J. Am. Chem. Soc.* **2016**, 138, 13740.

[21] Q. Zheng, W. A. Saidi, Y. Xie, Z. Lan, O. V. Prezhdo, H. Petek, J. Zhao, *Nano Lett.* **2017**, 17, 6435.

[22] H. M. Jaeger, S. Fischer, O. V. Prezhdo, *J. Chem. Phys.* **2012**, 137, 22a545.

[23] A. Grieder, M. C. Andrade, H. Takenaka, T. Ogitsu, L. Z. Tan, Y. Ping, *Phys. Rev. Lett.* **2025**, 135, 136301.
